# Preoperative treatment and postoperative outcomes in osteoporotic patients with vertebral fractures: a longitudinal database study

**DOI:** 10.1007/s00774-025-01639-2

**Published:** 2025-09-17

**Authors:** Ruriko Koto, Shiori Yoshida, Akihiro Nakajima, Tetsuya Miwa, Naohisa Miyakoshi

**Affiliations:** 1 Medical Science Department, Teijin Pharma Limited, 2-1, Kasumigaseki 3-chome Chiyoda-ku, Tokyo, 100-8585 Japan; 2 Clinical Development Control Department, Teijin Pharma Limited, Tokyo, Japan; 3 Department of Orthopedic Surgery, Akita University Graduate School of Medicine, Akita, Japan

**Keywords:** Osteoporosis, Anabolic agent, Vertebroplasty, Spine fusion, Spine surgery

## Abstract

**Introduction:**

This study aimed to investigate perioperative treatment and postoperative outcomes in osteoporotic patients with vertebral fractures (VFs), categorized by the type of spine surgery.

**Materials and methods:**

Patients aged ≥ 40, diagnosed with VFs and osteoporosis, with initial spine surgery between April 2015 and February 2021, were analyzed using a Japanese claims database. Time-to-event analysis was conducted for postoperative outcome. Outcome-related factors were explored with a multivariable Cox proportional hazards model.

**Results:**

The study population (*n* = 4870) consisted of 2675 patients in the percutaneous vertebroplasty (PVP) group and 2195 in the spine fusion surgery (SFS) group. Most patients had lumbar VFs, and posterior spinal fusion was common in the SFS group. Approximately 20% of patients did not receive prescriptions for osteoporosis medications during the perioperative period. Most reoperations and subsequent fractures occurred within 90 days after PVP or SFS. In the PVP group, degenerative spine disease (adjusted hazard ratio 1.34 [95% CI, 1.03–1.76]), psychotropic drugs (1.34 [1.03–1.76]), and glucocorticoid prescriptions with a mean dose of ≥ 5 to < 7.5 mg/day (2.35 [1.04–5.34]) (vs. < 1 mg/day) were associated with reoperation. In post hoc subgroup analysis by year of spine surgery, anabolic agents were associated with a lower risk of reoperation (0.48 [0.30–0.75]) in 2019 and later. In the SFS group, hyperparathyroidism and Parkinson’s disease were associated with reoperation (2.14 [1.03–4.44] and 2.10 [1.31–3.37], respectively).

**Conclusion:**

Perioperative osteoporosis medication may be suboptimal. Factors associated with postoperative outcomes must be considered, with the strategic goal of improving patient outcomes.

**Supplementary Information:**

The online version contains supplementary material available at 10.1007/s00774-025-01639-2.

## Introduction

The vertebrae are a major site of fractures in osteoporosis [1], and the incidence of such fractures increases with age [2, 3]. Patients are also at high risk of secondary fractures within 2 years after vertebral fracture (VF) [4–6]. Such fractures interfere with activities of daily living [7], decrease patient quality of life [8], and worsen survival prognosis [9, 10]. Effective prevention of VF could play a major role in reducing the burden for patients and on the healthcare system.

The standard treatment for VF consists of conservative management, including rest, bracing, and analgesics [11]. Spine surgery, such as percutaneous vertebroplasty (PVP) or spine fusion surgery (SFS), is indicated if pain does not improve, bone healing is delayed, or issues regarding spinal deformity or neurological impairment occur even under the best possible conservative management. However, few large-scale studies have reported on the characteristics of patients who undergo these surgical procedures. Additionally, poor bone health is often underestimated in patients undergoing spine surgery, resulting in inadequate treatment [12]. In the United States, one report showed that only 14.3% of osteoporotic patients who underwent SFS had received osteoporosis medications prior to surgery, and only 43% were still continuing those medications 1 year after surgery [13]. Osteoporosis treatment guidelines [14, 15] recommend medication following VF or hip fractures (HF). Additionally, the U.S. guidelines for elective spinal reconstruction surgery in adult osteoporosis patients, suggest anabolic agents as first-line medications, specifically daily teriparatide, a synthetic form of parathyroid hormone (PTH), and abaloparatide, to be administered pre- and post-operatively unless contraindicated [12]. In-depth analysis is limited, however, by the small amount of data available on the real-world use of these anabolic agents in osteoporotic patients undergoing spine surgery. In particular, we know little about how these agents are used during the perioperative period, when medication is initiated, and how long that medication is continued.

In patients undergoing PVP, subsequent fractures have been reported to occur frequently [16, 17], and many occur relatively early, within 2–3 months after surgery. In SFS, patients with poor bone health appear to be especially at risk of various complications [18, 19], and also at greater risk of requiring revision surgery [20, 21]. However, previous research has been predominantly small-scale and observational, and only a limited number of real-world studies have assessed the incidence of subsequent VF and the risk of reoperation associated with PVP and with SFS [22]. In addition, evaluation of the risk of HF after spine surgery may have significance, because patients who have undergone spine surgery may experience motor function impairment, which increases their likelihood of falling and their risk of HF [23].

Previous studies have identified several risk factors associated with subsequent fractures in osteoporotic patients after spine surgery, including low bone density after the initial surgery, cement leakage, and kyphosis [24, 25]. Although only a few studies have examined associated factors such as perioperative medications [26], some research has indicated that teriparatide can reduce subsequent fractures after vertebroplasty [27]. These findings raise the question of whether postoperative outcomes can be improved by effective perioperative medication in osteoporotic patients who experience VF and undergo either PVP or SFS.

We used Japanese administrative claims data to examine patient characteristics and perioperative osteoporosis medication for each type of surgery, to assess the incidence of reoperation and subsequent fractures (VF and HF), and to explore factors associated with reoperation and subsequent fractures in those patients. Such information, based on real-world clinical practice in Japan, is particularly meaningful because older adults make up a large percentage of the Japanese population [28] and osteoporosis is highly prevalent in patients aged 80 years and older [14, 29]. As a result, Japanese hospitals perform many surgeries on older osteoporotic patients. Analyzing these therapeutic trends in Japan, the world’s first “super-aged society” [30], can provide valuable insights for osteoporosis management strategies in other countries as they face the challenge of aging populations in the future.

## Materials and methods

### Study design and setting

We conducted a retrospective longitudinal study using administrative claims data collected from April 2014 to February 2022. The database for this study was provided by DeSC Healthcare Co., Ltd. (Tokyo, Japan), using anonymized administrative claims data from three different types of health insurance: the society-managed employment-based health insurance association (SHI) for employees of large companies and their families, national health insurance (NHI) for persons under 75 years of age and not covered by another public health system, and the latter-stage elderly healthcare system (LSEHS) for persons aged 75 years and older [31]. This database included medical information such as diagnosis codes, prescription codes, and procedure codes. The system enabled us to track medical information for specific patients, even when they visited multiple medical institutions, as long as they remained under the coverage of the same health insurance society [32].

The study was registered through the University Hospital Medical Information Network (UMIN) Clinical Trials Registry (UMIN000054550).

### Participants

We identified patients who had undergone initial spine surgery, either PVP or SFS, from April 2015 to February 2021. The initial spine surgery date/month was defined as the date/month of the first spine surgery. The index date/month was defined as 3 weeks after the initial surgery. This index date/month marked the end of the perioperative period, which will be described later. The baseline period was defined as the 12 months before the index month (Online Resource 1 [Supplemental Fig. 1]).

We selected patients who had remained continuously registered in the database for 12 months before and 1 month after the initial spine surgery month, had been diagnosed with VF within the 12-month period before the initial spine surgery month, had been diagnosed with osteoporosis during the baseline period (excluding the index month), and were 40 years of age or older at the index month. Patients with a history of spine surgery within 12 months before the initial surgery month, or those diagnosed with infectious spondylitis, metastatic or primary spinal tumors, or multiple myeloma during the baseline period were excluded. Diseases and procedures are defined in Online Resource 1 (Supplemental Table 1).

### Study measures and definitions

Study design is diagrammed in Online Resource 1 (Supplemental Fig. 1). For both PVP and SFS, the study assessed patient demographics and perioperative medication practices, occurrences of postoperative outcomes for reoperation, subsequent VF, and HF, and factors associated with the occurrence of those postoperative outcomes. The duration of the perioperative period, defined as the period from 90 days before to 21 days after the initial spine surgery, was based on guidelines for the duration of preoperative osteoporosis medications [12] and findings from previous studies [33]. Comorbidities, concomitant medications, and medication persistence are defined in Online Resource 1 (Supplemental Table 1). These definitions were used for the collection of patient demographic data.

For postoperative outcomes, reoperation was defined as SFS or PVP performed after the index date. Subsequent VF was defined as brace immobilization (by corset, etc.) or reoperation, after the index date, for a condition corresponding to the disease definition of “vertebral fracture”. Brace immobilization, surgery details, and HF are defined in Online Resource 1 (Supplemental Table 1). The starting point for calculating time to postoperative outcomes was defined as the index date. The end date for time to outcome was defined as the earliest of the following: the date of outcome onset, 1095 days after the index date (censoring), or the last day of the last month of the observation period (censoring).

### Statistical methods

Patient characteristics were described overall and for each type of spine surgery. Subgroup analyses were performed by sex and by the year of initial spine surgery. For patients who were newly prescribed anabolic agents for osteoporosis during the perioperative period, Kaplan–Meier plots were generated for medication persistence. Anabolic agents were PTH or romosozumab (abaloparatide had not yet been approved during the study period). In this study, PTH refers to PTH analogs, specifically including daily, weekly, and twice-weekly teriparatide. Kaplan–Meier plots were also generated to show time to each postoperative outcome for each type of spine surgery. To explore factors associated with postoperative outcomes, univariable and multivariable analyses were conducted using Cox’s proportional hazards models for each type of spine surgery and each postoperative outcome, with time to outcome as the objective variable and patient characteristics as explanatory variables. For multivariable analysis, explanatory variables were selected based on clinically important factors and on the results of univariable analysis and correlations between variables, with consideration for the number of events. Univariable and multivariable analyses were not performed for HF because of the small number of events.

For post hoc analysis, prescriptions of osteoporosis medications during the pre-perioperative period and the perioperative period were cross-tabulated. Factors associated with postoperative outcomes were subjected to subgroup analysis by year of initial spine surgery, using Cox’s proportional hazards model with Firth’s corrections because of the small number of events noted per variable.

In addition, second-line therapy and medication possession ratio (MPR) were evaluated in patients who were newly prescribed anabolic agents for osteoporosis during the perioperative period as post hoc analyses. Second-line therapy was assessed in patients who could be followed for 60 days after discontinuation of anabolic therapy. Within this period, we identified situations in which any osteoporosis medications other than the initially prescribed perioperative anabolic agent were administered, and calculated the numbers of each type of such medications. MPR was calculated and summarized for patients who could be followed for 365 days from the initial administration date of the anabolic agent.

All statistical analyses were performed using SAS version 9.4.

### Data availability

The datasets generated and/or analyzed during the current study were obtained from data accessed under license with DeSC Healthcare Inc. (Tokyo, Japan) and are not publicly available due to restrictions placed by DeSC Healthcare Inc. on the use of that data.

## Results

### Study population

**Fig. 1:**
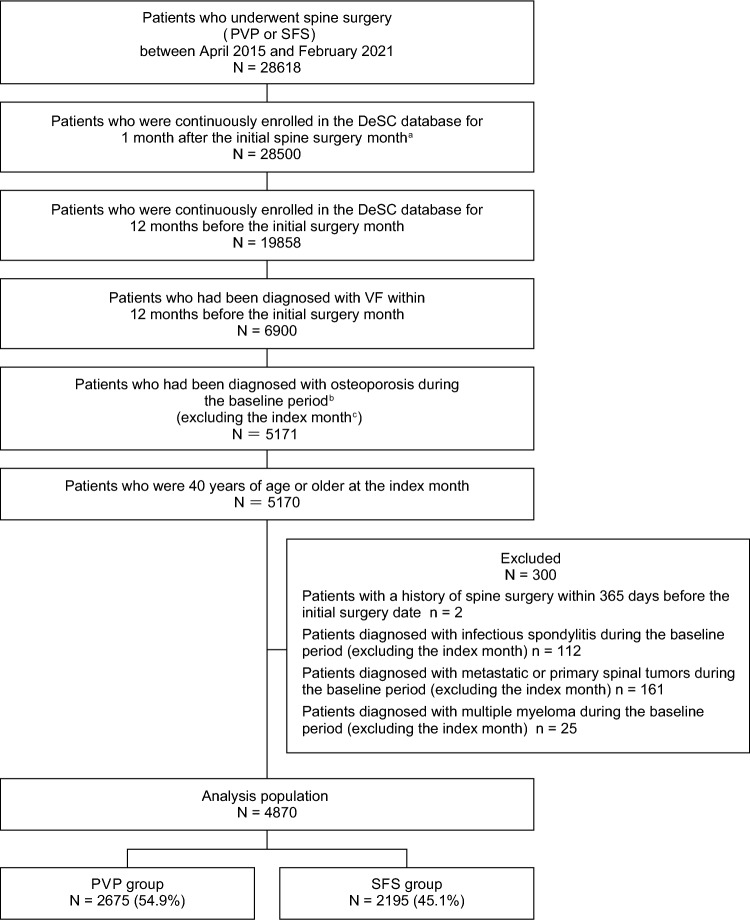
Patient disposition. *PVP* percutaneous vertebroplasty; *SFS* spine fusion surgery; *VF* vertebral fracture. ^a^ The initial surgery month within the included period. ^b^ The 12-month period before the index month. ^c^ The month 22 days after the initial surgery date

Patient disposition is shown in Fig. 1. Overall, 28,618 patients underwent spine surgery. Of these, 4870 patients were included in the analysis population: 2675 (54.9%) in the PVP group and 2195 (45.1%) in the SFS group.

### Patient demographics

Patient demographics are shown in Table 1. In the overall analysis population (*n* = 4870), the mean age ± standard deviation (SD) was 79.2 ± 6.9 years, with a higher proportion of women (75.6%). Mean age ± SD was 80.2 ± 6.7 years in the PVP group (*n* = 2675) and 78.0 ± 6.8 years in the SFS group (*n* = 2195). In total, 44.3% of patients received no prescriptions for osteoporosis medications during the pre-perioperative period, 35.4% were prescribed antiresorptive agents, and 8.1% were prescribed anabolic agents.

**Table 1:** Patient demographics (excluding comorbidities and concomitant medications)

	All	PVP group	SFS group
	*N* = 4870	*N* = 2675	*N* = 2195
Sex, *n* (%)			
Male	1190 (24.4)	623 (23.3)	567 (25.8)
Female	3680 (75.6)	2052 (76.7)	1628 (74.2)
Age, years			
Mean ± SD	79.2 ± 6.9	80.2 ± 6.7	78.0 ± 6.8
Age categories, *n* (%)			
40–69 years	425 (8.7)	178 (6.7)	247 (11.3)
70–79 years	1949 (40.0)	965 (36.1)	984 (44.8)
≥ 80 years	2496 (51.3)	1532 (57.3)	964 (43.9)
Type of insurance, *n* (%)			
SHI	31 (0.6)	8 (0.3)	23 (1.0)
NHI	1069 (22.0)	528 (19.7)	541 (24.6)
LSEHS	3770 (77.4)	2139 (80.0)	1631 (74.3)
Drug classes for pre-perioperative osteoporosis medications^a^, *n* (%)			
No	2155 (44.3)	1348 (50.4)	807 (36.8)
Anabolic agents	394 (8.1)	128 (4.8)	266 (12.1)
Parathyroid hormone	356 (7.3)	121 (4.5)	235 (10.7)
Romosozumab	38 (0.8)	7 (0.3)	31 (1.4)
Antiresorptive agents	1726 (35.4)	863 (32.3)	863 (39.3)
Bisphosphonate	1243 (25.5)	639 (23.9)	604 (27.5)
Selective estrogen receptor modulator	263 (5.4)	143 (5.3)	120 (5.5)
Anti-RANKL	220 (4.5)	81 (3.0)	139 (6.3)
Combination of two or more^b^	15 (0.3)	8 (0.3)	7 (0.3)
Bone metabolism-regulating drugs only^c^	580 (11.9)	328 (12.3)	252 (11.5)
Pre-preoperative osteoporosis medications status, *n* (%)			
Untreated^d^	2735 (56.2)	1676 (62.7)	1059 (48.2)
Previously treated	2135 (43.8)	999 (37.3)	1136 (51.8)
History of fractures other than VF, *n* (%)			
No	4105 (84.3)	2296 (85.8)	1809 (82.4)
All hip fracture	113 (2.3)	62 (2.3)	51 (2.3)
NHNV fracture	599 (12.3)	289 (10.8)	310 (14.1)
Both^e^	53 (1.1)	28 (1.0)	25 (1.1)

*LSEHS* latter-stage elderly health care system; *NHI* national health insurance; *NHNV* non-hip non-vertebral; *PVP* percutaneous vertebroplasty; *RANKL* receptor activator of nuclear factor kappa-B ligand; *SD* standard deviation; *SFS* spine fusion surgery; *SHI* society-managed employment-based health insurance association; *VF* vertebral fracture

^a^ “Anabolic agents” and “antiresorptive agents” also include patients who use those agents in combination with “bone metabolism-regulating drugs”

^b^ Excluding bone metabolism-regulating drugs

^c^ A general term that includes active vitamin D, vitamin K, and calcium

^d^ Untreated or bone metabolism-regulating drugs only

^e^ Patients with a history of both all hip fractures together with NHNV during the baseline period

The demographic background of comorbidities and concomitant medications is shown in Online Resource 1 (Supplemental Table 2). Comorbid degenerative spine disease was noted in 55.5% of patients, with lumbar spinal stenosis being the most prevalent. Notably, 71.1% of the patients in the SFS group had comorbid degenerative spine diseases, with a high prevalence of each. Among the concomitant medications of interest in this study, many patients were prescribed proton pump inhibitors, anxiolytics, and anticonvulsants: 61.0, 48.7, and 48.1%, respectively, in the SFS group.

### Treatment status during the perioperative period

Perioperative treatment status is shown in Table 2. A greater proportion of patients who underwent PVP had the procedure performed at facilities with 20–199 beds (43.3%), while more patients who underwent SFS were treated at larger hospitals with 400 or more beds (50.7%). In both spine surgery groups, lumbar fractures were the most common preoperative diagnosis. Posterior spinal fusion was the most common fusion procedure (69.9%), and the fusion length was 3 or more levels in most cases (75.9%). Approximately 20% of patients in both groups did not receive osteoporosis medications during the perioperative period. The prescription of anabolic agents was more common than that of antiresorptive agents in both groups. For patients who were first prescribed anabolic agents in the perioperative period, medication was most commonly initiated within 30 days before surgery for both groups (58.7 and 54.5%, respectively).

**Table 2:** Treatment status during the perioperative period

	PVP group	SFS group
	*N* = 2675	*N* = 2195
Index year^a^, *n* (%)		
–2018	672 (25.1)	598 (27.2)
2019–	2003 (74.9)	1597 (72.8)
No. of beds at medical institutions performing surgery, *n* (%)		
≤ 19	92 (3.4)	30 (1.4)
20–199	1159 (43.3)	368 (16.8)
200–399	651 (24.3)	684 (31.2)
≥ 400	773 (28.9)	1113 (50.7)
Missing	0 (0.0)	0 (0.0)
Types of VF^b^, *n* (%)		
Thoracic vertebrae fracture	1058 (39.6)	671 (30.6)
Lumbar fracture	1908 (71.3)	1535 (69.9)
Spinal fracture, unspecified	636 (23.8)	508 (23.1)
Details of the surgical procedure^c^, *n* (%)		
Posterior spinal fusion	–	1535 (69.9)
Anterior spinal fusion^d^	–	572 (26.1)
Combined percutaneous vertebroplasty	–	88 (4.0)
Length of fusion area, *n* (%)		
1 level	–	101 (4.6)
2 levels	–	428 (19.5)
≥ 3 levels	–	1666 (75.9)
Drug classes for perioperative osteoporosis medications^e^, *n* (%)		
No	529 (19.8)	416 (19.0)
Anabolic agents	970 (36.3)	1001 (45.6)
Parathyroid hormone	804 (30.1)	896 (40.8)
Romosozumab	166 (6.2)	105 (4.8)
Antiresorptive agents	804 (30.1)	547 (24.9)
Bisphosphonate	545 (20.4)	389 (17.7)
Selective estrogen receptor modulator	127 (4.7)	75 (3.4)
Anti-RANKL	132 (4.9)	83 (3.8)
Combination of two or more^f^	8 (0.3)	4 (0.2)
Bone metabolism-regulating drugs only^g^	364 (13.6)	227 (10.3)
Prescription start date relative to initial surgery date^h^, *n* (%)	*N* = 482	*N* = 382
≥ − 90, ≤ − 61 days	19 (3.9)	39 (10.2)
≥ − 60, ≤ − 31 days	40 (8.3)	46 (12.0)
≥ − 30, ≤ − 1 days	283 (58.7)	208 (54.5)
≥ 1, ≤ 22 days	140 (29.0)	89 (23.3)

*PVP* percutaneous vertebroplasty; *RANKL* receptor activator of nuclear factor kappa-B ligand; *SFS* spine fusion surgery; *VF* vertebral fracture

^a^ Year of initial spine surgery

^b^ If multiple fractures occurred in the 12 months prior to the month of the initial spine surgery, the fracture closest to the initial spine surgery is counted. If multiple fractures occurred in the same month, duplicate counting is used

^c^ For patients in the SFS group, spine surgeries claimed from the date of initial spine surgery to the day before the index date are counted. The details are as follows:

- Posterior spinal fusion: If this is the only procedure claimed during the evaluation period

- Anterior spinal fusion (including combined antero-posterior approaches): If no PVP was claimed during the evaluation period, but anterior spinal fusion or spinal osteotomy was claimed

- Combined PVP: If the evaluation period included a claim for PVP

^d^ Including combined antero-posterior approaches

^e^ “Anabolic agents” and “antiresorptive agents” also include patients who use those agents in combination with “bone metabolism-regulating drugs”

^f^ Excluding bone metabolism-regulating drugs

^g^ A general term that includes active vitamin D, vitamin K, and calcium

^h^ The denominator was the number of patients who were newly prescribed anabolic agents in the perioperative period. Day 1 is the day of the initial spine surgery

Prescription status for osteoporosis medications during the pre-perioperative period and the perioperative period are cross-tabulated in Online Resource 1 (Supplemental Table 3), with a large proportion of patients continuing the same medications across both periods. Of the patients prescribed anabolic agents during the pre-perioperative period in the SFS group, 85.3% continued with those agents, while 49.1% of patients who were prescribed antiresorptive agents during the pre-perioperative period continued with that same medication. Among the patients who switched medications between the pre-perioperative and perioperative periods, PTH analogs were a relatively common choice for perioperative treatment. Online Resource 1 (Supplemental Fig. 2) shows medication persistence in patients who received their first prescriptions for PTH or romosozumab during the perioperative period. Both PTH and romosozumab tended to be discontinued earlier in the SFS group than the PVP group. The results of second-line therapy among patients who were newly prescribed anabolic agents for osteoporosis during the perioperative period are presented in Online Resource 1 (Supplemental Table 4), and the MPR results are shown in Online Resource 1 (Supplemental Table 5). The proportion of patients who did not receive second-line therapy was higher in the SFS group (71.4%) than in the PVP group (63.2%), and this proportion was even higher among patients who discontinued treatment early, both in the SFS (78.4%) and PVP (68.6%) groups. Median MPR was higher in the SFS group (77.40%) than in the PVP group (70.96%).

### Subgroup analysis by patient demographics

The results of subgroup analysis for patient demographics and perioperative treatment status by sex are shown in Online Resource 1 (Supplemental Tables 6 and 7). In the SFS group, a higher proportion of men than women had a fusion length of 3 or more levels (83.6% of men, 73.2% of women). In both groups, a higher proportion of men than women were not prescribed osteoporosis medications during the perioperative period (31.8% of men and 16.1% of women for PVP, 30.2 and 15.0%, respectively, for SFS) (Online Resource 1 [Supplemental Table 7]).

### Postoperative outcomes

**Fig. 2:**
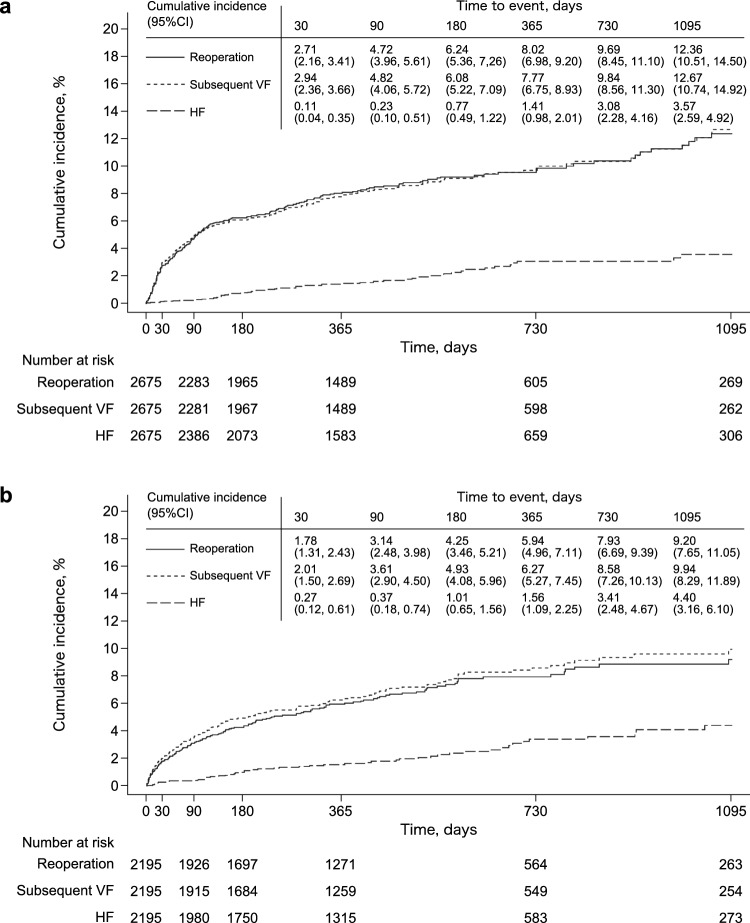
Kaplan–Meier plots for cumulative incidence of postoperative outcomes (reoperation, subsequent VF, HF) in PVP group (**a**) and SFS group (**b**). *CI* confidence interval; *VF* vertebral fracture; *HF* hip fracture; *PVP* percutaneous vertebroplasty; *SFS* spine fusion surgery

The cumulative incidence for the postoperative outcomes of reoperation, subsequent VF, and HF are shown in Fig. 2. The results suggested that reoperations and subsequent VF occurred most frequently within the first 90 days in both the PVP and SFS groups. The cumulative incidence at 3 years was 12.36 and 9.20%, respectively, for reoperations, and 12.67 and 9.94%, respectively, for subsequent VF. The incidence of HF was low in both groups: 3.57% in the PVP group and 4.40% in the SFS group at 3 years.

### Factors associated with postoperative outcomes

**Fig. 3:**
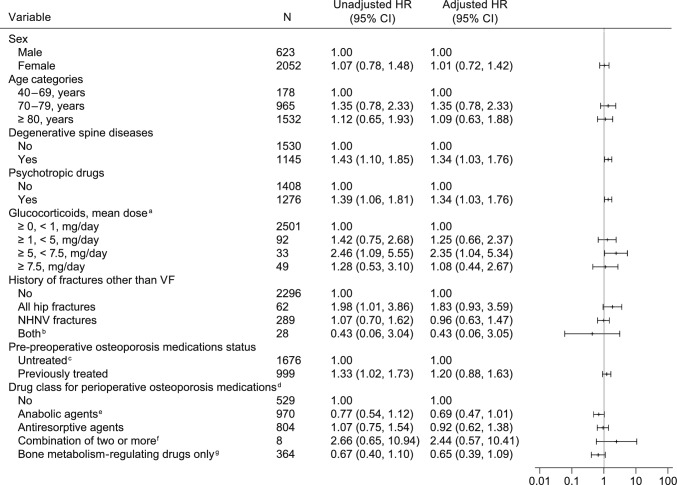
Factors associated with reoperation in the PVP group. *CI* confidence interval; *HR* hazard ratio; *NHNV* non-hip non-vertebral; *PVP* percutaneous vertebroplasty; *VF* vertebral fracture. ^a^ As prednisolone equivalent mg/day. The mean dose per day calculated from the total dose per year in patients who were prescribed oral glucocorticoids for at least 90 days during the baseline period. ^b^ Patients with a history of all hip fractures together with NHNV during the baseline period. ^c^ Untreated or bone metabolism-regulating drugs only. ^d^ “Anabolic agents” and “antiresorptive agents” also include patients who use those agents in combination with “bone metabolism-regulating drugs”. ^e^ Anabolic agents are parathyroid hormone and romosozumab. ^f^ Excluding bone metabolism-regulating drugs. ^g^ A general term that includes active vitamin D, vitamin K, and calcium

**Fig. 4:**
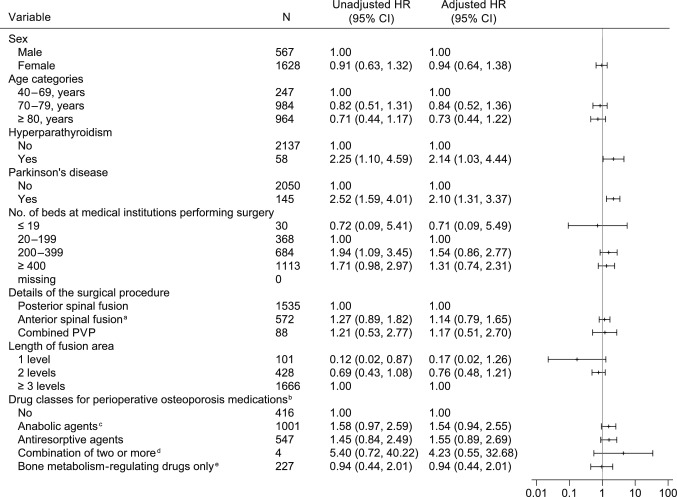
Factors associated with reoperation in the SFS group. HR, hazard ratio; PVP, percutaneous vertebroplasty; SFS, spine fusion surgery. ^a^ Including combined antero-posterior approaches. ^b^ “Anabolic agents” and “antiresorptive agents” also include patients who use those agents in combination with “bone metabolism-regulating drugs”. ^c^ Anabolic agents are parathyroid hormone and romosozumab. ^d^ Excluding bone metabolism-regulating drugs. ^e^ A general term that includes active vitamin D, vitamin K, and calcium

The factors associated with reoperation in the PVP group are shown in Fig. 3 and Online Resource 1 (Supplemental Table 8). Those factors, based on multivariable analysis, were degenerative spine diseases (HR: 1.34 [95% CI, 1.03–1.76]), psychotropic drugs (HR: 1.34 [95% CI, 1.03–1.76]), and glucocorticoid prescriptions at a mean dose of ≥ 5 to < 7.5 mg/day (HR: 2.35 [95% CI, 1.04–5.34] vs. < 1 mg/day). Anabolic agents tended to be associated with a reduced risk of reoperation (HR: 0.69 [95% CI, 0.47–1.01]). The factors associated with reoperation in the SFS group are shown in Fig. 4 and Online Resource 1 (Supplemental Table 9). Those factors were hyperparathyroidism (HR: 2.14 [95% CI, 1.03–4.44]) and Parkinson’s disease (HR: 2.10 [95% CI, 1.31–3.37]).

In the PVP group, psychotropic drugs (HR: 1.37 [95% CI, 1.05–1.79]) were associated with subsequent VF (Online Resource 1 [Supplemental Table 10]). In the SFS group, hip or knee osteoarthritis (HR: 0.50 [95% CI, 0.26–0.99]), Parkinson’s disease (HR: 1.91 [95% CI, 1.18–3.10]), and anterior spinal fusion (HR: 1.66 [95% CI, 1.18–2.33] vs. posterior spinal fusion) were associated with subsequent VF (Online Resource 1 [Supplemental Table 11]).

### Post-hoc analysis of factors associated with postoperative outcomes, by year of initial spine surgery

In the database used for this study, the proportion of patients covered by the LSEHS varied greatly by year. In particular, that proportion increased to around 80% after 2019 (Online Resource 1 [Supplemental Table 12]), suggesting notable differences before and after 2019 in the demographic characteristics, especially the proportion of older patients, of the study population. We thus conducted a subgroup analysis of factors associated with postoperative outcomes, dividing patients into those whose initial spine surgery was performed in 2018 and earlier, or in 2019 and later (Online Resource 1 [Supplemental Tables 13–16]).

The results of subgroup analysis were generally similar to the overall analysis, but with some differences. For example, in the PVP group, an association was noted between reoperation and anabolic agents in 2019 and later (HR: 0.48 [95% CI, 0.30–0.75]) (Online Resource 1 [Supplemental Table 13]), and between subsequent VF and anabolic agents in 2019 and later (HR: 0.52 [95% CI, 0.33–0.84]) (Online Resource 1 [Supplemental Table 15]), while in the SFS group, an association was noted between subsequent VF and malignant tumor in 2018 and earlier (HR: 2.30 [95% CI, 1.16–4.56]) (Online Resource 1 [Supplemental Table 16]).

## Discussion

In this study, we used real-world data from Japan to analyze perioperative treatment practices for patients who had osteoporosis complicated by VF. We found that lumbar fracture was the most common form of VF in the patients who underwent PVP or SFS. Most SFS procedures were performed across 3 or more levels and involved posterior spinal fusion. Approximately 20% of all patients and 30% of male patients were not prescribed osteoporosis medications during the perioperative period. There was notable early discontinuation among patients who were prescribed anabolic agents, suggesting that perioperative medication was suboptimal. Similarly, in a previous study, osteoporosis medications were actually provided to only a small proportion of patients who experience osteoporotic fractures [34], suggesting a discrepancy between the treatment guidelines for osteoporosis [14, 15] and actual clinical practice. The best practice guidelines for the evaluation and management of osteoporosis in adult patients undergoing elective spinal reconstruction surgery in the U.S. recommend that anabolic agents be considered as first-line therapy, and that medications should be continued for at least 2 months preoperatively and for a minimum of 8 months postoperatively [12]. In the present study, anabolic agents were prescribed more frequently than antiresorptive agents as osteoporosis medication during the perioperative period. Notably, among patients who were first prescribed an anabolic agent during the perioperative period, such medication was most commonly initiated within 1 month before surgery in both the PVP group and the SFS group. This shorter duration of preoperative medications for PVP is reasonable, given the need for timely surgical intervention after a fracture. Similarly, for SFS this abbreviated time period likely reflects the need for rapid surgical repair in cases of VF with paralysis, where patients cannot wait an extended time before surgery.

In this study, we focused on postoperative outcomes, including reoperation, subsequent VF, and HF. Previous studies have shown that patients who undergo PVP are likely to develop new adjacent or non-adjacent fractures relatively early, approximately 50–100 days after surgery [16, 17, 35]. Those results were consistent with our study findings, which showed that the incidence for new fractures ≤ 90 days after the index date (≤ 112 days after surgery) was higher than the subsequent incidence. The same trend was apparent in the SFS group. In this study, the incidence of HF was low, possibly because the study population was relatively young, with a mean age of 79.2 years, and was thus less at risk for HF. Previous epidemiological studies have shown the highest incidence of HF in patients aged 85 and older [36].

In the PVP group, the factors associated with reoperation were identified as degenerative spine diseases, the use of psychotropic drugs, and glucocorticoid prescriptions at a mean dose of ≥ 5 to < 7.5 mg/day. However, in real-world clinical practice where degenerative spine diseases are present, it is common to first perform PVP for VF, with SFS scheduled for a later time. Therefore, our findings for “reoperation” may include some surgeries that were actually planned in advance. Additionally, psychotropic drugs [37, 38] and glucocorticoids [39] are known risk factors for fractures and osteoporosis. In this study, although glucocorticoid doses of ≥ 7.5 mg/day were not identified as a risk factor, many of the patients who were prescribed high-dose glucocorticoids also had comorbidities of cardiovascular or cerebrovascular disease, often with concomitant prescriptions for anticoagulants (data not shown). It is possible that reoperation was not performed in these patients due to concerns about the physical risks of additional surgery. In the SFS group, hyperparathyroidism and Parkinson’s disease were identified as factors associated with reoperation. Those findings are supported by previous work that links hyperparathyroidism to pathological fractures due to bone resorption and decreased bone density caused by elevated PTH levels [40], and that links Parkinson’s disease to increased risk of complications and reoperation following SFS [41]. Interestingly, our study identified hip or knee osteoarthritis as a protective factor against subsequent VF in SFS (Online Resource 1 [Supplemental Table 11]). Although osteoarthritis is associated with an increased risk of falls, a meta-analysis has shown no significant correlation between osteoarthritis and fracture risk [42]. The potential role of osteoarthritis in fractures requires detailed investigation in future studies.

Because of the small number of postoperative outcome events, our study did not have sufficient power to provide a definitive answer to the question of whether perioperative osteoporosis medications were associated with postoperative outcomes. Although findings from SFS showed non-significant associations between perioperative osteoporosis medications and a higher risk of reoperation (anabolic agents: HR 1.54 [95% CI, 0.94–2.55], antiresorptive agents: HR 1.55 [95% CI, 0.89–2.69]). In the SFS group, there was a tendency for earlier discontinuation of anabolic agents. Additional analysis showed that the SFS group had a higher proportion of patients who did not receive second-line therapy than the PVP group. This proportion was even higher among patients who discontinued treatment early in both groups, especially the SFS group (Online Resource 1 [Supplemental Table 4]). These findings suggest that in the SFS group anabolic agents may have been more frequently used to promote bone fusion than to prevent subsequent fractures, and also that insufficient implementation of second-line therapy may have contributed to the need for reoperation. MPR was higher in the SFS group than in the PVP group (Online Resource 1 [Supplemental Table 5]), suggesting that a higher proportion of patients in the SFS group restarted treatment with anabolic agents after discontinuation. Of potential interest, a previous study has reported that the anabolic agent teriparatide effectively promoted bone fusion after SFS [43], but that study population consisted of patients who underwent posterolateral fusion across only 1 or 2 levels. In the present study, 75.9% of patients underwent SFS involving 3 or more levels, and the effects of perioperative medications may be less clear in these longer fusions; further detailed study is needed. Findings for PVP showed a trend toward reduced risk of reoperation in patients who received perioperative treatment with anabolic agents (HR: 0.69 [95% CI, 0.47–1.01]). Furthermore, in the subgroup analysis of patients who underwent PVP in 2019 and later, anabolic agents were significantly associated with a reduced risk of reoperation and subsequent VF (HR: 0.48 [95% CI, 0.30–0.75] and HR: 0.52 [95% CI, 0.33–0.84], respectively) (Online Resource 1 [Supplemental Tables 13 and 15]). The proportion of older adults in the database differed significantly between the periods before 2018 and after 2019. In addition, there were major changes in the market environment after 2019, including the launch of romosozumab and the introduction of generic teriparatide, which may have influenced the results from 2019 and later. In addition, although the guidelines for elective spinal reconstruction in adult patients in the United States recommend daily teriparatide and abaloparatide [12], the anabolic agents evaluated in this study were PTH analogs and romosozumab. These two agents differ in their bone metabolism dynamics: PTH analogs act through a remodeling-based mechanism for bone formation, whereas the mechanism of romosozumab is based primarily on the induction of mini-modeling [44, 45]. Given that such differences in bone metabolism dynamics may influence postoperative outcomes in patients with VFs, further investigation is needed to evaluate the treatment effects of each agent on postoperative outcomes.

The strength of this study lies in providing real-world information on approximately 5000 perioperative osteoporosis patients with VF, using a large-scale medical database from Japan. By covering data from major insurance associations, we were able to observe a highly generalizable population that included a substantial number of older adults. In addition, the features of this database ensured a high degree of traceability of outcomes.

This study has several limitations. First, studies that involve the secondary use of medical information databases have limitations regarding the validity of the definitions of diseases and outcomes. In particular, it is difficult to define a true “vertebral fracture” from a database of insurance claims data. Additionally, in this study, since many patients in the SFS group had comorbid degenerative spine diseases (Online Resource 1 [Supplemental Table 2]), it is possible that SFS was not necessarily performed solely for the treatment of osteoporotic VF. Second, since the period for identifying patients undergoing their initial surgery was set to 12 months, surgeries performed more than 12 months earlier were not identified. As a result, there may be patients included in the study for whom this spine surgery was not their first. Third, data such as the date of the VF injury, details of spine surgery procedures, severity of the patient’s condition, and information on bone density could not be obtained from the database, so these potential risk factors could not be analyzed. However, despite these limitations, this study is the first in Japan to access large-scale real-world data to provide evidence on perioperative treatment practices in osteoporotic patients with VF.

In conclusion, this study showed discrepancies between osteoporosis treatment guidelines and real-world treatment practices in osteoporotic patients with VF, suggesting that perioperative osteoporosis medication may be suboptimal in this population. Postoperative outcomes were closely associated with factors. Such factors must always be considered when working to improve patient outcomes.

## Supplementary Information

Below is the link to the electronic supplementary material.

Supplementary file1 (PDF 944 KB)

## Data Availability

The datasets generated and/or analyzed during the current study were obtained from data accessed under license with DeSC Healthcare Inc. (Tokyo, Japan) and are not publicly available due to restrictions placed by DeSC Healthcare Inc. on the use of that data.
