# Supplementary material for: Preoperative treatment and postoperative outcomes in osteoporotic patients with vertebral fractures: a longitudinal database study
Source: J Bone Miner Metab. 2025 Sep 17;43(6):649–61. doi: 10.1007/s00774-025-01639-2 (PMC12701861; doi:10.1007/s00774-025-01639-2)
Supplement: Supplementary file 1 — Supplementary file1 (PDF 944 KB) [file 774_2025_1639_MOESM1_ESM.pdf]

Supplementary Material
Journal of Bone and Mineral Metabolism

Preoperative treatment and postoperative outcomes in osteoporotic patients with vertebral fractures: a longitudinal database study

Ruriko Koto[1], Shiori Yoshida[2], Akihiro Nakajima[2], Tetsuya Miwa[1], Naohisa Miyakoshi[3]
1. Medical Science Department, Teijin Pharma Limited, Tokyo, Japan
2. Clinical Development Control Department, Teijin Pharma Limited, Tokyo, Japan
3. Department of Orthopedic Surgery, Akita University Graduate School of Medicine, Akita, Japan

Contents:

Supplemental Table 1 Definitions of diseases, procedures, outcomes, and covariates 2

A. Definitions of diseases and procedures, in inclusion and exclusion criteria 2

B. Definitions of factors including comorbidities, concomitant medications, and medication persistence, in patient demographics and treatment status 2

C. Definitions of HF, and brace immobilization and HF surgery in outcomes 6

Supplemental Table 2 Patient demographics (comorbidities and concomitant medications) 7

Supplemental Table 3 Cross-tabulation of prescription practices for osteoporosis medications during the pre-perioperative and perioperative periods 9

Supplemental Table 4 Second-line therapy in patients who first started PTH or Rmab in the perioperative period 11

Supplemental Table 5 MPR during the 365 days following new prescription in patients who first started PTH or Rmab in the perioperative period 13

Supplemental Table 6 Subgroup analysis of patient demographics by sex 14

Supplemental Table 7 Subgroup analysis of perioperative treatment status by sex 18

Supplemental Table 8 Details of factors associated with reoperation in the PVP group 20

Supplemental Table 9 Details of factors associated with reoperation in the SFS group 25

Supplemental Table 10 Factors associated with subsequent VF in the PVP group 30

Supplemental Table 11 Factors associated with subsequent VF in the SFS group 35

Supplemental Table 12 Subgroup analysis of patient demographics (sex, age, insurance type), by year of first spine surgery 40

Supplemental Table 13 Subgroup analysis of factors associated with reoperation in the PVP group, by year of first spine surgery 41

Supplemental Table 14 Subgroup analysis of factors associated with reoperation in the SFS group, by year of first spine surgery 47

Supplemental Table 15 Subgroup analysis of factors associated with subsequent VF in the PVP group, by year of first spine surgery 53

Supplemental Table 16 Subgroup analysis of factors associated with subsequent VF in the SFS group, by year of first spine surgery 59

Supplemental Fig. 1 Diagram of study design 65

Supplemental Fig. 2 Medication persistence in patients who first started PTH or Rmab in the perioperative period 66

Correspondence to:
Tetsuya Miwa t.miwa@teijin.co.jp

Supplemental Table 1 Definitions of diseases, procedures, outcomes, and covariates

A. Definitions of diseases and procedures, in inclusion and exclusion criteria

PVP
Patients for whom no appropriate SFS codes applied and only the following Claims code was applicable.
150355210

SFS
Claims codes: 150314710, 150314610, 150282610, 150282510, 150065010, 150314810, 150282750, 150369170, 150369070, 150368970, 150368870

VF
ICD-10: T08, S220, S221, S320, S321, S322, S327, S328, Claims codes: 8844320, 8842076, 8848171, 8844205, 8844274, 8850300, 8850338

Osteoporosis
ICD-10: M80, M81

Infectious spondylitis
ICD-10: M462, M463, M464, M465, G061, G062, A178

Metastatic or primary spinal tumor
ICD-10: C80, C76, D33, C412, C419, C720, C729, C794, C795, C798, C799, D166, D168, D169, D321, D329, D367, D369, D480, D487, D489

Multiple myeloma
In addition to C900 of ICD-10, the following Claims codes also apply: 2030003, 8839397, 8840039, 8842090, 8847152, 8847175, 8847250, 8847258

ICD-10, international statistical classification of diseases and related health problems 10th revision; PVP, percutaneous vertebroplasty; SFS, spine fusion surgery; VF, vertebral fracture

B. Definitions of factors including comorbidities, concomitant medications, and medication persistence, in patient demographics and treatment status

Comorbidities
Identified during the baseline period (excluding the index month)

Degenerative spine diseases
Category containing “kyphosis/scoliosis,” “cervical ossification of the posterior longitudinal ligament/ ossification of the spinal ligament,” “lumbar spondylolisthesis,” “lumbar spinal stenosis,” and “herniated lumbar disk”.

Kyphosis/scoliosis
ICD-10: M40, M41

Cervical ossification of the posterior longitudinal ligament/ossification of the spinal ligament
ICD-10: M488

Lumbar spondylolisthesis
ICD-10: M430, M431, Q762

Lumbar spinal stenosis
ICD-10: M480

Herniated lumbar disk
ICD-10: M510, M511, M512, M518, G834

Hip or knee osteoarthritis
ICD-10: M169, M179

Hyperparathyroidism
ICD-10: E210, E211, E213

Hypoparathyroidism
ICD-10: E20, E892

Hyperthyroidism
ICD-10: E05

Hypothyroidism
ICD-10: E03

Chronic kidney disease
Non-dialysis
ICD-10: E102, E112, E132, E142, I12, I13, I150, I151, N00-N08, N11, N12, N14, N15, N16, N18, N19, N25, N26, N27, N28, N391, N392, E851, D593, B52, Q60, Q61, Q62, Q63
Dialysis
Claims codes: 113002510, 114003610, 114003510, 114009310, 114009410, 114006510, 114008250, 114006610, 114009510, 140008510, 140054950, 140054850, 140008770, 140008810, 140057810, 140057910, 140058010, 140059310, 140059410, 140059510, 140060210, 140060310, 140060410, 140058110, 140058210, 140058310, 140060510, 140060610, 140060710, 140058410, 140058510, 140058610, 140060810, 140060910, 140061010, 140007710, 140007910, 140058770, 140058870, 140033770, 140058970, 140055970, 140059070, 140059170, 140029850, 140059270, 140053670, 140051110, 140051010, 140036710, 140052810, 140008170, 140052570, 140052970, 140062770

Diabetes mellitus
ICD-10: E10, E11, E12, E13, E14

Parkinson's disease
ICD-10: G20, G21, G22

Hypertension
ICD-10: I10, I11, I12, I13, I15, I16

Dyslipidemia
ICD-10: E78

Myocardial infarction
ICD-10: I21, I22, I252

Congestive heart failure
ICD-10: I099, I110, I130, I132, I255, I420, I425, I426, I427, I428, I429, I43, I50, P290

Cerebrovascular disease
ICD-10: G45, G46, H340, I60, I61, I62, I63, I64, I65, I66, I67, I68, I69

Dementia
ICD-10: F00, F01, F02, F03, F051, G30, G311

Chronic pulmonary disease
ICD-10: I278, I279, J40, J41, J42, J43, J44, J45, J46, J47, J60, J61, J62, J63, J64, J65, J66, J67, J684, J701, J703

Liver disease
Mild
ICD-10: B18, K700, K701, K702, K703, K709, K713, K714, K715, K717, K73, K74, K760, K762, K763, K764, K768, K769, Z944
Moderate to severe
ICD-10: I850, I859, I864, I982, K704, K711, K721, K729, K765, K766, K767

Rheumatologic disease
ICD-10: M05, M06, M315, M32, M33, M34, M351, M353, M360

Malignant tumor
ICD-10: C00-C14, C15-26, C30-C39, C40-C41, C43, C45-C49, C50, C51-C58, C60-C63, C64-C68, C69-C72, C73-C75, C76, C77-C80, C81, C82, C83, C84, C85, C88, C90, C91, C92, C93, C94, C95, C96, C97

Concomitant medications

Identified as drugs prescribed for 7 or more consecutive days, excluding drugs used only as needed, during the baseline period. This period excludes the index date. Does not apply to glucocorticoids.

Psychotropic drugs
Category containing “antipsychotics”, “anxiolytics”, and “antidepressants”

Antipsychotics
ATC5: N05A1 Atypical antipsychotics, N05A9 Typical antipsychotics

Anxiolytics
ATC5: N05C0 Tranquilizers, N05B1 Non-barbiturates (single agents).
Medications with the following claims codes, which include “bromisoval” in their generic name, are excluded from the analysis. 610408645, 610421000, 610431001, 610441001, 611120070, 611120120, 611120121, 611120122, 611120123, 611120125, 611120155, 620000460, 620000461, 620006112, 620008062, 620008429, 620008507

Antidepressants
ATC5: N06A4 SSRI antidepressants, N06A5 SNRI antidepressants, N06A9 Other antidepressants

Narcotics
ATC5: N02A0 Narcotics

Anticonvulsants
ATC5: N03A0 Anticonvulsants

Muscle relaxants
ATC5: M03A0 Peripheral muscle relaxants, M03B0 Central muscle relaxants

Proton pump inhibitor
Ingredients: omeprazole, omeprazole sodium, lansoprazole, rabeprazole sodium, esomeprazole magnesium hydrate, vonoprazan fumarate

Anticoagulants (warfarin and heparin)
Ingredients: warfarin potassium, heparin sodium, nadroparin calcium, enoxaparin sodium, parnaparin sodium, dalteparin sodium, ardeparin sodium, reviparin sodium, deligoparin sodium, tinzaparin sodium

Hormone replacement therapy (estrogen-only)
ATC5: G03C0 Estrogen agents (excluding G3A, G3E, and G3F)

Endocrine therapy except estrogen for breast cancer and prostate cancer
Ingredients: methyltestosterone, allylestrenol, ethinylestradiol, chlormadinone acetate, leuprorelin acetate, goserelin acetate, degarelix acetate, estramustine phosphate sodium hydrate, flutamide, bicalutamide, enzalutamide, apalutamide, darolutamide, abiraterone acetate, medroxyprogesterone acetate, mepitiostane, letrozole, tamoxifen citrate, toremifene citrate, fulvestrant, anastrozole, exemestane

Glucocorticoids (oral only)
Mean dose for patients who were prescribed oral glucocorticoids (excluding those used only as needed) for at least 90 days during the baseline period
Ingredients: dexamethasone acetate, dexamethasone palmitate, dexamethasone metasulfobenzoate sodium, dexamethasone sodium phosphate, triamcinolone acetonide, hydrocortisone sodium succinate, hydrocortisone sodium phosphate, prednisolone sodium succinate, betamethasone acetate and betamethasone sodium phosphate, betamethasone sodium phosphate, methylprednisolone acetate, methylprednisolone sodium succinate, cortisone acetate, dexamethasone, triamcinolone, hydrocortisone, hydrocortisone acetate, prednisolone, betamethasone, methylprednisolone, betamethasone and *d*-chlorpheniramine maleate, prednisolone sodium phosphate

Osteoporosis medications

PTH
Ingredients: teriparatide, teriparatide acetate

Rmab
Ingredients: romosozumab

BP
Ingredients: alendronate sodium hydrate[a], etidronate disodium, minodronic acid hydrate, sodium risedronate hydrate, ibandronate

sodium hydrate, zoledronic acid hydrate intravenous infusion (5mg)

SERM
Ingredients: raloxifene hydrochloride, bazedoxifene acetate

Anti-RANKL
Ingredients: denosumab

Bone metabolism-regulating drug (active vitamin D, vitamin K, calcium)
Ingredients: calcitriol[b], alfacalcidol, eldecalcitol, menatetrenone capsule (15mg), dibasic calcium phosphate hydrate, calcium L-aspartate hydrate

Medication persistence for PTH and Rmab

Treatment persistence for continuation of a drug prescription is defined below.

1. The prescription start date is defined as the date of the first prescription start date during the perioperative period.
2. If the interval between each prescription period (“prescription date” to “prescription date + [number of vials/kits prescribed × days per vial/kit] − 1”) is within the gap period, treatment is considered continuous, and the prescription periods are linked. The total prescription period is defined as the duration from the start date of the first prescription to the end date of the last linked prescription period. Days per vial/kit and gap periods are detailed below for each drug*. Changes of drugs within the same drug class or multiple prescriptions are considered continuous treatment.
   * Number of days required to assess medication persistence
     Days per vial/kit[c]/Gap period[d]
     Teriparatide (genetical recombination) subcutaneous injection kits 600μg: 28 days/60 days
     Teriparatide acetate injection 56.5μg: 7 days /14 days
     Teriparatide acetate injection 28.2μg autoinjector: 4 days /60 days
     Romosozumab (genetical recombination) subcutaneous injection 105mg syringes: 14 days /60 days
3. The end date for continuation of a drug prescription is defined as the earliest of the following:
   A) The end date of the total prescription period
   B) The day before the start date of a prescription for a different drug class (BP, SERMs, anti-RANKL, or PTH/Rmab)
   C) 730 days after the prescription start date (censoring)
   D) The last day of the month of the observation period end date (censoring)

History of fractures other than VF

All hip fracture
ICD-10: S324 (S3240, S3241), S720 (S7200, S7201), S721 (S7210, S7211), S722 (S7220, S7221)

NHNV fractures
ICD-10: S222 (S2220), S223 (S2230, S2231), S224 (S2240, S2241), S225 (S225, S2250, S2251), S323 (S3230, S3231), S325 (S3250, S3251), S327 (S3270, S3271), S328 (S3280, S3281), S422 (S4220, S4221), S424 (S4240, S4241), S520 (S5200, S5201), S521 (S5210, S5211), S523 (S5230, S5231), S525 (S5250, S5251), S526 (S5260, S5261), S723 (S7230, S7231), S724 (S7240, S7241), S727 (S7270, S7271), S729 (S7290, S7291), S820 (S8200, S8201), S821 (S8210, S8211), S920 (S9200, S9201)

Types of VF

Thoracic vertebrae fracture
ICD-10: S220, S221

Lumbar fracture
ICD-10: S320, S321, S322, S327, S328

Spinal fracture, unspecified
ICD-10: T08
Claims code: 8844320, 8842076, 8848171, 8844205, 8844274, 8850300, 8850338

Details of the surgical procedure

Posterior spinal fusion
Only one of the following codes was billed from the date of the initial spine surgery to the day before the index date
Claims code: 150314610, 150282610, 150065010, 150369070, 150368970

Anterior spinal fusion (including combined antero-posterior approaches)
Not classified as “combined PVP”, and one of the following codes has been billed from the date of the initial spine surgery to the day before the index date
Claims code: 150314710, 150282510, 150314810, 150282750, 150369170, 150368870, 150064610

Combined PVP
The following codes have been billed from the date of the initial spine surgery to the day before the index date
Claims code: 150355210

ATC, anatomical therapeutic chemical; BP, bisphosphonate; ICD-10, international statistical classification of diseases and related health problems 10th revision; NHNV, non-hip non-vertebral; PTH, parathyroid hormone; PVP, percutaneous vertebroplasty; RANKL, receptor activator of nuclear factor kappa-B ligand; Rmab, romosozumab; SERM, selective estrogen receptor modulator; SNRI, serotonin noradrenaline reuptake inhibitor; SSRI, selective serotonin reuptake inhibitor; VF, vertebral fracture

[a] Excluding drugs that contain “alendronate injection (5mg, 10mg) ” in the drug name

[b] Excluding intravenous formulations

[c] The number of days per vial/kit was based on the package insert for that drug

[d] The gap period was set at approximately twice the median interval between outpatient prescriptions, and was based on input from clinicians

C. Definition of HF, and brace immobilization and HF surgery in outcomes

HF

Defined as meeting any of the following conditions.

1. During baseline period (excluding index date), HF surgery was not claimed on a receipt also containing a billing code included in the disease definition of HF[a];
   After index date, HF surgery was newly claimed on a receipt also containing a billing code included in the disease definition of HF
2. During baseline period (excluding index date), HF surgery was claimed on a receipt also containing a billing code included in the disease definition of HF;
   From 180 days after the final date for surgery during the baseline period, HF surgery was newly claimed on a receipt also containing a billing code included in the disease definition of HF

Brace immobilization (corset, etc.)

Claims code: 140043410, 140043530, 140043630, 140043750, 140043850, 140047410

HF surgery

Claims code: 150016710, 150017310, 150018310, 150019210, 150028810, 150029810, 150042710, 150048310, 150049510, 150060410, 150289110, 150289210, 150289810, 150352210, 150384510

ICD-10, international statistical classification of diseases and related health problems 10th revision; HF, hip fractures

[a] Billing codes included in the disease definition of HF are shown in Supplemental Table 1B under the category of “All hip fracture”

Supplemental Table 2 Patient demographics (comorbidities and concomitant medications)

	All<br>N = 4870	PVP group<br>N = 2675	SFS group<br>N = 2195
**Comorbidities, n (%)**			
Degenerative spine diseases	2705 (55.5)	1145 (42.8)	1560 (71.1)
Kyphosis/scoliosis	494 (10.1)	137 (5.1)	357 (16.3)
Cervical ossification of the posterior longitudinal ligament /ossification of the spinal ligament	78 (1.6)	19 (0.7)	59 (2.7)
Lumbar spondylolisthesis	665 (13.7)	190 (7.1)	475 (21.6)
Lumbar spinal stenosis	2322 (47.7)	923 (34.5)	1399 (63.7)
Herniated lumber disk	464 (9.5)	191 (7.1)	273 (12.4)
Hip or knee osteoarthritis	465 (9.5)	209 (7.8)	256 (11.7)
Hyperparathyroidism	102 (2.1)	44 (1.6)	58 (2.6)
Hypoparathyroidism	3 (0.1)	2 (0.1)	1 (0.0)
Hyperthyroidism	117 (2.4)	59 (2.2)	58 (2.6)
Hypothyroidism	411 (8.4)	209 (7.8)	202 (9.2)
Chronic kidney disease	1033 (21.2)	538 (20.1)	495 (22.6)
Non-dialysis[a]	958 (19.7)	502 (18.8)	456 (20.8)
Dialysis	75 (1.5)	36 (1.3)	39 (1.8)
Diabetes mellitus	2037 (41.8)	1121 (41.9)	916 (41.7)
Parkinson's disease	302 (6.2)	157 (5.9)	145 (6.6)
Hypertension	3614 (74.2)	1957 (73.2)	1657 (75.5)
Dyslipidemia	2812 (57.7)	1521 (56.9)	1291 (58.8)
Myocardial infarction	165 (3.4)	90 (3.4)	75 (3.4)
Congestive heart failure	1587 (32.6)	854 (31.9)	733 (33.4)
Cerebrovascular disease	1471 (30.2)	835 (31.2)	636 (29.0)
Dementia	580 (11.9)	366 (13.7)	214 (9.7)
Chronic pulmonary disease	1443 (29.6)	805 (30.1)	638 (29.1)
Liver disease	1250 (25.7)	651 (24.3)	599 (27.3)
Mild[b]	1206 (24.8)	622 (23.3)	584 (26.6)
Moderate or severe	44 (0.9)	29 (1.1)	15 (0.7)
Rheumatologic disease	511 (10.5)	234 (8.7)	277 (12.6)
Malignant tumor	749 (15.4)	438 (16.4)	311 (14.2)
**Charlson Comorbidity Index**			
Mean ± SD	2.4 ± 2.1	2.4 ± 2.1	2.5 ± 2.2

0–2, n (%)	2785 (57.2)	1542 (57.6)	1243 (56.6)
≥ 3, n (%)	2085 (42.8)	1133 (42.4)	952 (43.4)
**Concomitant medications, n (%)**			
Psychotropic drugs	2577 (52.9)	1267 (47.4)	1310 (59.7)
Antipsychotics	571 (11.7)	248 (9.3)	323 (14.7)
Anxiolytics	2126 (43.7)	1056 (39.5)	1070 (48.7)
Antidepressants	890 (18.3)	367 (13.7)	523 (23.8)
Narcotics	51 (1.0)	30 (1.1)	21 (1.0)
Anticonvulsants	1668 (34.3)	612 (22.9)	1056 (48.1)
Muscle relaxants	619 (12.7)	325 (12.1)	294 (13.4)
Proton pump inhibitor	2739 (56.2)	1400 (52.3)	1339 (61.0)
Anticoagulants (warfarin and heparin)	327 (6.7)	154 (5.8)	173 (7.9)
Hormone replacement therapy (estrogen-only)	30 (0.6)	18 (0.7)	12 (0.5)
Endocrine therapy except estrogen for breast cancer and prostate cancer	105 (2.2)	62 (2.3)	43 (2.0)
**Glucocorticoids mean dose[c], n (%)**			
0 ≥, < 1, mg/day	4476 (91.9)	2501 (93.5)	1975 (90.0)
1 ≥, < 5, mg/day	204 (4.2)	92 (3.4)	112 (5.1)
5 ≥, < 7.5, mg/day	84 (1.7)	33 (1.2)	51 (2.3)
≥ 7.5, mg/day	106 (2.2)	49 (1.8)	57 (2.6)

PVP, percutaneous vertebroplasty; SD, standard deviation; SFS, spine fusion surgery

[a] Patients with chronic kidney disease, excluding dialysis

[b] Patients with mild liver disease, excluding moderate or severe liver disease

[c] As prednisolone equivalent mg/day. The mean dose per day calculated from the total dose per year in patients who were prescribed oral glucocorticoids for at least 90 days during the baseline period

Supplemental Table 3 Cross-tabulation of prescription practices for osteoporosis medications during the pre-perioperative and perioperative periods

	Drug classes for pre-perioperative osteoporosis medications									
	No	Anabolic agents			Antiresorptive agents				Combination of two or more[a]	Bone metabolism -regulating drugs only[b]
Drug classes for perioperative osteoporosis medications			PTH	Rmab		BP	SERM	Anti-RANKL		
**PVP group (N = 2675)**	N = 1348	N = 128	N = 121	N = 7	N = 863	N = 639	N = 143	N = 81	N = 8	N = 328
No, n (%)	444 (32.9)	6 (4.7)	6 (5.0)	0 (0.0)	48 (5.6)	34 (5.3)	7 (4.9)	7 (8.6)	0 (0.0)	31 (9.5)
Anabolic agents, n (%)	482 (35.8)	107 (83.6)	100 (82.6)	7 (100.0)	275 (31.9)	225 (35.2)	32 (22.4)	18 (22.2)	0 (0.0)	106 (32.3)
PTH	401 (29.7)	98 (76.6)	97 (80.2)	1 (14.3)	223 (25.8)	182 (28.5)	27 (18.9)	14 (17.3)	0 (0.0)	82 (25.0)
Rmab	81 (6.0)	9 (7.0)	3 (2.5)	6 (85.7)	52 (6.0)	43 (6.7)	5 (3.5)	4 (4.9)	0 (0.0)	24 (7.3)
Antiresorptive agents, n (%)	246 (18.2)	11 (8.6)	11 (9.1)	0 (0.0)	490 (56.8)	355 (55.6)	101 (70.6)	34 (42.0)	4 (50.0)	53 (16.2)
BP	156 (11.6)	6 (4.7)	6 (5.0)	0 (0.0)	349 (40.4)	334 (52.3)	11 (7.7)	4 (4.9)	1 (12.5)	33 (10.1)
SERM	31 (2.3)	1 (0.8)	1 (0.8)	0 (0.0)	86 (10.0)	6 (0.9)	80 (55.9)	0 (0.0)	3 (37.5)	6 (1.8)
Anti-RANKL	59 (4.4)	4 (3.1)	4 (3.3)	0 (0.0)	55 (6.4)	15 (2.3)	10 (7.0)	30 (37.0)	0 (0.0)	14 (4.3)
Combination of two or more[a], n (%)	0 (0.0)	0 (0.0)	0 (0.0)	0 (0.0)	4 (0.5)	2 (0.3)	2 (1.4)	0 (0.0)	4 (50.0)	0 (0.0)
Bone metabolism-regulating drugs only[b], n (%)	176 (13.1)	4 (3.1)	4 (3.3)	0 (0.0)	46 (5.3)	23 (3.6)	1 (0.7)	22 (27.2)	0 (0.0)	138 (42.1)
**SFS group (N = 2195)**	N = 807	N = 266	N = 235	N = 31	N = 863	N = 604	N = 120	N = 139	N = 7	N = 252
No, n (%)	309 (38.3)	15 (5.6)	12 (5.1)	3 (9.7)	67 (7.8)	36 (6.0)	8 (6.7)	23 (16.5)	0 (0.0)	25 (9.9)
Anabolic agents, n (%)	382 (47.3)	227 (85.3)	205 (87.2)	22 (71.0)	300 (34.8)	223 (36.9)	44 (36.7)	33 (23.7)	0 (0.0)	92 (36.5)
PTH	337 (41.8)	203 (76.3)	201 (85.5)	2 (6.5)	276 (32.0)	206 (34.1)	42 (35.0)	28 (20.1)	0 (0.0)	80 (31.7)
Rmab	45 (5.6)	24 (9.0)	4 (1.7)	20 (64.5)	24 (2.8)	17 (2.8)	2 (1.7)	5 (3.6)	0 (0.0)	12 (4.8)
Antiresorptive agents, n (%)	78 (9.7)	19 (7.1)	13 (5.5)	6 (19.4)	424 (49.1)	321 (53.1)	64 (53.3)	39 (28.1)	4 (57.1)	22 (8.7)
BP	51 (6.3)	12 (4.5)	9 (3.8)	3 (9.7)	312 (36.2)	306 (50.7)	5 (4.2)	1 (0.7)	2 (28.6)	12 (4.8)

SERM	9 (1.1)	2 (0.8)	2 (0.9)	0 (0.0)	61 (7.1)	3 (0.5)	58 (48.3)	0 (0.0)	1 (14.3)	2 (0.8)
Anti RANKL	18 (2.2)	5 (1.9)	2 (0.9)	3 (9.7)	51 (5.9)	12 (2.0)	1 (0.8)	38 (27.3)	1 (14.3)	8 (3.2)
Combination of two or more[a] , n (%)	0 (0.0)	0 (0.0)	0 (0.0)	0 (0.0)	2 (0.2)	0 (0.0)	2 (1.7)	0 (0.0)	2 (28.6)	0 (0.0)
Bone metabolism-regulating drugs only[b], n (%)	38 (4.7)	5 (1.9)	5 (2.1)	0 (0.0)	70 (8.1)	24 (4.0)	2 (1.7)	44 (31.7)	1 (14.3)	113 (44.8)

BP, bisphosphonate; PTH, parathyroid hormone; PVP, percutaneous vertebroplasty; RANKL, receptor activator of nuclear factor kappa-B ligand; Rmab, romosozumab; SERM, selective estrogen receptor modulator; SFS, spine fusion surgery

[a] Excluding bone metabolism-regulating drugs

[b] A general term that includes active vitamin D, vitamin K, and calcium

Supplemental Table 4 Second-line therapy in patients who first started PTH or Rmab in the perioperative period

	All			Discontinuation within 90 days			Discontinuation after 90 days		
	All	PVP group	SFS group	All	PVP group	SFS group	All	PVP group	SFS group
	N = 585	N = 323	N = 262	N = 306	N = 153	N = 153	N = 279	N = 170	N = 109
**Second-line therapy after anabolic agents[a], n (%)**									
No	391 (66.8)	204 (63.2)	187 (71.4)	225 (73.5)	105 (68.6)	120 (78.4)	166 (59.5)	99 (58.2)	67 (61.5)
Yes	194 (33.2)	119 (36.8)	75 (28.6)	81 (26.5)	48 (31.4)	33 (21.6)	113 (40.5)	71 (41.8)	42 (38.5)
Second-line osteoporosis medications[b]									
BP	114 (58.8)	69 (58.0)	45 (60.0)	47 (58.0)	28 (58.3)	19 (57.6)	67 (59.3)	41 (57.7)	26 (61.9)
Anti-RANKL	54 (27.8)	34 (28.6)	20 (26.7)	17 (21.0)	9 (18.8)	8 (24.2)	37 (32.7)	25 (35.2)	12 (28.6)
SERM	9 (4.6)	6 (5.0)	3 (4.0)	6 (7.4)	5 (10.4)	1 (3.0)	3 (2.7)	1 (1.4)	2 (4.8)
Rmab	14 (7.2)	8 (6.7)	6 (8.0)	9 (11.1)	5 (10.4)	4 (12.1)	5 (4.4)	3 (4.2)	2 (4.8)
PTH	3 (1.5)	2 (1.7)	1 (1.3)	2 (2.5)	1 (2.1)	1 (3.0)	1 (0.9)	1 (1.4)	0 (0.0)
**Second-line therapy after PTH[c], n (%)**									
No	351 (69.4)	179 (64.6)	172 (75.1)	200 (73.8)	93 (67.9)	107 (79.9)	151 (64.3)	86 (61.4)	65 (68.4)
Yes	155 (30.6)	98 (35.4)	57 (24.9)	71 (26.2)	44 (32.1)	27 (20.1)	84 (35.7)	54 (38.6)	30 (31.6)
Second-line osteoporosis medications[d]									
BP	92 (59.4)	55 (56.1)	37 (64.9)	42 (59.2)	25 (56.8)	17 (63.0)	50 (59.5)	30 (55.6)	20 (66.7)
Anti-RANKL	40 (25.8)	29 (29.6)	11 (19.3)	14 (19.7)	9 (20.5)	5 (18.5)	26 (31.0)	20 (37.0)	6 (20.0)
SERM	9 (5.8)	6 (6.1)	3 (5.3)	6 (8.5)	5 (11.4)	1 (3.7)	3 (3.6)	1 (1.9)	2 (6.7)
Rmab	14 (9.0)	8 (8.2)	6 (10.5)	9 (12.7)	5 (11.4)	4 (14.8)	5 (6.0)	3 (5.6)	2 (6.7)
PTH	0 (0.0)	0 (0.0)	0 (0.0)	0 (0.0)	0 (0.0)	0 (0.0)	0 (0.0)	0 (0.0)	0 (0.0)
**Second-line therapy after Rmab[e], n (%)**									
No	40 (50.6)	25 (54.3)	15 (45.5)	25 (71.4)	12 (75.0)	13 (68.4)	15 (34.1)	13 (43.3)	2 (14.3)

Yes	39 (49.4)	21 (45.7)	18 (54.5)	10 (28.6)	4 (25.0)	6 (31.6)	29 (65.9)	17 (56.7)	12 (85.7)
Second-line osteoporosis medications[f]									
BP	22 (56.4)	14 (66.7)	8 (44.4)	5 (50.0)	3 (75.0)	2 (33.3)	17 (58.6)	11 (64.7)	6 (50.0)
Anti-RANKL	14 (35.9)	5 (23.8)	9 (50.0)	3 (30.0)	0 (0.0)	3 (50.0)	11 (37.9)	5 (29.4)	6 (50.0)
SERM	0 (0.0)	0 (0.0)	0 (0.0)	0 (0.0)	0 (0.0)	0 (0.0)	0 (0.0)	0 (0.0)	0 (0.0)
Rmab	0 (0.0)	0 (0.0)	0 (0.0)	0 (0.0)	0 (0.0)	0 (0.0)	0 (0.0)	0 (0.0)	0 (0.0)
PTH	3 (7.7)	2 (9.5)	1 (5.6)	2 (20.0)	1 (25.0)	1 (16.7)	1 (3.4)	1 (5.9)	0 (0.0)

For patients who could be followed for 60 days after the end date for continuation of the anabolic agents, prescriptions for osteoporosis medications other than the index agent (excluding vitamins and calcium supplements) were identified within the 60-day period following the end of the treatment continuation period.
BP, bisphosphonate; PTH, parathyroid hormone; PVP, percutaneous vertebroplasty; RANKL, receptor activator of nuclear factor kappa-B ligand; Rmab, romosozumab; SERM, selective estrogen receptor modulator; SFS, spine fusion surgery
[a] Anabolic agents: PTH and Rmab
[b] Denominator for this proportion: number of patients listed as "Yes" for "Second-line therapy after anabolic agents"
[c] Denominator for this proportion: number of patients who first started PTH in the perioperative period
[d] Denominator for this proportion: number of patients listed as "Yes" for "Second-line therapy after PTH"
[e] Denominator for this proportion: number of patients who first started Rmab in the perioperative period
[f] Denominator for this proportion: number of patients listed as "Yes" for "Second-line therapy after Rmab"

Supplemental Table 5 MPR during the 365 days following new prescription in patients who first started PTH or Rmab in the perioperative period

	All<br>N = 592	PVP group<br>N = 336	SFS group<br>N = 256
**MPR for anabolic agents[a]**			
n[b]	592	336	256
Mean ± SD, %	63.00 ± 36.24	61.73 ± 36.21	64.68 ± 36.29
Median (IQR), %	76.16 (26.16, 93.56)	70.96 (25.75, 94.11)	77.40 (26.30, 92.74)
Min, Max, %	1.9, 144.9	1.9, 117.3	1.9, 144.9
**MPR for PTH**			
n[b]	526	302	224
Mean ± SD, %	63.20 ± 36.88	61.14 ± 37.16	65.99 ± 36.40
Median (IQR), %	75.48 (24.93, 96.44)	70.68 (23.01, 96.44)	78.77 (28.63, 96.03)
Min, Max, %	1.9, 144.9	1.9, 117.3	1.9, 144.9
**MPR for Rmab**			
n[b]	66	34	32
Mean ± SD, %	61.41 ± 30.88	66.97 ± 26.14	55.50 ± 34.66
Median (IQR), %	76.71 (38.36, 88.77)	79.86 (46.03, 90.41)	76.44 (11.51, 84.38)
Min, Max, %	7.7, 92.1	7.7, 92.1	7.7, 92.1

IQR, interquartile range; MPR, medication possession ratio; PTH, parathyroid hormone; PVP, percutaneous vertebroplasty; Rmab, romosozumab; SD, standard deviation; SFS, spine fusion surgery

[a] Anabolic agents: PTH and Rmab

[b] Patients who were first prescribed the medication during the perioperative period and could be followed for at least 365 days from the start of treatment

Supplemental Table 6 Subgroup analysis of patient demographics by sex

	All		PVP group		SFS group	
	Male<br>N = 1190	Female<br>N = 3680	Male<br>N = 623	Female<br>N = 2052	Male<br>N = 567	Female<br>N = 1628
**Age, years**						
Mean ± SD	79.5 ± 7.1	79.1 ± 6.8	80.8 ± 6.7	80.0 ± 6.7	78.1 ± 7.2	77.9 ± 6.7
**Age categories, n (%)**						
40–69 years	109 (9.2)	316 (8.6)	39 (6.3)	139 (6.8)	70 (12.3)	177 (10.9)
70–79 years	414 (34.8)	1535 (41.7)	194 (31.1)	771 (37.6)	220 (38.8)	764 (46.9)
≥ 80 years	667 (56.1)	1829 (49.7)	390 (62.6)	1142 (55.7)	277 (48.9)	687 (42.2)
**Type of insurance, n (%)**						
SHI	13 (1.1)	18 (0.5)	5 (0.8)	3 (0.1)	8 (1.4)	15 (0.9)
NHI	221 (18.6)	848 (23.0)	95 (15.2)	433 (21.1)	126 (22.2)	415 (25.5)
LSEHS	956 (80.3)	2814 (76.5)	523 (83.9)	1616 (78.8)	433 (76.4)	1198 (73.6)
**Comorbidities**, **n (%)**						
Degenerative spine diseases	620 (52.1)	2085 (56.7)	271 (43.5)	874 (42.6)	349 (61.6)	1211 (74.4)
Kyphosis/scoliosis	82 (6.9)	412 (11.2)	27 (4.3)	110 (5.4)	55 (9.7)	302 (18.6)
Cervical ossification of the posterior longitudinal ligament/ossification of the spinal ligament	23 (1.9)	55 (1.5)	5 (0.8)	14 (0.7)	18 (3.2)	41 (2.5)
Lumbar spondylolisthesis	101 (8.5)	564 (15.3)	27 (4.3)	163 (7.9)	74 (13.1)	401 (24.6)
Lumbar spinal stenosis	529 (44.5)	1793 (48.7)	219 (35.2)	704 (34.3)	310 (54.7)	1089 (66.9)
Herniated lumber disk	121 (10.2)	343 (9.3)	46 (7.4)	145 (7.1)	75 (13.2)	198 (12.2)
Hip or knee osteoarthritis	63 (5.3)	402 (10.9)	25 (4.0)	184 (9.0)	38 (6.7)	218 (13.4)
Hyperparathyroidism	43 (3.6)	59 (1.6)	14 (2.2)	30 (1.5)	29 (5.1)	29 (1.8)
Hypoparathyroidism	0 (0.0)	3 (0.1)	0 (0.0)	2 (0.1)	0 (0.0)	1 (0.1)

Hyperthyroidism	17 (1.4)	100 (2.7)	11 (1.8)	48 (2.3)	6 (1.1)	52 (3.2)
Hypothyroidism	82 (6.9)	329 (8.9)	37 (5.9)	172 (8.4)	45 (7.9)	157 (9.6)
Chronic kidney disease	343 (28.8)	690 (18.8)	162 (26.0)	376 (18.3)	181 (31.9)	314 (19.3)
Non-dialysis[a]	302 (25.4)	656 (17.8)	145 (23.3)	357 (17.4)	157 (27.7)	299 (18.4)
Dialysis	41 (3.4)	34 (0.9)	17 (2.7)	19 (0.9)	24 (4.2)	15 (0.9)
Diabetes mellitus	569 (47.8)	1468 (39.9)	300 (48.2)	821 (40.0)	269 (47.4)	647 (39.7)
Parkinson's disease	84 (7.1)	218 (5.9)	40 (6.4)	117 (5.7)	44 (7.8)	101 (6.2)
Hypertension	872 (73.3)	2742 (74.5)	441 (70.8)	1516 (73.9)	431 (76.0)	1226 (75.3)
Dyslipidemia	581 (48.8)	2231 (60.6)	297 (47.7)	1224 (59.6)	284 (50.1)	1007 (61.9)
Myocardial infarction	71 (6.0)	94 (2.6)	36 (5.8)	54 (2.6)	35 (6.2)	40 (2.5)
Congestive heart failure	450 (37.8)	1137 (30.9)	226 (36.3)	628 (30.6)	224 (39.5)	509 (31.3)
Cerebrovascular disease	431 (36.2)	1040 (28.3)	227 (36.4)	608 (29.6)	204 (36.0)	432 (26.5)
Dementia	152 (12.8)	428 (11.6)	86 (13.8)	280 (13.6)	66 (11.6)	148 (9.1)
Chronic pulmonary disease	378 (31.8)	1065 (28.9)	213 (34.2)	592 (28.8)	165 (29.1)	473 (29.1)
Liver disease	335 (28.2)	915 (24.9)	153 (24.6)	498 (24.3)	182 (32.1)	417 (25.6)
Mild[b]	319 (26.8)	887 (24.1)	144 (23.1)	478 (23.3)	175 (30.9)	409 (25.1)
Moderate or severe	16 (1.3)	28 (0.8)	9 (1.4)	20 (1.0)	7 (1.2)	8 (0.5)
Rheumatologic disease	91 (7.6)	420 (11.4)	37 (5.9)	197 (9.6)	54 (9.5)	223 (13.7)
Malignant tumor	326 (27.4)	423 (11.5)	192 (30.8)	246 (12.0)	134 (23.6)	177 (10.9)
**Charlson Comorbidity Index**						
Mean ± SD	3.0 ± 2.3	2.3 ± 2.0	2.9 ± 2.3	2.3 ± 2.0	3.0 ± 2.4	2.3 ± 2.0
0–2, n (%)	563 (47.3)	2222 (60.4)	293 (47.0)	1249 (60.9)	270 (47.6)	973 (59.8)
≥ 3, n (%)	627 (52.7)	1458 (39.6)	330 (53.0)	803 (39.1)	297 (52.4)	655 (40.2)
**Concomitant medications, n (%)**						

Psychotropic drugs	575 (48.3)	2002 (54.4)	268 (43.0)	999 (48.7)	307 (54.1)	1003 (61.6)
Antipsychotics	130 (10.9)	441 (12.0)	48 (7.7)	200 (9.7)	82 (14.5)	241 (14.8)
Anxiolytics	476 (40.0)	1650 (44.8)	218 (35.0)	838 (40.8)	258 (45.5)	812 (49.9)
Antidepressants	198 (16.6)	692 (18.8)	85 (13.6)	282 (13.7)	113 (19.9)	410 (25.2)
Narcotics	12 (1.0)	39 (1.1)	6 (1.0)	24 (1.2)	6 (1.1)	15 (0.9)
Anticonvulsants	376 (31.6)	1292 (35.1)	138 (22.2)	474 (23.1)	238 (42.0)	818 (50.2)
Muscle relaxants	153 (12.9)	466 (12.7)	72 (11.6)	253 (12.3)	81 (14.3)	213 (13.1)
Proton pump inhibitor	676 (56.8)	2063 (56.1)	322 (51.7)	1078 (52.5)	354 (62.4)	985 (60.5)
Anticoagulants (warfarin and heparin)	103 (8.7)	224 (6.1)	42 (6.7)	112 (5.5)	61 (10.8)	112 (6.9)
Hormone replacement therapy (estrogen-only)	0 (0.0)	30 (0.8)	0 (0.0)	18 (0.9)	0 (0.0)	12 (0.7)
Endocrine therapy except estrogen for breast cancer and prostate cancer	76 (6.4)	29 (0.8)	51 (8.2)	11 (0.5)	25 (4.4)	18 (1.1)
**Glucocorticoids mean dose[c], n (%)**						
≥0, < 1, mg/day	1090 (91.6)	3386 (92.0)	575 (92.3)	1926 (93.9)	515 (90.8)	1460 (89.7)
≥1, < 5, mg/day	43 (3.6)	161 (4.4)	19 (3.0)	73 (3.6)	24 (4.2)	88 (5.4)
≥5, < 7.5, mg/day	20 (1.7)	64 (1.7)	11 (1.8)	22 (1.1)	9 (1.6)	42 (2.6)
≥7.5, mg/day	37 (3.1)	69 (1.9)	18 (2.9)	31 (1.5)	19 (3.4)	38 (2.3)
**Drug classes for pre-perioperative osteoporosis medications[d], n (%)**						
No	831 (69.8)	1324 (36.0)	476 (76.4)	872 (42.5)	355 (62.6)	452 (27.8)
Anabolic agents	81 (6.8)	313 (8.5)	25 (4.0)	103 (5.0)	56 (9.9)	210 (12.9)
Parathyroid hormone	70 (5.9)	286 (7.8)	24 (3.9)	97 (4.7)	46 (8.1)	189 (11.6)
Romosozumab	11 (0.9)	27 (0.7)	1 (0.2)	6 (0.3)	10 (1.8)	21 (1.3)
Antiresorptive agents	174 (14.6)	1552 (42.2)	76 (12.2)	787 (38.4)	98 (17.3)	765 (47.0)
Bisphosphonate	146 (12.3)	1097 (29.8)	67 (10.8)	572 (27.9)	79 (13.9)	525 (32.2)
Selective estrogen receptor modulator	0 (0.0)	263 (7.1)	0 (0.0)	143 (7.0)	0 (0.0)	120 (7.4)

Anti-RANKL	28 (2.4)	192 (5.2)	9 (1.4)	72 (3.5)	19 (3.4)	120 (7.4)
Combination of two or more[e]	1 (0.1)	14 (0.4)	0 (0.0)	8 (0.4)	1 (0.2)	6 (0.4)
Bone metabolism-regulating drugs only[f]	103 (8.7)	477 (13.0)	46 (7.4)	282 (13.7)	57 (10.1)	195 (12.0)
**Pre-preoperative osteoporosis medications status, n (%)**						
Untreated[g]	934 (78.5)	1801 (48.9)	522 (83.8)	1154 (56.2)	412 (72.7)	647 (39.7)
Previously treated	256 (21.5)	1879 (51.1)	101 (16.2)	898 (43.8)	155 (27.3)	981 (60.3)
**History of fractures other than VF, n (%)**						
No	1056 (88.7)	3049 (82.9)	559 (89.7)	1737 (84.6)	497 (87.7)	1312 (80.6)
All hip fracture	16 (1.3)	97 (2.6)	10 (1.6)	52 (2.5)	6 (1.1)	45 (2.8)
NHNV fracture	114 (9.6)	485 (13.2)	51 (8.2)	238 (11.6)	63 (11.1)	247 (15.2)
Both[h]	4 (0.3)	49 (1.3)	3 (0.5)	25 (1.2)	1 (0.2)	24 (1.5)

LSEHS, latter-stage elderly healthcare system; NHI, national health insurance; NHNV, Non-hip non-vertebral; PVP, percutaneous vertebroplasty; RANKL, receptor activator of nuclear factor kappa-B ligand; SD, standard deviation; SFS, spine fusion surgery; SHI, society-managed employment-based health insurance association; VF, vertebral fracture

[a] Patients with chronic kidney disease, excluding dialysis

[b] Patients with mild liver disease, excluding moderate or severe liver disease

[c] As prednisolone equivalent mg/day. The mean dose per day calculated from the total dose per year in patients who were prescribed oral glucocorticoids for at least 90 days during the baseline period

[d] “Anabolic agents” and “antiresorptive agents” also include patients who use those agents in combination with “bone metabolism-regulating drugs”

[e] Excluding bone metabolism-regulating drugs

[f] A general term that includes active vitamin D, vitamin K, and calcium

[g] Untreated or bone metabolism-regulating drugs only

[h] Patients with a history of both all hip fracture together with NHNV during the baseline period

Supplemental Table 7 Subgroup analysis of perioperative treatment status by sex

	PVP group		SFS group	
	Male<br>N = 623	Female<br>N = 2052	Male<br>N = 567	Female<br>N = 1628
**Index year[a], n (%)**				
–2018	148 (23.8)	524 (25.5)	145 (25.6)	453 (27.8)
2019–	475 (76.2)	1528 (74.5)	422 (74.4)	1175 (72.2)
**No. of beds at medical institutions performing surgery, n (%)**				
≤ 19	15 (2.4)	77 (3.8)	10 (1.8)	20 (1.2)
20–199	273 (43.8)	886 (43.2)	87 (15.3)	281 (17.3)
200–399	147 (23.6)	504 (24.6)	182 (32.1)	502 (30.8)
≥ 400	188 (30.2)	585 (28.5)	288 (50.8)	825 (50.7)
missing	0 (0.0)	0 (0.0)	0 (0.0)	0 (0.0)
**Types of VF[b], n (%)**				
Thoracic vertebrae fracture	221 (35.5)	837 (40.8)	180 (31.7)	491 (30.2)
Lumbar fracture	470 (75.4)	1438 (70.1)	408 (72.0)	1127 (69.2)
Spinal fracture, unspecified	139 (22.3)	497 (24.2)	129 (22.8)	379 (23.3)
**Details of the surgical procedure[c], n (%)**				
Posterior spinal fusion	-	-	393 (69.3)	1142 (70.1)
Anterior spinal fusion[d]	-	-	143 (25.2)	429 (26.4)
Combined PVP	-	-	31 (5.5)	57 (3.5)
**Length of fusion area**				
1 level	-	-	16 (2.8)	85 (5.2)
2 levels	-	-	77 (13.6)	351 (21.6)
≥ 3 levels	-	-	474 (83.6)	1192 (73.2)
**Drug classes for perioperative osteoporosis medications[e], n (%)**				
No	198 (31.8)	331 (16.1)	171 (30.2)	245 (15.0)
Anabolic agents	212 (34.0)	758 (36.9)	253 (44.6)	748 (45.9)
Parathyroid hormone	177 (28.4)	627 (30.6)	218 (38.4)	678 (41.6)
Romosozumab	35 (5.6)	131 (6.4)	35 (6.2)	70 (4.3)
Antiresorptive agents	122 (19.6)	682 (33.2)	91 (16.0)	456 (28.0)
BP	95 (15.2)	450 (21.9)	74 (13.1)	315 (19.3)
SERM	0 (0.0)	127 (6.2)	0 (0.0)	75 (4.6)

Anti-RANKL	27 (4.3)	105 (5.1)	17 (3.0)	66 (4.1)
Combination of two or more[f]	0 (0.0)	8 (0.4)	0 (0.0)	4 (0.2)
Bone metabolism-regulating drugs only[g]	91 (14.6)	273 (13.3)	52 (9.2)	175 (10.7)
**Prescription start date relative to first surgery date[h], n (%)**	N = 163	N = 319	N = 163	N = 219
≥ −90, ≤ −61, days	8 (4.9)	11 (3.4)	23 (14.1)	16 (7.3)
≥ −60, ≤ −31, days	14 (8.6)	26 (8.2)	16 (9.8)	30 (13.7)
≥ −30, ≤ −1, days	98 (60.1)	185 (58.0)	83 (50.9)	125 (57.1)
≥ 1, ≤ 22, days	43 (26.4)	97 (30.4)	41 (25.2)	48 (21.9)

BP, bisphosphonate; PVP, percutaneous vertebroplasty; RANKL, receptor activator of nuclear factor kappa-B ligand; SERM, selective estrogen receptor modulator; SFS, spine fusion surgery; VF, vertebral fracture

[a] Year of initial spine surgery

[b] If multiple fractures occurred in the 12 months prior to the month of the initial spine surgery, the fracture closest to the initial spine surgery is counted. If multiple fractures occurred in the same month, duplicate counting is used

[c] For patients in the SFS group, spine surgeries claimed from the date of initial spine surgery to the day before the index date are counted. The details are as follows:

- Posterior spinal fusion: If this is the only procedure claimed during the evaluation period
- Anterior spinal fusion (including combined antero-posterior approaches): If no PVP was claimed during the evaluation period, but anterior spinal fusion or spinal osteotomy was claimed
- Combined PVP: If the evaluation period included a claim for PVP

[d] Including combined antero-posterior approaches

[e] “Anabolic agents” and “antiresorptive agents” also include patients who use those agents in combination with “bone metabolism-regulating drugs”

[f] Excluding bone metabolism-regulating drugs

[g] A general term that includes active vitamin D, vitamin K, and calcium

[h] The denominator was the number of patients who were newly prescribed anabolic agents in the perioperative period. Day 1 is the day of the initial spine surgery

Supplemental Table 8 Details of factors associated with reoperation in the PVP group

Variable	N	Cumulative incidence % at Day 365 (95% CI)	Unadjusted HR (95% CI)	Adjusted HR (95% CI)
Sex				
Male	623	7.89 (5.91, 10.50)	1.00	1.00
Female	2052	8.06 (6.88, 9.43)	1.07 (0.78, 1.48)	1.01 (0.72, 1.42)
Age categories				
40–69 years	178	7.11 (4.10, 12.20)	1.00	1.00
70–79 years	965	8.92 (7.18, 11.06)	1.35 (0.78, 2.33)	1.35 (0.78, 2.33)
≥ 80 years	1532	7.51 (6.21, 9.08)	1.12 (0.65, 1.93)	1.09 (0.63, 1.88)
Type of insurance				
SHI	8	0.00 (0.00, 0.00)	-	-
NHI	528	6.87 (4.92, 9.56)	-	-
LSEHS	2139	8.35 (7.17, 9.71)	-	-
Degenerative spine diseases				
No	1530	6.55 (5.34, 8.02)	1.00	1.00
Yes	1145	9.98 (8.27, 12.02)	1.43 (1.10, 1.85)	1.34 (1.03, 1.76)
Hip or knee osteoarthritis				
No	2466	7.99 (6.91, 9.23)	1.00	-
Yes	209	8.37 (5.19, 13.36)	1.17 (0.73, 1.87)	-
Hyperparathyroidism				
No	2631	7.96 (6.92, 9.16)	1.00	-
Yes	44	11.60 (4.48, 28.23)	1.13 (0.42, 3.04)	-
Hypoparathyroidism				
No	2673	8.02 (6.99, 9.21)	-	-
Yes	2	NE	-	-
Hyperthyroidism				
No	2616	7.97 (6.92, 9.16)	1.00	-
Yes	59	10.46 (4.83, 21.82)	1.46 (0.69, 3.09)	-
Hypothyroidism				
No	2466	7.95 (6.87, 9.18)	1.00	-
Yes	209	8.82 (5.54, 13.91)	1.10 (0.68, 1.79)	-
Chronic kidney disease				

No	2137	8.44 (7.25, 9.81)	1.00	-
Non-dialysis[a]	502	6.25 (4.38, 8.90)	0.83 (0.58, 1.19)	-
Dialysis	36	7.65 (1.95, 27.47)	0.64 (0.16, 2.58)	-
Diabetes mellitus				
No	1554	8.10 (6.74, 9.72)	1.00	-
Yes	1121	7.93 (6.41, 9.78)	1.16 (0.89, 1.51)	-
Parkinson's disease				
No	2518	7.79 (6.74, 9.00)	1.00	-
Yes	157	11.64 (7.26, 18.39)	1.24 (0.75, 2.06)	-
Hypertension				
No	718	8.24 (6.30, 10.74)	1.00	-
Yes	1957	7.91 (6.73, 9.30)	1.11 (0.82, 1.50)	-
Dyslipidemia				
No	1154	7.72 (6.21, 9.59)	1.00	-
Yes	1521	8.21 (6.87, 9.81)	1.12 (0.85, 1.46)	-
Myocardial infarction				
No	2585	8.08 (7.03, 9.30)	1.00	-
Yes	90	6.09 (2.57, 14.07)	0.98 (0.46, 2.09)	-
Congestive heart failure				
No	1821	7.81 (6.58, 9.27)	1.00	-
Yes	854	8.44 (6.68, 10.64)	1.18 (0.89, 1.55)	-
Cerebrovascular disease				
No	1840	8.02 (6.79, 9.47)	1.00	-
Yes	835	8.01 (6.24, 10.25)	1.05 (0.79, 1.40)	-
Dementia				
No	2309	7.83 (6.73, 9.10)	1.00	-
Yes	366	9.19 (6.49, 12.94)	1.09 (0.75, 1.59)	-
Chronic pulmonary disease				
No	1870	7.64 (6.44, 9.04)	1.00	-
Yes	805	8.92 (7.01, 11.30)	1.18 (0.89, 1.56)	-
Liver disease				
No	2024	8.11 (6.92, 9.49)	1.00	-
Mild[b]	622	7.50 (5.57, 10.06)	0.98 (0.71, 1.34)	-

Moderate or severe	29	13.14 (4.34, 36.10)	1.92 (0.71, 5.18)	-
Rheumatologic disease				
No	2441	7.88 (6.81, 9.11)	1.00	-
Yes	234	9.53 (6.13, 14.66)	1.37 (0.91, 2.06)	-
Malignant tumor				
No	2237	8.14 (7.00, 9.45)	1.00	-
Yes	438	7.34 (5.14, 10.43)	0.98 (0.68, 1.42)	-
Charlson Comorbidity Index				
0–2	1542	7.99 (6.64, 9.59)	1.00	-
≥ 3	1133	8.04 (6.51, 9.91)	1.11 (0.85, 1.45)	-
Psychotropic drugs				
No	1408	6.25 (5.02, 7.76)	1.00	1.00
Yes	1267	9.97 (8.33, 11.90)	1.39 (1.06, 1.81)	1.34 (1.03, 1.76)
Narcotics				
No	2645	7.93 (6.89, 9.11)	1.00	-
Yes	30	15.56 (6.05, 36.75)	1.54 (0.57, 4.15)	-
Anticonvulsants				
No	2063	6.98 (5.89, 8.26)	1.00	-
Yes	612	11.41 (8.99, 14.44)	1.51 (1.14, 2.01)	-
Muscle relaxants				
No	2350	7.74 (6.65, 8.99)	1.00	-
Yes	325	10.01 (7.04, 14.13)	1.19 (0.82, 1.72)	-
Proton pump inhibitor				
No	1275	6.90 (5.54, 8.58)	1.00	-
Yes	1400	9.03 (7.55, 10.78)	1.39 (1.06, 1.81)	-
Anticoagulants (warfarin and heparin)				
No	2521	8.08 (7.01, 9.31)	1.00	-
Yes	154	6.89 (3.76, 12.44)	0.95 (0.53, 1.71)	-
Hormone replacement therapy (estrogen only)				
No	2657	7.99 (6.95, 9.18)	1.00	-
Yes	18	12.30 (3.19, 41.20)	1.34 (0.33, 5.39)	-
Endocrine therapy except estrogen for breast cancer and prostate cancer				
No	2613	8.17 (7.11, 9.38)	1.00	-

Yes	62	1.72 (0.24, 11.62)	0.57 (0.18, 1.78)	-
Glucocorticoids mean dose[c]				
≥ 0, < 1, mg/day	2501	7.83 (6.77, 9.05)	1.00	1.00
≥ 1, < 5, mg/day	92	8.07 (3.92, 16.26)	1.42 (0.75, 2.68)	1.25 (0.66, 2.37)
≥ 5, < 7.5, mg/day	33	19.83 (9.29, 39.42)	2.46 (1.09, 5.55)	2.35 (1.04, 5.34)
≥ 7.5, mg/day	49	9.73 (3.64, 24.65)	1.28 (0.53, 3.10)	1.08 (0.44, 2.67)
History of fractures other than VF				
No	2296	7.91 (6.80, 9.19)	1.00	1.00
All hip fracture	62	15.63 (8.43, 27.96)	1.98 (1.01, 3.86)	1.83 (0.93, 3.59)
NHNV fracture	289	7.78 (5.07, 11.83)	1.07 (0.70, 1.62)	0.96 (0.63, 1.47)
Both[d]	28	3.57 (0.51, 22.76)	0.43 (0.06, 3.04)	0.43 (0.06, 3.05)
Index year				
–2018	672	8.98 (7.01, 11.47)	1.00	-
2019–	2003	7.53 (6.37, 8.90)	0.92 (0.69, 1.24)	-
No. of beds at medical institutions performing surgery				
≤ 19	92	5.75 (2.43, 13.30)	0.66 (0.29, 1.50)	-
20–199	1159	8.29 (6.73, 10.20)	1.00	-
200–399	651	7.10 (5.28, 9.51)	0.93 (0.66, 1.31)	-
≥ 400	773	8.60 (6.71, 10.99)	0.98 (0.72, 1.33)	-
Missing	0	-	-	-
Pre-preoperative osteoporosis medications status				
Untreated[e]	1676	6.85 (5.67, 8.25)	1.00	1.00
Previously treated	999	9.94 (8.10, 12.17)	1.33 (1.02, 1.73)	1.20 (0.88, 1.63)
Drug classes for perioperative osteoporosis medications[f]				
No	529	9.22 (6.89, 12.28)	1.00	1.00
Anabolic agents[g]	970	7.27 (5.71, 9.23)	0.77 (0.54, 1.12)	0.69 (0.47, 1.01)
Antiresorptive agents	804	9.05 (7.15, 11.43)	1.07 (0.75, 1.54)	0.92 (0.62, 1.38)
Combination of two or more[h]	8	12.50 (1.86, 61.30)	2.66 (0.65, 10.94)	2.44 (0.57, 10.41)
Bone metabolism-regulating drugs only[i]	364	5.97 (3.76, 9.41)	0.67 (0.40, 1.10)	0.65 (0.39, 1.09)

CI, confidence interval; HR, hazard ratio; LSEHS, latter-stage elderly healthcare system; NE, not estimable; NHI, national health insurance; NHNV, non-hip non-vertebral; PVP, percutaneous vertebroplasty; SHI, society-managed employment-based health insurance association; VF, vertebral fracture

[a] Patients with chronic kidney disease, excluding dialysis

[b] Patients with mild liver disease, excluding moderate or severe liver disease

[c] As prednisolone equivalent mg/day. The mean dose per day calculated from the total dose per year in patients who were prescribed oral glucocorticoids for at least 90 days during the baseline period

[d] Patients with a history of both all hip fracture together with NHNV during the baseline period

[e] Untreated or bone metabolism-regulating drugs only
[f] "Anabolic agents" and "antiresorptive agents" also include patients who use those agents in combination with "bone metabolism-regulating drugs"
[g] Anabolic agents: PTH and Rmab
[h] Excluding bone metabolism-regulating drugs
[i] A general term that includes active vitamin D, vitamin K, and calcium

Supplemental Table 9 Details of factors associated with reoperation in the SFS group

Variable	N	Cumulative incidence % at Day 365 (95% CI)	Unadjusted HR (95% CI)	Adjusted HR (95% CI)
Sex				
Male	567	6.67 (4.76, 9.31)	1.00	1.00
Female	1628	5.69 (4.60, 7.03)	0.91 (0.63, 1.32)	0.94 (0.64, 1.38)
Age categories				
40–69 years	247	5.68 (3.33, 9.60)	1.00	1.00
70–79 years	984	5.89 (4.51, 7.67)	0.82 (0.51, 1.31)	0.84 (0.52, 1.36)
≥ 80 years	964	6.06 (4.61, 7.96)	0.71 (0.44, 1.17)	0.73 (0.44, 1.22)
Type of insurance				
SHI	23	8.70 (2.25, 30.51)	0.97 (0.23, 3.99)	-
NHI	541	5.26 (3.60, 7.66)	1.00	-
LSEHS	1631	6.13 (4.98, 7.53)	0.83 (0.58, 1.20)	-
Degenerative spine diseases				
No	635	6.75 (4.95, 9.19)	1.00	-
Yes	1560	5.61 (4.50, 6.99)	0.82 (0.58, 1.16)	-
Hip or knee osteoarthritis				
No	1939	6.13 (5.08, 7.38)	1.00	-
Yes	256	4.55 (2.46, 8.35)	0.76 (0.43, 1.35)	-
Hyperparathyroidism				
No	2137	5.89 (4.90, 7.06)	1.00	1.00
Yes	58	7.92 (3.01, 20.00)	2.25 (1.10, 4.59)	2.14 (1.03, 4.44)
Hypoparathyroidism				
No	2194	5.95 (4.97, 7.11)	-	-
Yes	1	0.00 (0.00, 0.00)	-	-
Hyperthyroidism				
No	2137	6.06 (5.06, 7.25)	1.00	-
Yes	58	1.72 (0.24, 11.62)	0.77 (0.25, 2.41)	-
Hypothyroidism				
No	1993	5.96 (4.94, 7.19)	1.00	-
Yes	202	5.79 (3.13, 10.60)	1.15 (0.66, 1.99)	-
Chronic kidney disease				

No	1700	5.83 (4.74, 7.16)	1.00	-
Non-dialysis[a]	456	6.19 (4.21, 9.06)	0.95 (0.63, 1.43)	-
Dialysis	39	7.98 (2.64, 22.75)	1.21 (0.38, 3.80)	-
Diabetes mellitus				
No	1279	6.54 (5.22, 8.18)	1.00	-
Yes	916	5.13 (3.80, 6.89)	0.85 (0.61, 1.19)	-
Parkinson's disease				
No	2050	5.44 (4.47, 6.62)	1.00	1.00
Yes	145	12.94 (8.35, 19.79)	2.52 (1.59, 4.01)	2.10 (1.31, 3.37)
Hypertension				
No	538	5.69 (3.92, 8.22)	1.00	-
Yes	1657	6.01 (4.90, 7.38)	0.86 (0.60, 1.24)	-
Dyslipidemia				
No	904	6.51 (4.98, 8.48)	1.00	-
Yes	1291	5.55 (4.35, 7.06)	0.82 (0.59, 1.13)	-
Myocardial infarction				
No	2120	6.00 (5.00, 7.19)	1.00	-
Yes	75	4.50 (1.45, 13.52)	0.82 (0.30, 2.22)	-
Congestive heart failure				
No	1462	5.64 (4.50, 7.06)	1.00	-
Yes	733	6.55 (4.86, 8.79)	1.17 (0.83, 1.65)	-
Cerebrovascular disease				
No	1559	5.88 (4.75, 7.28)	1.00	-
Yes	636	6.10 (4.38, 8.48)	0.99 (0.69, 1.42)	-
Dementia				
No	1981	6.00 (4.97, 7.23)	1.00	-
Yes	214	5.42 (2.92, 9.95)	0.84 (0.45, 1.55)	-
Chronic pulmonary disease				
No	1557	6.31 (5.12, 7.75)	1.00	-
Yes	638	5.06 (3.53, 7.22)	0.91 (0.63, 1.32)	-
Liver disease				
No	1596	6.22 (5.07, 7.63)	-	-
Mild[b]	584	5.30 (3.65, 7.66)	-	-

Moderate or severe	15	0.00 (0.00, 0.00)	-	-
Rheumatologic disease				
No	1918	5.78 (4.75, 7.03)	1.00	-
Yes	277	7.05 (4.48, 10.99)	1.31 (0.84, 2.05)	-
Malignant tumor				
No	1884	5.59 (4.57, 6.81)	1.00	-
Yes	311	8.13 (5.38, 12.19)	1.33 (0.85, 2.08)	-
Charlson Comorbidity Index				
0–2	1243	5.23 (4.05, 6.74)	1.00	-
≥ 3	952	6.88 (5.35, 8.84)	1.27 (0.92, 1.77)	-
Psychotropic drugs				
No	885	6.08 (4.58, 8.03)	1.00	-
Yes	1310	5.85 (4.63, 7.38)	0.95 (0.68, 1.33)	-
Narcotics				
No	2174	6.00 (5.02, 7.18)	1.00	-
Yes	21	0.00 (0.00, 0.00)	0.63 (0.09, 4.50)	-
Anticonvulsants				
No	1139	5.54 (4.27, 7.18)	1.00	-
Yes	1056	6.35 (4.95, 8.12)	1.16 (0.84, 1.62)	-
Muscle relaxants				
No	1901	5.92 (4.87, 7.18)	1.00	-
Yes	294	6.11 (3.78, 9.81)	1.05 (0.66, 1.67)	-
Proton pump inhibitor				
No	856	6.20 (4.66, 8.23)	1.00	-
Yes	1339	5.78 (4.58, 7.27)	0.96 (0.68, 1.34)	-
Anticoagulants (warfarin and heparin)				
No	2022	5.98 (4.96, 7.21)	1.00	-
Yes	173	5.60 (2.93, 10.56)	1.04 (0.57, 1.87)	-
Hormone replacement therapy (estrogen-only)				
No	2183	5.98 (5.00, 7.15)	1.00	-
Yes	12	0.00 (0.00, 0.00)	1.02 (0.14, 7.31)	-
Endocrine therapy except estrogen for breast cancer and prostate cancer				
No	2152	5.91 (4.92, 7.08)	1.00	-

Yes	43	7.30 (2.41, 21.02)	1.28 (0.41, 4.01)	-
Glucocorticoids mean dose[c]				
≥0, < 1, mg/day	1975	5.86 (4.84, 7.08)	1.00	-
≥1, < 5, mg/day	112	8.21 (4.17, 15.84)	1.26 (0.64, 2.48)	-
≥5, < 7.5, mg/day	51	7.02 (2.24, 20.83)	1.38 (0.51, 3.73)	-
≥ 7.5, mg/day	57	3.51 (0.89, 13.31)	1.54 (0.68, 3.49)	-
History of fractures other than VF				
No	1809	6.02 (4.95, 7.32)	1.00	-
All hip fracture	51	1.96 (0.28, 13.11)	0.56 (0.14, 2.26)	-
NHNV fracture	310	6.35 (3.98, 10.06)	1.05 (0.66, 1.67)	-
Both[d]	25	4.00 (0.57, 25.16)	0.65 (0.09, 4.64)	-
Index year				
–2018	598	5.03 (3.52, 7.16)	1.00	-
2019–	1597	6.30 (5.11, 7.75)	1.28 (0.88, 1.86)	-
No. of beds at medical institutions performing surgery				
≤ 19	30	3.57 (0.51, 22.76)	0.72 (0.09, 5.41)	0.71 (0.09, 5.49)
20–199	368	2.76 (1.43, 5.29)	1.00	1.00
200–399	684	6.68 (4.90, 9.06)	1.94 (1.09, 3.45)	1.54 (0.86, 2.77)
≥ 400	1113	6.61 (5.22, 8.36)	1.71 (0.98, 2.97)	1.31 (0.74, 2.31)
missing	0	-	-	-
Details of the surgical procedure				
Posterior spinal fusion	1535	5.74 (4.62, 7.13)	1.00	1.00
Anterior spinal fusion[e]	572	6.73 (4.83, 9.33)	1.27 (0.89, 1.82)	1.14 (0.79, 1.65)
Combined PVP	88	4.09 (1.32, 12.25)	1.21 (0.53, 2.77)	1.17 (0.51, 2.70)
Length of fusion area				
1 level	101	1.35 (0.19, 9.21)	0.12 (0.02, 0.87)	0.17 (0.02, 1.26)
2 levels	428	4.46 (2.79, 7.11)	0.69 (0.43, 1.08)	0.76 (0.48, 1.21)
≥ 3 levels	1666	6.62 (5.44, 8.03)	1.00	1.00
Pre-preoperative osteoporosis medications status				
Untreated[f]	1059	5.51 (4.22, 7.19)	1.00	-
Previously treated	1136	6.34 (4.97, 8.07)	1.19 (0.86, 1.66)	-
Drug classes for perioperative osteoporosis medications[g]				
No	416	4.44 (2.77, 7.06)	1.00	1.00

Anabolic agents[h]	1001	6.87 (5.37, 8.77)	1.58 (0.97, 2.59)	1.54 (0.94, 2.55)
Antiresorptive agents	547	6.45 (4.53, 9.14)	1.45 (0.84, 2.49)	1.55 (0.89, 2.69)
Combination of two or more[i]	4	25.00 (3.95, 87.21)	5.40 (0.72, 40.22)	4.23 (0.55, 32.68)
Bone metabolism-regulating drugs only[j]	227	3.07 (1.38, 6.78)	0.94 (0.44, 2.01)	0.94 (0.44, 2.01)

CI, confidence interval; HR, hazard ratio; LSEHS, latter-stage elderly healthcare system; NHI, national health insurance; NHNV, non-hip non-vertebral; PVP, percutaneous vertebroplasty; SFS, spine fusion surgery; SHI, society-managed employment-based health insurance association; VF, vertebral fracture

[a] Patients with chronic kidney disease, excluding dialysis

[b] Patients with mild liver disease, excluding moderate or severe liver disease

[c] As prednisolone equivalent mg/day. The mean dose per day calculated from the total dose per year in patients who were prescribed oral glucocorticoids for at least 90 days during the baseline period

[d] Patients with a history of both all hip fracture together with NHNV during the baseline period

[e] Including combined antero-posterior approaches

[f] Untreated or bone metabolism-regulating drugs only

[g] "Anabolic agents" and "antiresorptive agents" also include patients who use those agents in combination with "bone metabolism-regulating drugs"

[h] Anabolic agents: PTH and Rmab

[i] Excluding bone metabolism-regulating drugs

[j] A general term that includes active vitamin D, vitamin K, and calcium

Supplemental Table 10 Factors associated with subsequent VF in the PVP group

Variable	N	Cumulative incidence % at Day 365 (95% CI)	Unadjusted HR (95% CI)	Adjusted HR (95% CI)
Sex				
Male	623	7.20 (5.31, 9.73)	1.00	1.00
Female	2052	7.94 (6.77, 9.30)	1.23 (0.88, 1.72)	1.15 (0.82, 1.63)
Age categories				
40–69 years	178	7.09 (4.09, 12.17)	1.00	1.00
70–79 years	965	8.92 (7.16, 11.07)	1.35 (0.78, 2.33)	1.33 (0.77, 2.31)
≥ 80 years	1532	7.07 (5.82, 8.58)	1.13 (0.66, 1.94)	1.12 (0.65, 1.93)
Type of insurance				
SHI	8	0.00 (0.00, 0.00)	-	-
NHI	528	7.43 (5.40, 10.18)	-	-
LSEHS	2139	7.89 (6.74, 9.22)	-	-
Degenerative spine diseases				
No	1530	6.55 (5.35, 8.02)	1.00	-
Yes	1145	9.39 (7.72, 11.39)	1.29 (0.99, 1.68)	-
Hip or knee osteoarthritis				
No	2466	7.66 (6.60, 8.87)	1.00	-
Yes	209	9.06 (5.70, 14.26)	1.18 (0.74, 1.89)	-
Hyperparathyroidism				
No	2631	7.71 (6.69, 8.88)	1.00	-
Yes	44	11.26 (4.32, 27.60)	1.45 (0.60, 3.52)	-
Hypoparathyroidism				
No	2673	7.77 (6.75, 8.94)	-	-
Yes	2	NE	-	-
Hyperthyroidism				
No	2616	7.71 (6.68, 8.89)	1.00	-
Yes	59	10.46 (4.83, 21.82)	1.45 (0.68, 3.09)	-
Hypothyroidism				
No	2466	7.51 (6.47, 8.71)	1.00	-
Yes	209	10.91 (7.11, 16.55)	1.25 (0.79, 1.99)	-
Chronic kidney disease				

No	2137	7.99 (6.84, 9.32)	1.00	-
Non-dialysis[a]	502	6.61 (4.65, 9.36)	0.90 (0.63, 1.27)	-
Dialysis	36	10.58 (3.47, 29.83)	1.02 (0.33, 3.19)	-
Diabetes mellitus				
No	1554	8.13 (6.77, 9.75)	1.00	-
Yes	1121	7.28 (5.84, 9.06)	1.08 (0.83, 1.41)	-
Parkinson's disease				
No	2518	7.42 (6.40, 8.60)	1.00	-
Yes	157	13.22 (8.49, 20.27)	1.52 (0.95, 2.44)	-
Hypertension				
No	718	7.32 (5.51, 9.69)	1.00	-
Yes	1957	7.92 (6.73, 9.30)	1.29 (0.94, 1.76)	-
Dyslipidemia				
No	1154	7.46 (5.98, 9.29)	1.00	-
Yes	1521	7.97 (6.65, 9.55)	1.17 (0.90, 1.54)	-
Myocardial infarction				
No	2585	7.91 (6.86, 9.11)	1.00	-
Yes	90	3.53 (1.15, 10.55)	0.97 (0.46, 2.06)	-
Congestive heart failure				
No	1821	7.52 (6.32, 8.95)	1.00	-
Yes	854	8.28 (6.52, 10.48)	1.12 (0.84, 1.48)	-
Cerebrovascular disease				
No	1840	7.39 (6.21, 8.78)	1.00	-
Yes	835	8.62 (6.77, 10.94)	1.16 (0.88, 1.53)	-
Dementia				
No	2309	7.26 (6.21, 8.49)	1.00	-
Yes	366	10.98 (7.96, 15.04)	1.28 (0.90, 1.83)	-
Chronic pulmonary disease				
No	1870	7.44 (6.27, 8.82)	1.00	-
Yes	805	8.54 (6.67, 10.90)	1.16 (0.88, 1.54)	-
Liver disease				
No	2024	7.68 (6.52, 9.02)	1.00	-
Mild[b]	622	8.07 (6.05, 10.71)	1.01 (0.74, 1.39)	-

Moderate or severe	29	7.72 (1.98, 27.52)	1.44 (0.46, 4.51)	-
Rheumatologic disease				
No	2441	7.68 (6.63, 8.89)	1.00	-
Yes	234	8.75 (5.46, 13.84)	1.37 (0.91, 2.07)	-
Malignant tumor				
No	2237	7.86 (6.75, 9.14)	1.00	-
Yes	438	7.27 (5.05, 10.42)	0.92 (0.63, 1.34)	-
Charlson Comorbidity Index				
0–2	1542	7.58 (6.28, 9.13)	1.00	-
≥ 3	1133	8.02 (6.48, 9.90)	1.06 (0.81, 1.39)	-
Psychotropic drugs				
No	1408	6.11 (4.90, 7.60)	1.00	1.00
Yes	1267	9.58 (7.98, 11.48)	1.37 (1.05, 1.79)	1.37 (1.05, 1.79)
Narcotics				
No	2645	7.73 (6.71, 8.90)	1.00	-
Yes	30	11.11 (3.67, 31.02)	1.12 (0.36, 3.50)	-
Anticonvulsants				
No	2063	6.97 (5.88, 8.25)	1.00	-
Yes	612	10.38 (8.08, 13.29)	1.33 (0.99, 1.78)	-
Muscle relaxants				
No	2350	7.50 (6.44, 8.73)	1.00	-
Yes	325	9.64 (6.74, 13.70)	1.15 (0.78, 1.67)	-
Proton pump inhibitor				
No	1275	7.25 (5.86, 8.96)	1.00	-
Yes	1400	8.22 (6.82, 9.90)	1.18 (0.90, 1.54)	-
Anticoagulants (warfarin and heparin)				
No	2521	7.82 (6.76, 9.03)	1.00	-
Yes	154	6.86 (3.75, 12.41)	0.87 (0.48, 1.60)	-
Hormone replacement therapy (estrogen-only)				
No	2657	7.74 (6.72, 8.91)	1.00	-
Yes	18	12.30 (3.19, 41.20)	1.33 (0.33, 5.34)	-
Endocrine therapy except estrogen for breast cancer and prostate cancer				
No	2613	7.95 (6.91, 9.14)	1.00	-

Yes	62	0.00 (0.00, 0.00)	0.38 (0.09, 1.51)	-
Glucocorticoids mean dose[c]				
≥ 0, < 1, mg/day	2501	7.65 (6.61, 8.85)	1.00	-
≥ 1, < 5, mg/day	92	9.45 (4.81, 18.09)	1.72 (0.96, 3.09)	-
≥ 5, < 7.5, mg/day	33	12.23 (4.77, 29.38)	1.56 (0.58, 4.20)	-
≥ 7.5, mg/day	49	7.51 (2.38, 22.34)	1.01 (0.38, 2.72)	-
History of fractures other than VF				
No	2296	7.56 (6.47, 8.81)	1.00	-
All hip fracture	62	13.99 (7.24, 26.11)	1.77 (0.87, 3.58)	-
NHNV fracture	289	8.18 (5.38, 12.33)	1.19 (0.79, 1.77)	-
Both[d]	28	7.43 (1.91, 26.61)	0.90 (0.22, 3.63)	-
Index year				
–2018	672	9.29 (7.29, 11.81)	1.00	-
2019–	2003	7.03 (5.91, 8.34)	0.76 (0.57, 1.02)	-
No. of beds at medical institutions performing surgery				
≤ 19	92	14.20 (8.29, 23.76)	1.59 (0.91, 2.79)	-
20–199	1159	8.57 (6.98, 10.50)	1.00	-
200–399	651	6.12 (4.46, 8.36)	0.80 (0.56, 1.14)	-
≥ 400	773	7.07 (5.38, 9.27)	0.82 (0.60, 1.13)	-
Missing	0	-	-	-
Pre-preoperative osteoporosis medications status				
Untreated[e]	1676	6.92 (5.74, 8.34)	1.00	1.00
Previously treated	999	9.15 (7.40, 11.30)	1.27 (0.97, 1.65)	1.14 (0.84, 1.54)
Drug classes for perioperative osteoporosis medications[f]				
No	529	8.99 (6.69, 12.03)	1.00	1.00
Anabolic agents[g]	970	7.23 (5.68, 9.18)	0.85 (0.58, 1.22)	0.78 (0.53, 1.14)
Antiresorptive agents	804	8.59 (6.74, 10.91)	1.11 (0.77, 1.60)	0.99 (0.66, 1.48)
Combination of two or more[h]	8	12.50 (1.86, 61.30)	2.77 (0.67, 11.42)	2.61 (0.61, 11.07)
Bone metabolism-regulating drugs only[i]	364	5.60 (3.48, 8.95)	0.66 (0.40, 1.12)	0.64 (0.38, 1.08)

CI, confidence interval; HR, hazard ratio; LSEHS, latter-stage elderly health care system; NE, not estimable; NHI, national health insurance; NHNV, non-hip non-vertebral; PVP, percutaneous vertebroplasty; SHI, society-managed employment-based health insurance association; VF, vertebral fracture

[a] Patients with chronic kidney disease, excluding dialysis

[b] Patients with mild liver disease, excluding moderate or severe liver disease

[c] As prednisolone equivalent mg/day. The mean dose per day calculated from the total dose per year in patients who were prescribed oral glucocorticoids for at least 90 days during the baseline period

[d] Patients with a history of both all hip fracture together with NHNV during the baseline period

[e] Untreated or bone metabolism-regulating drugs only
[f] “Anabolic agents” and “antiresorptive agents” also include patients who use those agents in combination with “bone metabolism-regulating drugs”
[g] Anabolic agents: PTH and Rmab
[h] Excluding bone metabolism-regulating drugs
[i] A general term that includes active vitamin D, vitamin K, and calcium

Supplemental Table 11 Factors associated with subsequent VF in the SFS group

Variable	N	Cumulative incidence % at Day 365 (95% CI)	Unadjusted HR (95% CI)	Adjusted HR (95% CI)
Sex				
Male	567	7.49 (5.48, 10.20)	1.00	1.00
Female	1628	5.85 (4.74, 7.20)	0.74 (0.52, 1.04)	0.80 (0.56, 1.14)
Age categories				
40–69 years	247	4.53 (2.54, 8.04)	1.00	1.00
70–79 years	984	6.17 (4.76, 7.98)	1.17 (0.70, 1.96)	1.18 (0.70, 1.99)
≥ 80 years	964	6.94 (5.37, 8.95)	1.06 (0.63, 1.80)	1.11 (0.65, 1.90)
Type of insurance				
SHI	23	0.00 (0.00, 0.00)	-	-
NHI	541	5.87 (4.14, 8.29)	-	-
LSEHS	1631	6.54 (5.35, 7.98)	-	-
Degenerative spine diseases				
No	635	7.01 (5.18, 9.47)	1.00	-
Yes	1560	5.97 (4.83, 7.36)	0.85 (0.61, 1.20)	-
Hip or knee osteoarthritis				
No	1939	6.62 (5.53, 7.91)	1.00	1.00
Yes	256	3.61 (1.80, 7.15)	0.47 (0.24, 0.92)	0.50 (0.26, 0.99)
Hyperparathyroidism				
No	2137	6.27 (5.26, 7.46)	1.00	-
Yes	58	6.17 (2.01, 18.16)	1.24 (0.51, 3.02)	-
Hypoparathyroidism				
No	2194	6.27 (5.27, 7.45)	-	-
Yes	1	0.00 (0.00, 0.00)	-	-
Hyperthyroidism				
No	2137	6.40 (5.37, 7.60)	1.00	-
Yes	58	1.72 (0.24, 11.62)	0.47 (0.12, 1.91)	-
Hypothyroidism				
No	1993	6.21 (5.18, 7.45)	1.00	-
Yes	202	6.87 (3.92, 11.88)	1.16 (0.68, 1.97)	-
Chronic kidney disease				

No	1700	6.14 (5.03, 7.49)	1.00	-
Non-dialysis[a]	456	6.82 (4.74, 9.76)	1.07 (0.72, 1.57)	-
Dialysis	39	5.41 (1.38, 19.93)	0.75 (0.18, 3.02)	-
Diabetes mellitus				
No	1279	6.80 (5.47, 8.46)	1.00	-
Yes	916	5.53 (4.16, 7.33)	0.86 (0.62, 1.19)	-
Parkinson's disease				
No	2050	5.79 (4.79, 6.98)	1.00	1.00
Yes	145	13.00 (8.38, 19.87)	2.18 (1.36, 3.50)	1.91 (1.18, 3.10)
Hypertension				
No	538	6.13 (4.31, 8.67)	1.00	-
Yes	1657	6.31 (5.17, 7.70)	0.91 (0.64, 1.30)	-
Dyslipidemia				
No	904	7.09 (5.51, 9.10)	1.00	-
Yes	1291	5.70 (4.48, 7.23)	0.82 (0.60, 1.13)	-
Myocardial infarction				
No	2120	6.40 (5.37, 7.61)	1.00	-
Yes	75	2.67 (0.67, 10.24)	0.56 (0.18, 1.77)	-
Congestive heart failure				
No	1462	6.01 (4.84, 7.45)	1.00	-
Yes	733	6.81 (5.09, 9.09)	1.12 (0.80, 1.56)	-
Cerebrovascular disease				
No	1559	6.22 (5.06, 7.63)	1.00	-
Yes	636	6.41 (4.64, 8.82)	0.96 (0.67, 1.37)	-
Dementia				
No	1981	6.44 (5.38, 7.70)	1.00	-
Yes	214	4.65 (2.43, 8.79)	0.69 (0.37, 1.32)	-
Chronic pulmonary disease				
No	1557	6.76 (5.54, 8.23)	1.00	-
Yes	638	5.08 (3.55, 7.24)	0.88 (0.62, 1.27)	-
Liver disease				
No	1596	6.61 (5.43, 8.05)	-	-
Mild[b]	584	5.46 (3.79, 7.84)	-	-

Moderate or severe	15	0.00 (0.00, 0.00)	-	-
Rheumatologic disease				
No	1918	6.06 (5.01, 7.32)	1.00	-
Yes	277	7.71 (5.03, 11.73)	1.15 (0.73, 1.81)	-
Malignant tumor				
No	1884	5.73 (4.71, 6.96)	1.00	1.00
Yes	311	9.76 (6.67, 14.16)	1.56 (1.04, 2.36)	1.50 (0.99, 2.28)
Charlson Comorbidity Index				
0–2	1243	5.79 (4.56, 7.34)	1.00	-
≥ 3	952	6.92 (5.38, 8.89)	1.21 (0.88, 1.66)	-
Psychotropic drugs				
No	885	5.86 (4.42, 7.74)	1.00	-
Yes	1310	6.55 (5.25, 8.15)	1.04 (0.75, 1.44)	-
Narcotics				
No	2174	6.33 (5.33, 7.53)	-	-
Yes	21	0.00 (0.00, 0.00)	-	-
Anticonvulsants				
No	1139	6.39 (5.03, 8.10)	1.00	-
Yes	1056	6.11 (4.75, 7.85)	0.88 (0.64, 1.21)	-
Muscle relaxants				
No	1901	6.19 (5.13, 7.46)	1.00	-
Yes	294	6.80 (4.33, 10.61)	1.20 (0.78, 1.85)	-
Proton pump inhibitor				
No	856	6.04 (4.55, 8.01)	1.00	-
Yes	1339	6.42 (5.15, 7.97)	1.11 (0.80, 1.54)	-
Anticoagulants (warfarin and heparin)				
No	2022	6.22 (5.19, 7.45)	1.00	-
Yes	173	6.97 (3.88, 12.34)	1.07 (0.60, 1.88)	-
Hormone replacement therapy (estrogen-only)				
No	2183	6.31 (5.30, 7.49)	-	-
Yes	12	0.00 (0.00, 0.00)	-	-
Endocrine therapy except estrogen for breast cancer and prostate cancer				
No	2152	6.24 (5.24, 7.43)	1.00	-

Yes	43	7.30 (2.41, 21.02)	1.58 (0.59, 4.28)	-
Glucocorticoids mean dose[c]				
≥ 0, < 1 mg/day	1975	6.27 (5.21, 7.52)	1.00	-
≥ 1, < 5 mg/day	112	7.86 (3.99, 15.14)	1.03 (0.50, 2.10)	-
≥ 5, < 7.5 mg/day	51	5.92 (1.95, 17.25)	1.24 (0.46, 3.35)	-
≥ 7.5 mg/day	57	3.51 (0.89, 13.31)	1.16 (0.48, 2.83)	-
History of fractures other than VF				
No	1809	6.47 (5.37, 7.80)	1.00	-
All hip fracture	51	1.96 (0.28, 13.11)	0.51 (0.13, 2.04)	-
NHNV fracture	310	6.05 (3.73, 9.75)	0.86 (0.53, 1.38)	-
Both[d]	25	4.00 (0.57, 25.16)	0.59 (0.08, 4.22)	-
Index year				
–2018	598	6.38 (4.66, 8.70)	1.00	-
2019–	1597	6.22 (5.04, 7.66)	0.95 (0.67, 1.34)	-
No. of beds at medical institutions performing surgery				
≤ 19	30	10.37 (3.46, 28.84)	2.50 (0.85, 7.40)	-
20–199	368	3.68 (2.09, 6.44)	1.00	-
200–399	684	6.37 (4.66, 8.68)	1.58 (0.92, 2.70)	-
≥ 400	1113	6.93 (5.51, 8.70)	1.51 (0.91, 2.52)	-
missing	0	-	-	-
Details of the surgical procedure				
Posterior spinal fusion	1535	5.58 (4.46, 6.96)	1.00	1.00
Anterior spinal fusion[e]	572	8.10 (6.06, 10.79)	1.72 (1.23, 2.41)	1.66 (1.18, 2.33)
Combined PVP	88	6.59 (2.77, 15.20)	1.72 (0.84, 3.55)	1.54 (0.74, 3.21)
Length of fusion area				
1 level	101	1.12 (0.16, 7.71)	0.23 (0.06, 0.93)	0.29 (0.07, 1.18)
2 levels	428	5.06 (3.24, 7.87)	0.74 (0.48, 1.14)	0.85 (0.55, 1.31)
≥ 3 levels	1666	6.91 (5.72, 8.34)	1.00	1.00
Pre-preoperative osteoporosis medications status				
Untreated[f]	1059	5.77 (4.45, 7.47)	1.00	-
Previously treated	1136	6.72 (5.32, 8.48)	1.14 (0.83, 1.58)	-
Drug classes for perioperative osteoporosis medications[g]				
No	416	5.86 (3.88, 8.79)	1.00	1.00

Anabolic agents[h]	1001	7.05 (5.55, 8.94)	1.15 (0.75, 1.78)	1.11 (0.72, 1.73)
Antiresorptive agents	547	6.55 (4.63, 9.23)	1.08 (0.67, 1.76)	1.18 (0.72, 1.94)
Combination of two or more[i]	4	25.00 (3.95, 87.21)	3.84 (0.52, 28.22)	3.70 (0.49, 28.07)
Bone metabolism-regulating drugs only[j]	227	2.54 (1.05, 6.10)	0.53 (0.24, 1.16)	0.54 (0.25, 1.20)

CI, confidence interval; HR, hazard ratio; LSEHS, latter-stage elderly healthcare system; NHI, national health insurance; NHNV, non-hip non-vertebral; PVP, percutaneous vertebroplasty; SFS, spine fusion surgery; SHI, society-managed employment-based health insurance association; VF, vertebral fracture

[a] Patients with chronic kidney disease, excluding dialysis

[b] Patients with mild liver disease, excluding moderate or severe liver disease

[c] As prednisolone equivalent mg/day. The mean dose per day calculated from the total dose per year in patients who were prescribed oral glucocorticoids for at least 90 days during the baseline period

[d] Patients with a history of both all hip fracture together with NHNV during the baseline period

[e] Including combined antero-posterior approaches

[f] Untreated or bone metabolism-regulating drugs only

[g] "Anabolic agents" and "antiresorptive agents" also include patients who use those agents in combination with "bone metabolism-regulating drugs"

[h] Anabolic agents: PTH and Rmab

[i] Excluding bone metabolism-regulating drugs

[j] A general term that includes active vitamin D, vitamin K, and calcium

Supplemental Table 12 Subgroup analysis of patient demographics (sex, age, insurance type), by year of first spine surgery

	All<br>Index year		PVP group<br>Index year		SFS group<br>Index year	
	–2018<br>N = 1270	2019–<br>N = 3600	–2018<br>N = 672	2019–<br>N = 2003	–2018<br>N = 598	2019–<br>N = 1597
Sex, n (%)						
Male	293 (23.1)	897 (24.9)	148 (22.0)	475 (23.7)	145 (24.2)	422 (26.4)
Female	977 (76.9)	2703 (75.1)	524 (78.0)	1528 (76.3)	453 (75.8)	1175 (73.6)
Age, years						
Mean ± SD	76.9 ± 7.1	80.0 ± 6.6	78.2 ± 6.9	80.8 ± 6.5	75.4 ± 7.1	78.9 ± 6.5
Age categories, n (%)						
40–69 years	191 (15.0)	234 (6.5)	76 (11.3)	102 (5.1)	115 (19.2)	132 (8.3)
70–79 years	596 (46.9)	1353 (37.6)	299 (44.5)	666 (33.3)	297 (49.7)	687 (43.0)
≥ 80 years	483 (38.0)	2013 (55.9)	297 (44.2)	1235 (61.7)	186 (31.1)	778 (48.7)
Type of insurance, n (%)						
SHI	16 (1.3)	15 (0.4)	3 (0.4)	5 (0.2)	13 (2.2)	10 (0.6)
NHI	454 (35.7)	615 (17.1)	215 (32.0)	313 (15.6)	239 (40.0)	302 (18.9)
LSEHS	800 (63.0)	2970 (82.5)	454 (67.6)	1685 (84.1)	346 (57.9)	1285 (80.5)

LSEHS, latter-stage elderly health care system; NHI, national health insurance; PVP, percutaneous vertebroplasty; SD, standard deviation; SFS, spine fusion surgery; SHI, society-managed employment-based health insurance association

Supplemental Table 13 Subgroup analysis of factors associated with reoperation in the PVP group, by year of first spine surgery

	Index year							
	–2018				2019–			
Variable	N	Cumulative incidence % at Day 365 (95% CI)	Unadjusted hazard ratio (95% CI)	Adjusted hazard ratio (95% CI)	N	Cumulative incidence % at Day 365 (95% CI)	Unadjusted HR (95% CI)	Adjusted HR (95% CI)
Sex								
Male	148	7.12 (3.89, 12,86)	1.00	1.00	475	8.03 (5.77, 11.11)	1.00	1.00
Female	524	9.48 (7.23, 12.39)	1.40 (0.76, 2.57)	1.11 (0.58, 2.11)	1528	7.38 (6.07, 8.95)	0.94 (0.64, 1.36)	0.97 (0.65, 1.44)
Age categories								
40–69 years	76	9.30 (4.55, 18.52)	1.00	1.00	102	5.29 (2.23, 12.28)	1.00	1.00
70–79 years	299	8.47 (5.75, 12.39)	1.02 (0.49, 2.11)	1.14 (0.53, 2.42)	666	9.08 (6.98, 11.77)	1.64 (0.72, 3.70)	1.55 (0.68, 3.53)
≥ 80 years	297	9.38 (6.53, 13.39)	0.98 (0.47, 2.02)	1.11 (0.52, 2.37)	1235	6.84 (5.46, 8.56)	1.27 (0.57, 2.83)	1.15 (0.51, 2.60)
Type of insurance								
SHI	3	0.00 (0.00, 0.00)	1.70 (0.10, 28.92)	-	5	0.00 (0.00, 0.00)	1.13 (0.07, 18.93)	-
NHI	215	7.13 (4.27, 11.77)	1.00	-	313	6.55 (4.22, 10.11)	1.00	-
LSEHS	454	9.91 (7.47, 13.09)	1.27 (0.75, 2.13)	-	1685	7.76 (6.48, 9.30)	1.12 (0.71, 1.76)	-
Degenerative spine diseases								
No	370	6.19 (4.12, 9.25)	1.00	1.00	1160	6.61 (5.21, 8.37)	1.00	1.00
Yes	302	12.39 (9.09, 16.77)	1.49 (0.95, 2.34)	1.40 (0.88, 2.22)	843	8.82 (6.96, 11.14)	1.39 (1.01, 1.92)	1.31 (0.94, 1.83)
Hip or knee osteoarthritis								
No	632	8.38 (6.43, 10.88)	1.00	-	1834	7.68 (6.46, 9.13)	1.00	-
Yes	40	18.44 (9.21, 34.94)	2.40 (1.17, 4.93)	-	169	5.94 (3.11, 11.20)	0.90 (0.49, 1.65)	-
Hyperparathyroidism								

No	660	8.51 (6.58, 10.97)	1.00	-	1971	7.65 (6.47, 9.04)	1.00	-
Yes	12	33.33 (14.03, 66.30)	3.71 (1.42, 9.68)	-	32	0.00 (0.00, 0.00)	0.21 (0.01, 3.46)	-
Hypoparathyroidism								
No	672	8.98 (7.01, 11.47)	1.00	-	2001	7.54 (6.38, 8.91)	1.00	-
Yes	0	-	-	-	2	NE	3.87 (0.24, 62.92)	-
Hyperthyroidism								
No	654	8.93 (6.94, 11.45)	1.00	-	1962	7.48 (6.31, 8.86)	1.00	-
Yes	18	11.11 (2.90, 37.58)	1.78 (0.61, 5.25)	-	41	10.17 (3.93, 24.92)	1.55 (0.60, 3.98)	-
Hypothyroidism								
No	629	9.09 (7.05, 11.68)	1.00	-	1837	7.38 (6.18, 8.80)	1.00	-
Yes	43	7.09 (2.34, 20.40)	0.93 (0.36, 2.42)	-	166	9.27 (5.52, 15.37)	1.27 (0.74, 2.19)	-
Chronic kidney disease								
No	540	9.60 (7.36, 12.47)	1.00	-	1597	7.86 (6.54, 9.44)	1.00	-
Non-dialysis[a]	121	5.15 (2.34, 11.11)	0.74 (0.38, 1.43)	-	381	6.61 (4.42, 9.83)	0.90 (0.59, 1.38)	-
Dialysis	11	20.00 (5.41, 59.13)	1.99 (0.55, 7.12)	-	25	0.00 (0.00, 0.00)	0.27 (0.02, 4.34)	-
Diabetes mellitus								
No	395	9.78 (7.18, 13.26)	1.00	-	1159	7.27 (5.79, 9.12)	1.00	-
Yes	277	7.83 (5.17, 11.77)	0.94 (0.59, 1.49)	-	844	7.90 (6.17, 10.08)	1.30 (0.94, 1.80)	-
Parkinson's disease								
No	625	8.99 (6.95, 11.57)	1.00	-	1893	7.21 (6.05, 8.59)	1.00	-
Yes	47	8.99 (3.46, 22.25)	0.84 (0.32, 2.19)	-	110	12.93 (7.48, 21.87)	1.62 (0.91, 2.90)	-
Hypertension								
No	206	9.71 (6.30, 14.82)	1.00	-	512	7.46 (5.30, 10.46)	1.00	-
Yes	466	8.66 (6.40, 11.67)	1.08 (0.66, 1.76)	-	1491	7.54 (6.22, 9.12)	1.12 (0.77, 1.63)	-

Dyslipidemia								
No	318	8.71 (6.01, 12.54)	1.00	-	836	7.08 (5.40, 9.26)	1.00	-
Yes	354	9.25 (6.63, 12.83)	1.26 (0.80, 1.98)	-	1167	7.84 (6.33, 9.69)	1.05 (0.75, 1.45)	-
Myocardial infarction								
No	651	9.11 (7.10, 11.65)	1.00	-	1934	7.57 (6.38, 8.96)	1.00	-
Yes	21	4.76 (0.68, 29.28)	1.54 (0.52, 4.52)	-	69	6.60 (2.51, 16.77)	0.92 (0.36, 2.37)	-
Congestive heart failure								
No	487	9.21 (6.91, 12.22)	1.00	-	1334	7.08 (5.71, 8.77)	1.00	-
Yes	185	8.34 (5.11, 13.46)	1.10 (0.67, 1.81)	-	669	8.44 (6.46, 10.99)	1.24 (0.89, 1.73)	-
Cerebrovascular disease								
No	463	9.22 (6.87, 12.32)	1.00	-	1377	7.38 (6.02, 9.02)	1.00	-
Yes	209	8.42 (5.31, 13.20)	1.06 (0.65, 1.71)	-	626	7.90 (5.85, 10.62)	1.06 (0.75, 1.50)	-
Dementia								
No	594	9.29 (7.18, 11.99)	1.00	-	1715	7.19 (5.96, 8.66)	1.00	-
Yes	78	6.55 (2.78, 15.03)	0.66 (0.29, 1.48)	-	288	9.57 (6.55, 13.88)	1.36 (0.89, 2.07)	-
Chronic pulmonary disease								
No	467	7.76 (5.63, 10.65)	1.00	-	1403	7.53 (6.16, 9.19)	1.00	-
Yes	205	11.76 (7.97, 17.18)	1.44 (0.91, 2.29)	-	600	7.55 (5.57, 10.19)	1.07 (0.75, 1.51)	-
Liver disease								
No	522	10.34 (7.97, 13.35)	1.00	-	1502	7.10 (5.81, 8.66)	1.00	-
Mild[b]	144	4.35 (1.98, 9.45)	0.56 (0.29, 1.08)	-	478	8.48 (6.15, 11.64)	1.26 (0.87, 1.81)	-
Moderate or severe	6	0.00 (0.00, 0.00)	0.78 (0.05, 13.04)	-	23	16.97 (5.68, 44.66)	3.12 (1.21, 8.07)	-
Rheumatologic disease								
No	605	8.58 (6.57, 11.17)	1.00	-	1836	7.52 (6.31, 8.94)	1.00	-

Yes	67	12.56 (6.47, 23.61)	1.96 (1.09, 3.54)	-	167	7.76 (4.31, 13.78)	1.11 (0.63, 1.94)	-
Malignant tumor								
No	576	8.97 (6.87, 11.67)	1.00	-	1661	7.69 (6.40, 9.22)	1.00	-
Yes	96	9.01 (4.59, 17.26)	1.30 (0.70, 2.38)	-	342	6.74 (4.43, 10.18)	0.90 (0.57, 1.41)	-
Charlson Comorbidity Index								
0–2	427	9.72 (7.22, 13.02)	1.00	-	1115	7.14 (5.64, 9.01)	1.00	-
≥ 3	245	7.67 (4.90, 11.91)	0.93 (0.58, 1.50)	-	888	8.02 (6.32, 10.16)	1.23 (0.89, 1.70)	-
Psychotropic drugs								
No	335	5.64 (3.59, 8.81)	1.00	1.00	1073	6.34 (4.94, 8.13)	1.00	1.00
Yes	337	12.29 (9.16, 16.38)	1.58 (1.00, 2.50)	1.47 (0.91, 2.38)	930	8.89 (7.10, 11.12)	1.29 (0.93, 1.79)	1.29 (0.93, 1.79)
Narcotics								
No	661	8.79 (6.83, 11.27)	1.00	-	1984	7.51 (6.34, 8.88)	1.00	-
Yes	11	22.22 (6.07, 63.52)	2.08 (0.59, 7.41)	-	19	10.53 (2.74, 35.92)	1.77 (0.51, 6.22)	-
Anticonvulsants								
No	507	7.99 (5.90, 10.78)	1.00	-	1556	6.49 (5.29, 7.96)	1.00	-
Yes	165	12.04 (7.84, 18.25)	1.41 (0.86, 2.30)	-	447	11.02 (8.25, 14.63)	1.59 (1.12, 2.26)	-
Muscle relaxants								
No	563	7.96 (5.96, 10.59)	1.00	-	1787	7.51 (6.29, 8.96)	1.00	-
Yes	109	14.34 (8.90, 22.68)	1.50 (0.87, 2.60)	-	216	7.63 (4.56, 12.65)	1.03 (0.62, 1.72)	-
Proton pump inhibitor								
No	338	7.34 (4.98, 10.76)	1.00	-	937	6.61 (5.06, 8.60)	1.00	-
Yes	334	10.66 (7.73, 14.61)	1.39 (0.88, 2.19)	-	1066	8.34 (6.72, 10.33)	1.39 (0.99, 1.93)	-
Anticoagulants (warfarin and heparin)								
No	627	8.82 (6.81, 11.40)	1.00	-	1894	7.67 (6.47, 9.09)	1.00	-

Yes	45	11.11 (4.78, 24.66)	1.33 (0.59, 2.99)	-	109	4.91 (2.07, 11.44)	0.84 (0.38, 1.84)	-
Hormone replacement therapy (estrogen-only)								
No	666	9.07 (7.08, 11.57)	1.00	-	1991	7.46 (6.30, 8.83)	1.00	-
Yes	6	0.00 (0.00, 0.00)	0.64 (0.04, 10.42)	-	12	19.79 (5.21, 59.74)	3.36 (0.96, 11.78)	-
Endocrine therapy except estrogen for breast cancer and prostate cancer								
No	660	9.14 (7.14, 11.67)	1.00	-	1953	7.67 (6.48, 9.07)	1.00	-
Yes	12	0.00 (0.00, 0.00)	1.08 (0.21, 5.48)	-	50	2.17 (0.31, 14.45)	0.64 (0.18, 2.25)	-
Glucocorticoids mean dose[c]								
≥ 0, < 1 mg/day	632	8.56 (6.59, 11.09)	1.00	1.00	1869	7.43 (6.24, 8.84)	1.00	1.00
≥ 1, < 5 mg/day	19	10.53 (2.74, 35.92)	2.46 (0.94, 6.46)	2.72 (0.99, 7.44)	73	7.50 (3.17, 17.17)	1.25 (0.57, 2.77)	1.07 (0.48, 2.40)
≥ 5, < 7.5 mg/day	11	18.18 (4.88, 55.26)	2.37 (0.66, 8.53)	1.69 (0.43, 6.60)	22	21.95 (8.48, 49.99)	3.19 (1.24, 8.23)	3.24 (1.24, 8.47)
≥ 7.5 mg/day	10	20.00 (5.41, 59.13)	2.33 (0.65, 8.36)	2.67 (0.70, 10.17)	39	5.13 (1.31, 18.98)	1.23 (0.42, 3.57)	1.14 (0.39, 3.34)
History of fractures other than VF								
No	594	8.44 (6.43, 11.05)	1.00	1.00	1702	7.54 (6.29, 9.04)	1.00	1.00
All hip fracture	8	25.00 (6.91, 68.52)	3.89 (1.08, 14.03)	2.91 (0.75, 11.32)	54	14.09 (6.95, 27.41)	1.90 (0.90, 3.98)	1.88 (0.89, 4.00)
NHNV fracture	64	12.75 (6.58, 23.89)	1.89 (1.02, 3.49)	1.88 (1.00, 3.55)	225	6.21 (3.55, 10.75)	0.80 (0.45, 1.40)	0.73 (0.41, 1.29)
Both[d]	6	0.00 (0.00, 0.00)	0.76 (0.05, 12.80)	0.69 (0.04, 12.39)	22	4.55 (0.65, 28.13)	0.88 (0.17, 4.46)	0.86 (0.17, 4.40)
No. of beds at medical institutions performing surgery								
≤ 19	53	5.74 (1.89, 16.75)	0.63 (0.23, 1.69)	-	39	5.56 (1.42, 20.43)	0.90 (0.25, 3.23)	-
20–199	291	10.02 (7.02, 14.19)	1.00	-	868	7.52 (5.80, 9.71)	1.00	-
200–399	65	10.87 (5.34, 21.47)	1.00 (0.47, 2.13)	-	586	6.70 (4.83, 9.25)	0.98 (0.66, 1.45)	-
≥ 400	263	8.07 (5.28, 12.25)	0.79 (0.48, 1.30)	-	510	8.66 (6.37, 11.72)	1.12 (0.76, 1.67)	-
missing	0	-	-	-	0	-	-	-
Pre preoperative osteoporosis medications status								

Untreated[e]	399	7.02 (4.86, 10.07)	1.00	1.00	1277	6.72 (5.39, 8.36)	1.00	1.00
Previously treated	273	11.77 (8.42, 16.32)	1.71 (1.09, 2.69)	1.23 (0.73, 2.05)	726	8.97 (6.92, 11.58)	1.16 (0.83, 1.61)	1.18 (0.79, 1.75)
Drug classes for perioperative osteoporosis medications[f]								
No	118	4.36 (1.84, 10.17)	1.00	1.00	411	10.84 (7.95, 14.70)	1.00	1.00
Anabolic agents[g]	249	10.47 (7.19, 15.11)	2.37 (1.01, 5.60)	2.16 (0.89, 5.28)	721	5.99 (4.38, 8.16)	0.51 (0.33, 0.79)	0.48 (0.30, 0.75)
Antiresorptive agents	230	9.94 (6.66, 14.71)	2.43 (1.03, 5.75)	2.17 (0.87, 5.39)	574	8.22 (6.14, 10.96)	0.85 (0.56, 1.28)	0.78 (0.48, 1.25)
Combination of two or more[h]	4	25.00 (3.95, 87.21)	15.36 (3.49, 67.68)	16.97 (3.27, 87.98)	4	0.00 (0.00, 0.00)	1.02 (0.06, 17.02)	0.89 (0.05, 15.42)
Bone metabolism-regulating drugs only[i]	71	7.20 (3.06, 16.44)	1.88 (0.65, 5.48)	2.02 (0.68, 5.94)	293	5.73 (3.29, 9.88)	0.50 (0.28, 0.90)	0.50 (0.28, 0.91)

Using Firth's method for parameter estimates. CI, confidence interval; HR, hazard ratio; LSEHS, latter-stage elderly healthcare system; NE, not estimable; NHI, national health insurance; NHNV, non-hip non-vertebral; PVP, percutaneous vertebroplasty; SHI, society-managed employment-based health insurance association; VF, vertebral fracture

[a] Patients with chronic kidney disease, excluding dialysis

[b] Patients with mild liver disease, excluding moderate or severe liver disease

[c] As prednisolone equivalent mg/day. The mean dose per day calculated from the total dose per year in patients who were prescribed oral glucocorticoids for at least 90 days during the baseline period

[d] Patients with a history of both all hip fracture together with NHNV during the baseline period

[e] Untreated or bone metabolism-regulating drugs only

[f] "Anabolic agents" and "antiresorptive agents" also include patients who use those agents in combination with "bone metabolism-regulating drugs"

[g] Anabolic agents: PTH and Rmab

[h] Excluding bone metabolism-regulating drugs

[i] A general term that includes active vitamin D, vitamin K, and calcium

Supplemental Table 14 Subgroup analysis of factors associated with reoperation in the SFS group, by year of first spine surgery

Variable	Index year							
	–2018				2019–			
	N	Cumulative incidence % at Day 365 (95% CI)	Unadjusted HR (95% CI)	Adjusted HR (95% CI)	N	Cumulative incidence % at Day 365 (95% CI)	Unadjusted HR (95% CI)	Adjusted HR (95% CI)
Sex								
Male	145	5.66 (2.87, 11.01)	1.00	1.00	422	7.14 (4.83, 10.50)	1.00	1.00
Female	453	4.82 (3.17, 7.31)	0.87 (0.44, 1.72)	0.90 (0.45, 1.81)	1175	6.00 (4.69, 7.68)	0.92 (0.59, 1.43)	0.94 (0.59, 1.48)
Age categories								
40–69 years	115	7.10 (3.61, 13.69)	1.00	1.00	132	4.10 (1.72, 9.63)	1.00	1.00
70–79 years	297	3.52 (1.91, 6.45)	0.46 (0.23, 0.95)	0.48 (0.23, 1.01)	687	7.01 (5.21, 9.40)	1.13 (0.56, 2.27)	1.17 (0.58, 2.36)
≥ 80 years	186	6.05 (3.39, 10.66)	0.58 (0.27, 1.23)	0.62 (0.27, 1.39)	778	6.06 (4.42, 8.28)	0.85 (0.41, 1.73)	0.86 (0.41, 1.77)
Type of insurance								
SHI	13	15.38 (4.09, 48.78)	1.97 (0.52, 7.43)	-	10	0.00 (0.00, 0.00)	0.54 (0.03, 9.29)	-
NHI	239	6.20 (3.71, 10.26)	1.00	-	302	4.29 (2.45, 7.46)	1.00	-
LSEHS	346	3.84 (2.25, 6.53)	0.48 (0.26, 0.90)	-	1285	6.85 (5.47, 8.57)	1.04 (0.63, 1.72)	-
Degenerative spine diseases								
No	162	7.05 (3.96, 12.37)	1.00	-	473	6.63 (4.56, 9.59)	1.00	-
Yes	436	4.28 (2.72, 6.71)	0.65 (0.35, 1.22)	-	1124	6.16 (4.78, 7.91)	0.90 (0.59, 1.38)	-
Hip or knee osteoarthritis								
No	538	5.02 (3.44, 7.29)	1.00	-	1401	6.58 (5.29, 8.16)	1.00	-
Yes	60	5.15 (1.69, 15.14)	1.24 (0.50, 3.06)	-	196	4.29 (2.05, 8.88)	0.67 (0.33, 1.35)	-
Hyperparathyroidism								
No	581	4.84 (3.34, 6.98)	1.00	1.00	1556	6.28 (5.08, 7.74)	1.00	1.00

Yes	17	11.76 (3.08, 39.40)	5.08 (2.05, 12.54)	3.40 (1.24, 9.32)	41	6.93 (1.71, 25.81)	1.42 (0.49, 4.14)	1.49 (0.50, 4.51)
Hypoparathyroidism								
No	598	5.03 (3.52, 7.16)	1.00	-	1596	6.30 (5.11, 7.76)	1.00	-
Yes	0	-	-	-	1	0.00 (0.00, 0.00)	5.64 (0.34, 92.59)	-
Hyperthyroidism								
No	582	5.17 (3.62, 7.35)	1.00	-	1555	6.41 (5.19, 7.90)	1.00	-
Yes	16	0.00 (0.00, 0.00)	1.24 (0.24, 6.46)	-	42	2.38 (0.34, 15.72)	0.92 (0.26, 3.26)	-
Hypothyroidism								
No	560	5.01 (3.46, 7.22)	1.00	-	1433	6.32 (5.07, 7.86)	1.00	-
Yes	38	5.26 (1.34, 19.44)	1.76 (0.65, 4.71)	-	164	6.15 (3.07, 12.10)	1.04 (0.55, 1.98)	-
Chronic kidney disease								
No	474	4.60 (3.02, 6.98)	1.00	-	1226	6.36 (5.01, 8.07)	1.00	-
Non-dialysis[a]	113	5.52 (2.52, 11.89)	0.89 (0.40, 1.98)	-	343	6.29 (4.04, 9.72)	0.99 (0.61, 1.61)	-
Dialysis	11	18.18 (4.88, 55.26)	3.30 (0.89, 12.19)	-	28	3.85 (0.55, 24.31)	0.86 (0.17, 4.40)	-
Diabetes mellitus								
No	360	5.75 (3.75, 8.78)	1.00	-	919	6.78 (5.19, 8.83)	1.00	-
Yes	238	3.90 (2.05, 7.37)	0.76 (0.40, 1.43)	-	678	5.64 (4.02, 7.89)	0.89 (0.60, 1.33)	-
Parkinson's disease								
No	555	4.51 (3.05, 6.66)	1.00	1.00	1495	5.80 (4.62, 7.27)	1.00	1.00
Yes	43	11.85 (5.11, 26.17)	3.31 (1.55, 7.06)	2.50 (1.09, 5.69)	102	13.53 (8.06, 22.23)	2.34 (1.31, 4.16)	2.10 (1.16, 3.78)
Hypertension								
No	177	5.20 (2.74, 9.76)	1.00	-	361	6.00 (3.78, 9.44)	1.00	-
Yes	421	4.96 (3.23, 7.58)	0.71 (0.38, 1.32)	-	1236	6.37 (5.04, 8.04)	0.91 (0.58, 1.44)	-
Dyslipidemia								

No	259	4.78 (2.74, 8.27)	1.00	-	645	7.27 (5.35, 9.84)	1.00	-
Yes	339	5.23 (3.28, 8.28)	1.06 (0.58, 1.94)	-	952	5.64 (4.24, 7.49)	0.72 (0.49, 1.07)	-
Myocardial infarction								
No	579	5.19 (3.63, 7.38)	1.00	-	1541	6.30 (5.10, 7.79)	1.00	-
Yes	19	0.00 (0.00, 0.00)	0.35 (0.02, 5.90)	-	56	6.18 (1.99, 18.32)	1.31 (0.50, 3.38)	-
Congestive heart failure								
No	414	4.78 (3.07, 7.39)	1.00	-	1048	5.98 (4.59, 7.77)	1.00	-
Yes	184	5.60 (3.05, 10.16)	0.99 (0.52, 1.90)	-	549	6.92 (4.91, 9.73)	1.27 (0.85, 1.90)	-
Cerebrovascular disease								
No	462	6.31 (4.40, 9.01)	1.00	-	1097	5.61 (4.30, 7.31)	1.00	-
Yes	136	0.77 (0.11, 5.33)	0.56 (0.24, 1.29)	-	500	7.84 (5.59, 10.94)	1.18 (0.78, 1.78)	-
Dementia								
No	555	5.22 (3.63, 7.47)	1.00	-	1426	6.33 (5.07, 7.88)	1.00	-
Yes	43	2.56 (0.37, 16.84)	0.79 (0.22, 2.89)	-	171	5.96 (3.11, 11.25)	0.92 (0.47, 1.80)	-
Chronic pulmonary disease								
No	421	4.94 (3.22, 7.56)	1.00	-	1136	6.84 (5.39, 8.67)	1.00	-
Yes	177	5.26 (2.77, 9.88)	1.17 (0.62, 2.20)	-	461	4.96 (3.21, 7.61)	0.83 (0.53, 1.31)	-
Liver disease								
No	459	5.44 (3.68, 8.01)	1.00	-	1137	6.52 (5.11, 8.29)	1.00	-
Mild[b]	135	3.76 (1.58, 8.80)	0.67 (0.30, 1.49)	-	449	5.87 (3.88, 8.83)	0.96 (0.62, 1.49)	-
Moderate or severe	4	0.00 (0.00, 0.00)	2.37 (0.14, 40.86)	-	11	0.00 (0.00, 0.00)	0.78 (0.05, 12.92)	-
Rheumatologic disease								
No	511	5.50 (3.80, 7.92)	1.00	-	1407	5.84 (4.63, 7.35)	1.00	-
Yes	87	2.31 (0.58, 8.93)	0.66 (0.25, 1.77)	-	190	9.53 (5.90, 15.20)	1.81 (1.10, 2.97)	-

Malignant tumor								
No	529	4.32 (2.87, 6.49)	1.00	-	1355	6.10 (4.85, 7.67)	1.00	-
Yes	69	10.50 (5.14, 20.80)	2.01 (0.94, 4.28)	-	242	7.36 (4.44, 12.06)	1.15 (0.67, 1.98)	-
Charlson Comorbidity Index								
0–2	364	4.01 (2.39, 6.68)	1.00	-	879	5.73 (4.26, 7.69)	1.00	-
≥ 3	234	6.62 (4.04, 10.74)	1.28 (0.70, 2.33)	-	718	7.00 (5.21, 9.38)	1.26 (0.85, 1.86)	-
Psychotropic drugs								
No	238	7.05 (4.38, 11.26)	1.00	-	647	5.56 (3.92, 7.86)	1.00	-
Yes	360	3.71 (2.17, 6.31)	0.67 (0.37, 1.22)	-	950	6.80 (5.24, 8.81)	1.12 (0.75, 1.68)	-
Narcotics								
No	586	5.13 (3.59, 7.30)	1.00	-	1588	6.34 (5.14, 7.80)	1.00	-
Yes	12	0.00 (0.00, 0.00)	1.80 (0.35, 9.36)	-	9	0.00 (0.00, 0.00)	0.75 (0.05, 12.24)	-
Anticonvulsants								
No	291	5.07 (3.03, 8.41)	1.00	-	848	5.64 (4.15, 7.63)	1.00	-
Yes	307	5.00 (3.04, 8.15)	1.04 (0.57, 1.89)	-	749	6.99 (5.24, 9.29)	1.24 (0.84, 1.84)	-
Muscle relaxants								
No	502	4.98 (3.36, 7.34)	1.00	-	1399	6.26 (5.00, 7.82)	1.00	-
Yes	96	5.30 (2.24, 12.28)	1.03 (0.47, 2.28)	-	198	6.57 (3.67, 11.61)	1.15 (0.66, 2.01)	-
Proton pump inhibitor								
No	229	4.98 (2.79, 8.81)	1.00	-	627	6.67 (4.80, 9.24)	1.00	-
Yes	369	5.06 (3.22, 7.92)	1.16 (0.62, 2.18)	-	970	6.06 (4.62, 7.93)	0.88 (0.59, 1.31)	-
Anticoagulants (warfarin and heparin)								
No	550	5.28 (3.68, 7.56)	1.00	-	1472	6.24 (5.01, 7.76)	1.00	-
Yes	48	2.13 (0.30, 14.16)	0.70 (0.19, 2.56)	-	125	7.12 (3.56, 13.97)	1.29 (0.68, 2.45)	-

Hormone replacement therapy (estrogen-only)								
No	593	5.07 (3.55, 7.22)	1.00	-	1590	6.33 (5.13, 7.79)	1.00	-
Yes	5	0.00 (0.00, 0.00)	3.70 (0.71, 19.24)	-	7	0.00 (0.00, 0.00)	0.90 (0.06, 14.78)	-
Endocrine therapy except estrogen for breast cancer and prostate cancer								
No	592	4.91 (3.42, 7.04)	1.00	-	1560	6.29 (5.09, 7.77)	1.00	-
Yes	6	16.67 (2.53, 72.69)	3.60 (0.69, 18.73)	-	37	5.84 (1.49, 21.50)	1.23 (0.35, 4.36)	-
Glucocorticoids mean dose[c]								
≥ 0, < 1, mg/day	521	5.38 (3.72, 7.75)	1.00	-	1454	5.98 (4.78, 7.47)	1.00	-
≥ 1, < 5, mg/day	34	3.03 (0.43, 19.63)	0.59 (0.11, 3.12)	-	78	10.92 (5.29, 21.79)	1.86 (0.91, 3.79)	-
≥ 5, < 7.5, mg/day	18	0.00 (0.00, 0.00)	0.38 (0.02, 6.52)	-	33	11.93 (3.73, 34.61)	2.95 (1.13, 7.70)	-
≥ 7.5, mg/day	25	4.00 (0.57, 25.16)	1.81 (0.59, 5.55)	-	32	3.13 (0.45, 20.18)	1.76 (0.60, 5.20)	-
History of fractures other than VF								
No	507	5.32 (3.65, 7.72)	1.00	-	1302	6.30 (4.99, 7.93)	1.00	-
All hip fracture	16	0.00 (0.00, 0.00)	0.38 (0.02, 6.60)	-	35	2.86 (0.41, 18.60)	1.11 (0.31, 3.97)	-
NHNV fracture	73	4.27 (1.40, 12.67)	1.04 (0.42, 2.59)	-	237	7.08 (4.22, 11.74)	1.09 (0.64, 1.87)	-
Both[d]	2	0.00 (0.00, 0.00)	2.99 (0.17, 51.99)	-	23	4.35 (0.62, 27.07)	1.03 (0.20, 5.29)	-
No. of beds at medical institutions performing surgery								
≤ 19	14	0.00 (0.00, 0.00)	0.57 (0.03, 10.86)	0.45 (0.02, 8.87)	16	7.14 (1.04, 40.92)	2.27 (0.40, 12.94)	2.27 (0.39, 13.03)
20–199	106	1.98 (0.50, 7.68)	1.00	1.00	262	3.03 (1.43, 6.35)	1.00	1.00
200–399	133	3.91 (1.65, 9.14)	1.34 (0.49, 3.65)	1.26 (0.43, 3.66)	551	7.47 (5.36, 10.36)	2.10 (1.03, 4.28)	1.64 (0.80, 3.36)
≥ 400	345	6.65 (4.43, 9.93)	1.41 (0.59, 3.39)	1.37 (0.53, 3.53)	768	6.50 (4.85, 8.67)	1.77 (0.88, 3.58)	1.28 (0.63, 2.60)
missing	0	-	-	-	0	-	-	-
Details of the surgical procedure								
Posterior spinal fusion	398	5.19 (3.38, 7.93)	1.00	1.00	1137	5.93 (4.60, 7.63)	1.00	1.00

Anterior spinal fusion[e]	193	4.86 (2.55, 9.13)	1.42 (0.76, 2.63)	1.22 (0.63, 2.34)	379	7.79 (5.27, 11.43)	1.27 (0.81, 1.99)	1.16 (0.73, 1.85)
Combined PVP	7	0.00 (0.00, 0.00)	3.21 (0.60, 17.13)	2.43 (0.39, 15.13)	81	4.53 (1.46, 13.54)	1.13 (0.47, 2.70)	1.09 (0.45, 2.63)
Length of fusion area								
1 level	36	2.86 (0.41, 18.60)	0.47 (0.09, 2.47)	0.77 (0.13, 4.68)	65	0.00 (0.00, 0.00)	0.10 (0.01, 1.65)	0.13 (0.01, 2.15)
2 levels	113	1.80 (0.45, 7.02)	0.79 (0.35, 1.76)	0.89 (0.38, 2.04)	315	5.57 (3.36, 9.15)	0.67 (0.39, 1.16)	0.72 (0.41, 1.26)
≥ 3 levels	449	6.03 (4.14, 8.73)	1.00	1.00	1217	6.83 (5.43, 8.58)	1.00	1.00
Pre-preoperative osteoporosis medications status								
Untreated[f]	279	4.84 (2.84, 8.19)	1.00	-	780	5.72 (4.20, 7.77)	1.00	-
Previously treated	319	5.20 (3.21, 8.35)	1.19 (0.65, 2.19)	-	817	6.84 (5.15, 9.06)	1.19 (0.80, 1.77)	-
Drug classes for perioperative osteoporosis medications[g]								
No	115	1.75 (0.44, 6.80)	1.00	1.00	301	5.62 (3.41, 9.21)	1.00	1.00
Anabolic agents[h]	273	6.13 (3.80, 9.82)	2.40 (0.85, 6.74)	2.39 (0.83, 6.86)	728	7.13 (5.35, 9.48)	1.29 (0.73, 2.26)	1.22 (0.69, 2.16)
Antiresorptive agents	156	6.66 (3.64, 12.03)	2.28 (0.76, 6.84)	2.32 (0.75, 7.15)	391	6.25 (4.04, 9.62)	1.18 (0.63, 2.22)	1.29 (0.68, 2.45)
Combination of two or more[i]	1	0.00 (0.00, 0.00)	11.32 (0.56, 229.04)	5.29 (0.18, 152.86)	3	33.33 (5.48, 94.59)	10.26 (1.88, 55.80)	11.76 (2.04, 67.85)
Bone metabolism-regulating drugs only[j]	53	1.89 (0.27, 12.65)	1.18 (0.24, 5.79)	1.12 (0.22, 5.56)	174	3.63 (1.50, 8.68)	0.91 (0.40, 2.11)	0.91 (0.39, 2.11)

Using Firth’s method for parameter estimates. CI, confidence interval; HR, hazard ratio; LSEHS, latter-stage elderly healthcare system; NHI, national health insurance; NHNV, non-hip non-vertebral; PVP, percutaneous vertebroplasty; SFS, spine fusion surgery; SHI, society-managed employment-based health insurance association; VF, vertebral fracture

[a] Patients with chronic kidney disease, excluding dialysis

[b] Patients with mild liver disease, excluding moderate or severe liver disease

[c] As prednisolone equivalent mg/day. The mean dose per day calculated from the total dose per year in patients who were prescribed oral glucocorticoids for at least 90 days during the baseline period

[d] Patients with a history of both all hip fracture together with NHNV during the baseline period

[e] Including combined antero-posterior approaches

[f] Untreated or bone metabolism-regulating drugs only

[g] “Anabolic agents” and “antiresorptive agents” also include patients who use those agents in combination with “bone metabolism-regulating drugs”

[h] Anabolic agents: PTH and Rmab

[i] Excluding bone metabolism-regulating drugs

[j] A general term that includes active vitamin D, vitamin K, and calcium

Supplemental Table 15 Subgroup analysis of factors associated with subsequent VF in the PVP group, by year of first spine surgery

	Index year							
	–2018				2019–			
Variable	N	Cumulative incidence % at Day 365 (95% CI)	Unadjusted HR (95% CI)	Adjusted HR (95% CI)	N	Cumulative incidence % at Day 365 (95% CI)	Unadjusted HR (95% CI)	Adjusted HR (95% CI)
Sex								
Male	148	7.86 (4.42, 13.76)	1.00	1.00	475	6.85 (4.78, 9.77)	1.00	1.00
Female	524	9.69 (7.41, 12.62)	1.59 (0.87, 2.91)	1.36 (0.73, 2.54)	1528	7.08 (5.82, 8.61)	1.04 (0.70, 1.56)	1.08 (0.71, 1.64)
Age categories								
40–69 years	76	7.95 (3.65, 16.84)	1.00	1.00	102	6.31 (2.88, 13.53)	1.00	1.00
70–79 years	299	10.01 (7.01 14.19)	1.42 (0.67, 3.00)	1.42 (0.67, 3.04)	666	8.18 (6.20, 10.77)	1.19 (0.55, 2.58)	1.14 (0.53, 2.49)
≥ 80 years	297	8.99 (6.21, 12.92)	1.21 (0.57, 2.56)	1.25 (0.58, 2.67)	1235	6.41 (5.10, 8.05)	1.03 (0.49, 2.19)	0.99 (0.46, 2.12)
Type of insurance								
SHI	3	0.00 (0.00, 0.00)	1.63 (0.10, 27.57)	-	5	0.00 (0.00, 0.00)	1.15 (0.07, 19.27)	-
NHI	215	7.57 (4.62, 12.29)	1.00	-	313	7.18 (4.73, 10.83)	1.00	-
LSEHS	454	10.14 (7.67, 13.35)	1.35 (0.82, 2.24)	-	1685	7.03 (5.82, 8.48)	1.01 (0.64, 1.60)	-
Degenerative spine diseases								
No	370	6.45 (4.33, 9.55)	1.00	-	1160	6.52 (5.15, 8.25)	1.00	-
Yes	302	12.78 (9.42, 17.22)	1.43 (0.94, 2.19)	-	843	7.72 (6.01, 9.91)	1.20 (0.85, 1.68)	-
Hip or knee osteoarthritis								
No	632	8.52 (6.56, 11.04)	1.00	-	1834	7.16 (5.99, 8.55)	1.00	-
Yes	40	22.52 (11.83, 40.38)	2.39 (1.21, 4.71)	-	169	5.63 (2.96, 10.56)	0.89 (0.47, 1.67)	-
Hyperparathyroidism								

No	660	8.83 (6.86, 11.33)	1.00	-	1971	7.13 (6.01, 8.47)	1.00	-
Yes	12	33.33 (14.03, 66.30)	4.33 (1.81, 10.32)	-	32	0.00 (0.00, 0.00)	0.23 (0.01, 3.71)	-
Hypoparathyroidism								
No	672	9.29 (7.29, 11.81)	1.00	-	2001	7.03 (5.92, 8.34)	1.00	-
Yes	0	-	-	-	2	NE	4.01 (0.25, 65.32)	-
Hyperthyroidism								
No	654	9.25 (7.22, 11.80)	1.00	-	1962	6.96 (5.84, 8.28)	1.00	-
Yes	18	11.11 (2.90, 37.58)	1.57 (0.54, 4.61)	-	41	10.17 (3.93, 24.92)	1.67 (0.65, 4.28)	-
Hypothyroidism								
No	629	9.08 (7.04, 11.66)	1.00	-	1837	6.74 (5.61, 8.09)	1.00	-
Yes	43	12.45 (5.35, 27.50)	1.06 (0.44, 2.52)	-	166	10.20 (6.19, 16.58)	1.47 (0.87, 2.50)	-
Chronic kidney disease								
No	540	9.40 (7.18, 12.25)	1.00	-	1597	7.29 (6.03, 8.81)	1.00	-
Non-dialysis[a]	121	6.98 (3.54, 13.50)	0.89 (0.50, 1.61)	-	381	6.32 (4.19, 9.48)	0.94 (0.61, 1.44)	-
Dialysis	11	29.29 (10.47, 66.27)	2.88 (0.97, 8.51)	-	25	0.00 (0.00, 0.00)	0.29 (0.02, 4.65)	-
Diabetes mellitus								
No	395	11.35 (8.54, 15.01)	1.00	-	1159	6.68 (5.27, 8.46)	1.00	-
Yes	277	6.35 (3.99, 10.02)	0.81 (0.52, 1.26)	-	844	7.50 (5.84, 9.61)	1.30 (0.93, 1.82)	-
Parkinson's disease								
No	625	8.98 (6.95, 11.57)	1.00	-	1893	6.67 (5.57, 7.99)	1.00	-
Yes	47	13.53 (6.30, 27.73)	1.34 (0.63, 2.84)	-	110	12.93 (7.48, 21.87)	1.75 (0.98, 3.14)	-
Hypertension								
No	206	8.70 (5.50, 13.64)	1.00	-	512	6.51 (4.54, 9.29)	1.00	-
Yes	466	9.55 (7.17, 12.67)	1.54 (0.93, 2.56)	-	1491	7.20 (5.91, 8.74)	1.14 (0.77, 1.69)	-

Dyslipidemia								
No	318	9.37 (6.56, 13.30)	1.00	-	836	6.37 (4.80, 8.43)	1.00	-
Yes	354	9.25 (6.63, 12.83)	1.37 (0.89, 2.11)	-	1167	7.49 (6.02, 9.29)	1.07 (0.76, 1.51)	-
Myocardial infarction								
No	651	9.58 (7.52, 12.17)	1.00	-	1934	7.10 (5.97, 8.45)	1.00	-
Yes	21	0.00 (0.00, 0.00)	1.72 (0.66, 4.47)	-	69	4.66 (1.52, 13.78)	0.77 (0.26, 2.22)	-
Congestive heart failure								
No	487	9.39 (7.07, 12.42)	1.00	-	1334	6.58 (5.28, 8.20)	1.00	-
Yes	185	9.03 (5.62, 14.33)	1.00 (0.62, 1.61)	-	669	7.90 (6.00, 10.38)	1.23 (0.87, 1.74)	-
Cerebrovascular disease								
No	463	8.74 (6.46, 11.78)	1.00	-	1377	6.69 (5.42, 8.25)	1.00	-
Yes	209	10.53 (6.99, 15.70)	1.26 (0.81, 1.97)	-	626	7.78 (5.76, 10.45)	1.11 (0.78, 1.58)	-
Dementia								
No	594	8.74 (6.70, 11.38)	1.00	-	1715	6.60 (5.44, 8.00)	1.00	-
Yes	78	13.11 (7.28, 23.01)	1.04 (0.54, 1.99)	-	288	9.57 (6.55, 13.88)	1.48 (0.97, 2.27)	-
Chronic pulmonary disease								
No	467	8.41 (6.19, 11.38)	1.00	-	1403	6.99 (5.69, 8.59)	1.00	-
Yes	205	11.34 (7.61, 16.73)	1.41 (0.91, 2.18)	-	600	7.09 (5.19, 9.66)	1.03 (0.72, 1.49)	-
Liver disease								
No	522	10.14 (7.80, 13.13)	1.00	-	1502	6.53 (5.31, 8.02)	1.00	-
Mild[b]	144	6.54 (3.45, 12.22)	0.73 (0.41, 1.29)	-	478	8.45 (6.12, 11.60)	1.24 (0.85, 1.81)	-
Moderate or severe	6	0.00 (0.00, 0.00)	0.77 (0.05, 12.73)	-	23	10.05 (2.60, 34.66)	2.54 (0.87, 7.42)	-
Rheumatologic disease								
No	605	8.92 (6.87, 11.54)	1.00	-	1836	7.09 (5.93, 8.47)	1.00	-

Yes	67	12.70 (6.54, 23.85)	2.03 (1.17, 3.53)	-	167	6.33 (3.28, 12.03)	1.00 (0.54, 1.83)	-
Malignant tumor								
No	576	8.95 (6.86, 11.65)	1.00	-	1661	7.30 (6.06, 8.78)	1.00	-
Yes	96	11.65 (6.42, 20.64)	1.26 (0.70, 2.26)	-	342	5.63 (3.58, 8.80)	0.82 (0.51, 1.32)	-
Charlson Comorbidity Index								
0–2	427	9.44 (6.98, 12.69)	1.00	-	1115	6.69 (5.26, 8.49)	1.00	-
≥ 3	245	9.05 (5.99, 13.56)	0.98 (0.63, 1.53)	-	888	7.44 (5.81, 9.50)	1.15 (0.82, 1.61)	-
Psychotropic drugs								
No	335	6.26 (4.08, 9.53)	1.00	1.00	1073	5.91 (4.57, 7.62)	1.00	1.00
Yes	337	12.29 (9.16, 16.39)	1.54 (1.00, 2.38)	1.54 (0.99, 2.40)	930	8.30 (6.58, 10.44)	1.26 (0.90, 1.76)	1.28 (0.91, 1.79)
Narcotics								
No	661	9.27 (7.26, 11.81)	1.00	-	1984	6.99 (5.88, 8.31)	1.00	-
Yes	11	11.11 (1.64, 56.70)	1.04 (0.20, 5.25)	-	19	10.53 (2.74, 35.92)	1.92 (0.55, 6.76)	-
Anticonvulsants								
No	507	8.38 (6.24, 11.22)	1.00	-	1556	6.35 (5.16, 7.81)	1.00	-
Yes	165	12.14 (7.91, 18.39)	1.20 (0.75, 1.94)	-	447	9.36 (6.88, 12.67)	1.45 (1.00, 2.09)	-
Muscle relaxants								
No	563	8.32 (6.27, 10.99)	1.00	-	1787	7.04 (5.87, 8.44)	1.00	-
Yes	109	14.37 (8.91, 22.73)	1.40 (0.83, 2.37)	-	216	6.89 (4.04, 11.64)	0.96 (0.56, 1.66)	-
Proton pump inhibitor								
No	338	8.87 (6.24, 12.51)	1.00	-	937	6.45 (4.93, 8.42)	1.00	-
Yes	334	9.72 (6.93, 13.55)	1.02 (0.67, 1.56)	-	1066	7.51 (6.00, 9.38)	1.30 (0.92, 1.83)	-
Anticoagulants (warfarin and heparin)								
No	627	8.99 (6.96, 11.58)	1.00	-	1894	7.20 (6.04, 8.56)	1.00	-

Yes	45	13.39 (6.25, 27.41)	1.40 (0.66, 2.97)	-	109	3.77 (1.43, 9.73)	0.61 (0.24, 1.57)	-
Hormone replacement therapy (estrogen-only)								
No	666	9.38 (7.36, 11.92)	1.00	-	1991	6.95 (5.84, 8.26)	1.00	-
Yes	6	0.00 (0.00, 0.00)	0.56 (0.03, 9.11)	-	12	19.79 (5.21, 59.74)	3.56 (1.01, 12.50)	-
Endocrine therapy except estrogen for breast cancer and prostate cancer								
No	660	9.46 (7.42, 12.02)	1.00	-	1953	7.21 (6.07, 8.55)	1.00	-
Yes	12	0.00 (0.00, 0.00)	0.96 (0.19, 4.89)	-	50	0.00 (0.00, 0.00)	0.41 (0.08, 2.04)	-
Glucocorticoids mean dose[c]								
≥ 0, < 1, mg/day	632	9.05 (7.02, 11.63)	1.00	-	1869	6.96 (5.82, 8.32)	1.00	-
≥ 1, < 5, mg/day	19	16.92 (5.74, 44.09)	3.34 (1.48, 7.51)	-	73	7.50 (3.17, 17.17)	1.33 (0.60, 2.94)	-
≥ 5, < 7.5, mg/day	11	9.09 (1.33, 49.19)	1.14 (0.22, 5.86)	-	22	13.88 (4.69, 37.14)	2.59 (0.89, 7.56)	-
≥ 7.5	10	10.00 (1.47, 52.70)	1.99 (0.56, 7.11)	-	39	5.13 (1.31, 18.98)	0.93 (0.26, 3.28)	-
History of fractures other than VF								
No	594	8.45 (6.43, 11.06)	1.00	-	1702	7.01 (5.81, 8.44)	1.00	-
All hip fracture	8	25.00 (6.91, 68.52)	3.63 (1.01, 13.03)	-	54	12.21 (5.66, 25.28)	1.76 (0.79, 3.91)	-
NHNV fracture	64	14.42 (7.77, 25.91)	2.06 (1.16, 3.65)	-	225	6.15 (3.52, 10.65)	0.86 (0.49, 1.52)	-
Both[d]	6	16.67 (2.53, 72.69)	2.43 (0.47, 12.49)	-	22	4.55 (0.65, 28.13)	0.96 (0.19, 4.85)	-
No. of beds at medical institutions performing surgery								
≤ 19	53	21.22 (12.35, 35.07)	2.04 (1.09, 3.81)	-	39	2.78 (0.40, 18.13)	0.51 (0.10, 2.62)	-
20–199	291	10.08 (7.07, 14.28)	1.00	-	868	7.76 (6.03, 9.94)	1.00	-
200–399	65	10.87 (5.34, 21.47)	1.17 (0.59, 2.33)	-	586	5.58 (3.92, 7.91)	0.78 (0.52, 1.19)	-
≥ 400	263	5.60 (3.35, 9.29)	0.64 (0.38, 1.07)	-	510	7.76 (5.61, 10.69)	0.98 (0.65, 1.46)	-
missing	0	-	-	-	0	-	-	-
Pre-preoperative osteoporosis medications status								

Untreated[e]	399	7.84 (5.55, 11.03)	1.00	1.00	1277	6.48 (5.19, 8.08)	1.00	1.00
Previously treated	273	11.38 (8.09, 15.88)	1.57 (1.03, 2.41)	1.20 (0.75, 1.93)	726	7.99 (6.08, 10.45)	1.09 (0.77, 1.53)	1.05 (0.70, 1.57)
Drug classes for perioperative osteoporosis medications[f]								
No	118	6.22 (3.01, 12.63)	1.00	1.00	411	9.82 (7.09, 13.50)	1.00	1.00
Anabolic agents[g]	249	11.39 (7.95, 16.19)	2.09 (0.98, 4.46)	1.85 (0.85, 4.02)	721	5.46 (3.96, 7.51)	0.54 (0.35, 0.86)	0.52 (0.33, 0.84)
Antiresorptive agents	230	8.99 (5.89, 13.59)	1.96 (0.91, 4.23)	1.68 (0.75, 3.73)	574	8.13 (6.05, 10.88)	0.88 (0.57, 1.36)	0.84 (0.51, 1.36)
Combination of two or more[h]	4	25.00 (3.95, 87.21)	11.65 (2.78, 48.77)	12.06 (2.62, 55.43)	4	0.00 (0.00, 0.00)	1.16 (0.07, 19.41)	1.08 (0.06, 18.83)
Bone metabolism-regulating drugs only[i]	71	7.22 (3.07, 16.48)	1.42 (0.53, 3.85)	1.35 (0.50, 3.68)	293	5.22 (2.93, 9.23)	0.52 (0.28, 0.96)	0.51 (0.27, 0.94)

Using Firth's method for parameter estimates. CI, confidence interval; HR, hazard ratio; LSEHS, latter-stage elderly healthcare system; NE, not estimable; NHI, national health insurance; NHNV, non-hip non-vertebral; PVP, percutaneous vertebroplasty; SHI, society-managed employment-based health insurance association; VF, vertebral fracture

[a] Patients with chronic kidney disease, excluding dialysis

[b] Patients with mild liver disease, excluding moderate or severe liver disease

[c] As prednisolone equivalent mg/day. The mean dose per day calculated from the total dose per year in patients who were prescribed oral glucocorticoids for at least 90 days during the baseline period

[d] Patients with a history of both all hip fracture together with NHNV during the baseline period

[e] Untreated or bone metabolism-regulating drugs only

[f] "Anabolic agents" and "antiresorptive agents" also include patients who use those agents in combination with "bone metabolism-regulating drugs"

[g] Anabolic agents: PTH and Rmab

[h] Excluding bone metabolism-regulating drugs

[i] A general term that includes active vitamin D, vitamin K, and calcium

Supplemental Table 16 Subgroup analysis of factors associated with subsequent VF in the SFS group, by year of first spine surgery

	Index year							
	–2018				2019–			
Variable	N	Cumulative incidence % at Day 365 (95% CI)	Unadjusted HR (95% CI)	Adjusted HR (95% CI)	N	Cumulative incidence % at Day 365 (95% CI)	Unadjusted HR (95% CI)	Adjusted HR (95% CI)
Sex								
Male	145	8.41 (4.86, 14.34)	1.00	1.00	422	7.30 (4.97, 10.65)	1.00	1.00
Female	453	5.72 (3.90, 8.35)	0.61 (0.34, 1.06)	0.67 (0.38, 1.20)	1175	5.85 (4.55, 7.50)	0.82 (0.53, 1.26)	0.89 (0.56, 1.41)
Age, categories								
40–69, years	115	5.24 (2.39, 11,29)	1.00	1.00	132	3.90 (1.64, 9.13)	1.00	1.00
70–79, years	297	5.23 (3.18, 8.52)	0.92 (0.45, 1.87)	0.93 (0.45, 1.91)	687	6.66 (4.90, 9.00)	1.39 (0.64, 3.01)	1.37 (0.63, 2.98)
≥ 80, years	186	8.86 (5.52, 14.06)	1.08 (0.52, 2.26)	1.16 (0.54, 2.53)	778	6.28 (4.62, 8.51)	1.14 (0.52, 2.49)	1.17 (0.53, 2.58)
Type of insurance								
SHI	13	0.00 (0.00, 0.00)	0.35 (0.02, 5.95)	-	10	0.00 (0.00, 0.00)	0.52 (0.03, 9.06)	-
NHI	239	6.89 (4.28, 11.01)	1.00	-	302	4.99 (2.97, 8.30)	1.00	-
LSEHS	346	6.22 (4.10, 9.39)	0.81 (0.47, 1.38)	-	1285	6.59 (5.24, 8.28)	1.10 (0.66, 1.83)	-
Degenerative spine diseases								
No	162	7.64 (4.41, 13.06)	1.00	-	473	6.80 (4.71, 9.77)	1.00	-
Yes	436	5.91 (4.03, 8.62)	0.79 (0.44, 1.39)	-	1124	5.98 (4.63, 7.71)	0.87 (0.57, 1.34)	-
Hip or knee osteoarthritis								
No	538	6.70 (4.86, 9.21)	1.00	1.00	1401	6.60 (5.31, 8.18)	1.00	1.00
Yes	60	3.48 (0.88, 13.22)	0.56 (0.19, 1.67)	0.53 (0.17, 1.61)	196	3.53 (1.58, 7.78)	0.51 (0.23, 1.14)	0.57 (0.25, 1.28)
Hyperparathyroidism								

No	581	6.39 (4.65, 8.76)	1.00	-	1556	6.20 (5.02, 7.65)	1.00	-
Yes	17	5.88 (0.85, 34.98)	1.53 (0.43, 5.51)	-	41	6.93 (1.71, 25.81)	1.45 (0.50, 4.24)	-
Hypoparathyroidism								
No	598	6.38 (4.66, 8.70)	1.00	-	1596	6.23 (5.05, 7.67)	1.00	-
Yes	0	-	-	-	1	0.00 (0.00, 0.00)	6.16 (0.38, 101.21)	-
Hyperthyroidism								
No	582	6.55 (4.79, 8.93)	1.00	-	1555	6.33 (5.13, 7.80)	1.00	-
Yes	16	0.00 (0.00, 0.00)	0.31 (0.02, 5.15)	-	42	2.38 (0.34, 15.72)	0.95 (0.27, 3.34)	-
Hypothyroidism								
No	560	6.26 (4.51, 8.65)	1.00	-	1433	6.17 (4.95, 7.69)	1.00	-
Yes	38	8.05 (2.67, 22.94)	1.36 (0.51, 3.58)	-	164	6.68 (3.48, 12.65)	1.18 (0.64, 2.19)	-
Chronic kidney disease								
No	474	5.65 (3.88, 8.19)	1.00	-	1226	6.42 (5.06, 8.13)	1.00	-
Non-dialysis[a]	113	9.12 (5.01, 16.31)	1.50 (0.82, 2.76)	-	343	5.74 (3.64, 8.97)	0.89 (0.54, 1.47)	-
Dialysis	11	9.09 (1.33, 49.19)	1.58 (0.30, 8.25)	-	28	3.85 (0.55, 24.31)	0.86 (0.17, 4.41)	-
Diabetes mellitus								
No	360	7.41 (5.11, 10.70)	1.00	-	919	6.51 (4.96, 8.53)	1.00	-
Yes	238	4.77 (2.67, 8.45)	0.75 (0.43, 1.32)	-	678	5.83 (4.20, 8.08)	0.93 (0.62, 1.39)	-
Parkinson's disease								
No	555	5.96 (4.25, 8.32)	1.00	1.00	1495	5.72 (4.55, 7.17)	1.00	1.00
Yes	43	11.85 (5.11, 26.17)	2.10 (0.97, 4.57)	2.07 (0.91, 4.70)	102	13.54 (8.06, 22.25)	2.38 (1.33, 4.23)	2.04 (1.13, 3.69)
Hypertension								
No	177	5.70 (3.11, 10.33)	1.00	-	361	6.57 (4.24, 10.09)	1.00	-
Yes	421	6.68 (4.63, 9.60)	0.95 (0.54, 1.68)	-	1236	6.11 (4.81, 7.74)	0.87 (0.56, 1.38)	-

Dyslipidemia								
No	259	6.67 (4.20, 10.52)	1.00	-	645	7.36 (5.43, 9.94)	1.00	-
Yes	339	6.16 (4.02, 9.39)	0.97 (0.57, 1.66)	-	952	5.45 (4.08, 7.27)	0.75 (0.50, 1.11)	-
Myocardial infarction								
No	579	6.58 (4.81, 8.97)	1.00	-	1541	6.33 (5.12, 7.81)	1.00	-
Yes	19	0.00 (0.00, 0.00)	0.27 (0.02, 4.51)	-	56	3.57 (0.91, 13.53)	1.02 (0.35, 2.99)	-
Congestive heart failure								
No	414	6.22 (4.24, 9.07)	1.00	-	1048	5.90 (4.53, 7.67)	1.00	-
Yes	184	6.72 (3.87, 11.54)	1.03 (0.58, 1.82)	-	549	6.87 (4.86, 9.66)	1.20 (0.79, 1.81)	-
Cerebrovascular disease								
No	462	6.94 (4.93, 9.73)	1.00	-	1097	5.84 (4.51, 7.55)	1.00	-
Yes	136	4.49 (2.04, 9.73)	0.94 (0.50, 1.77)	-	500	7.10 (4.97, 10.11)	1.01 (0.65, 1.55)	-
Dementia								
No	555	6.87 (5.02, 9.36)	1.00	-	1426	6.25 (5.01, 7.78)	1.00	-
Yes	43	0.00 (0.00, 0.00)	0.35 (0.07, 1.81)	-	171	5.96 (3.11, 11.25)	0.93 (0.48, 1.83)	-
Chronic pulmonary disease								
No	421	6.36 (4.37, 9.20)	1.00	-	1136	6.92 (5.47, 8.74)	1.00	-
Yes	177	6.42 (3.61, 11.31)	1.17 (0.67, 2.06)	-	461	4.51 (2.85, 7.09)	0.76 (0.48, 1.21)	-
Liver disease								
No	459	6.30 (4.39, 9.00)	1.00	-	1137	6.75 (5.32, 8.53)	1.00	-
Mild[b]	135	6.75 (3.57, 12.58)	1.16 (0.63, 2.12)	-	449	5.02 (3.21, 7.82)	0.75 (0.47, 1.20)	-
Moderate or severe	4	0.00 (0.00, 0.00)	1.98 (0.12, 33.61)	-	11	0.00 (0.00, 0.00)	0.75 (0.05, 12.35)	-
Rheumatologic disease								
No	511	6.46 (4.61, 9.02)	1.00	-	1407	5.92 (4.69, 7.44)	1.00	-

Yes	87	5.91 (2.50, 13.63)	0.63 (0.26, 1.53)	-	190	8.49 (5.19, 13.74)	1.61 (0.96, 2.71)	-
Malignant tumor								
No	529	5.44 (3.79, 7.79)	1.00	1.00	1355	5.86 (4.64, 7.39)	1.00	1.00
Yes	69	13.71 (7.36, 24.74)	2.20 (1.14, 4.23)	2.30 (1.16, 4.56)	242	8.37 (5.18, 13.38)	1.36 (0.81, 2.29)	1.31 (0.77, 2.24)
Charlson Comorbidity Index								
0–2	364	5.08 (3.23, 7.94)	1.00	-	879	6.14 (4.62, 8.13)	1.00	-
≥ 3	234	8.43 (5.45, 12.90)	1.56 (0.92, 2.64)	-	718	6.33 (4.63, 8.62)	1.05 (0.70, 1.56)	-
Psychotropic drugs								
No	238	6.90 (4.28, 11.02)	1.00	-	647	5.47 (3.85, 7.74)	1.00	-
Yes	360	6.03 (3.97, 9.10)	0.78 (0.46, 1.32)	-	950	6.74 (5.19, 8.73)	1.24 (0.82, 1.87)	-
Narcotics								
No	586	6.50 (4.75, 8.87)	1.00	-	1588	6.26 (5.08, 7.71)	1.00	-
Yes	12	0.00 (0.00, 0.00)	0.45 (0.03, 7.53)	-	9	0.00 (0.00, 0.00)	0.79 (0.05, 12.88)	-
Anticonvulsants								
No	291	7.13 (4.66, 10.84)	1.00	-	848	6.13 (4.57, 8.19)	1.00	-
Yes	307	5.66 (3.56, 8.95)	0.75 (0.44, 1.27)	-	749	6.31 (4.67, 8.50)	0.96 (0.65, 1.43)	-
Muscle relaxants								
No	502	6.19 (4.37, 8.74)	1.00	-	1399	6.14 (4.90, 7.67)	1.00	-
Yes	96	7.35 (3.57, 14.80)	1.47 (0.78, 2.78)	-	198	6.76 (3.76, 12.01)	1.09 (0.61, 1.93)	-
Proton pump inhibitor								
No	229	5.82 (3.42, 9.81)	1.00	-	627	6.11 (4.35, 8.55)	1.00	-
Yes	369	6.72 (4.56, 9.87)	1.29 (0.74, 2.27)	-	970	6.29 (4.82, 8.19)	1.01 (0.67, 1.52)	-
Anticoagulants (warfarin and heparin)								
No	550	6.55 (4.75, 9.01)	1.00	-	1472	6.09 (4.88, 7.59)	1.00	-

Yes	48	4.47 (1.13, 16.78)	0.55 (0.15, 1.98)	-	125	7.91 (4.13, 14.88)	1.50 (0.81, 2.78)	-
Hormone replacement therapy (estrogen-only)								
No	593	6.43 (4.70, 8.77)	1.00	-	1590	6.25 (5.07, 7.70)	1.00	-
Yes	5	0.00 (0.00, 0.00)	0.93 (0.06, 15.36)	-	7	0.00 (0.00, 0.00)	0.96 (0.06, 15.66)	-
Endocrine therapy except estrogen for breast cancer and prostate cancer								
No	592	6.27 (4.56, 8.59)	1.00	-	1560	6.22 (5.03, 7.68)	1.00	-
Yes	6	16.67 (2.53, 72.69)	4.90 (1.36, 17.61)	-	37	5.84 (1.49, 21.50)	1.25 (0.35, 4.41)	-
Glucocorticoids mean dose[c]								
≥ 0, < 1 mg/day	521	6.74 (4.86, 9.31)	1.00	-	1454	6.06 (4.85, 7.56)	1.00	-
≥ 1, < 5, mg/day	34	3.03 (0.43, 19.63)	0.45 (0.09, 2.33)	-	78	10.20 (4.96, 20.34)	1.68 (0.79, 3.57)	-
≥ 5, < 7.5, mg/day	18	5.56 (0.80, 33.36)	0.86 (0.16, 4.48)	-	33	6.06 (1.55, 22.12)	2.22 (0.75, 6.54)	-
≥ 7.5, mg/day	25	4.00 (0.57, 25.16)	0.98 (0.27, 3.56)	-	32	3.13 (0.45, 20.18)	1.68 (0.57, 4.99)	-
History of fractures other than VF								
No	507	6.90 (4.98, 9.53)	1.00	-	1302	6.30 (5.00, 7.93)	1.00	-
All hip fracture	16	0.00 (0.00, 0.00)	0.28 (0.02, 4.77)	-	35	2.86 (0.41, 18.60)	1.14 (0.32, 4.08)	-
NHNV fracture	73	4.44 (1.45, 13.18)	0.62 (0.23, 1.64)	-	237	6.53 (3.81, 11.06)	1.04 (0.60, 1.81)	-
Both[d]	2	0.00 (0.00, 0.00)	2.20 (0.13, 37.45)	-	23	4.35 (0.62, 27.07)	1.06 (0.21, 5.44)	-
No. of beds at medical institutions performing surgery								
≤ 19	14	14.29 (3.78, 46.06)	2.99 (0.86, 10.41)	-	16	7.14 (1.04, 40.92)	2.15 (0.37, 12.30)	-
20–199	106	4.72 (1.99, 10.96)	1.00	-	262	3.40 (1.58, 7.22)	1.00	-
200–399	133	3.83 (1.61, 8.96)	0.98 (0.41, 2.36)	-	551	7.17 (5.12, 10.00)	2.02 (0.99, 4.14)	-
≥ 400	345	7.56 (5.17, 10.99)	1.12 (0.54, 2.33)	-	768	6.46 (4.84, 8.59)	1.79 (0.89, 3.62)	-
missing	0	-	-	-	0	-	-	-
Details of the surgical procedure								

Posterior spinal fusion	398	5.98 (4.01, 8.87)	1.00	1.00	1137	5.38 (4.10, 7.03)	1.00	1.00
Anterior spinal fusion[e]	193	6.89 (4.06, 11.58)	1.75 (1.02, 3.01)	1.86 (1.06, 3.26)	379	8.84 (6.22, 12.49)	1.73 (1.12, 2.66)	1.58 (1.02, 2.46)
Combined PVP	7	14.29 (2.14, 66.59)	5.04 (1.36, 18.64)	3.50 (0.84, 14.57)	81	5.89 (2.23, 15.08)	1.52 (0.67, 3.43)	1.39 (0.61, 3.18)
Length of fusion area								
1 level	36	2.78 (0.40, 18.13)	0.37 (0.07, 1.93)	0.47 (0.09, 2.51)	65	0.00 (0.00, 0.00)	0.31 (0.06, 1.59)	0.38 (0.07, 2.01)
2 levels	113	4.52 (1.91, 10.53)	0.89 (0.45, 1.76)	1.09 (0.53, 2.22)	315	5.36 (3.17, 9.01)	0.70 (0.40, 1.21)	0.76 (0.44, 1.34)
≥ 3 levels	449	7.14 (5.08, 10.01)	1.00	1.00	1217	6.79 (5.40, 8.51)	1.00	1.00
Pre-preoperative osteoporosis medications status								
Untreated[f]	279	4.79 (2.81, 8.12)	1.00	-	780	6.19 (4.59, 8.32)	1.00	-
Previously treated	319	7.74 (5.25, 11.33)	1.41 (0.82, 2.42)	-	817	6.25 (4.66, 8.37)	1.02 (0.68, 1.51)	-
Drug classes for perioperative osteoporosis medications[g]								
No	115	3.54 (1.34, 9.16)	1.00	1.00	301	6.87 (4.34, 10.77)	1.00	1.00
Anabolic agents[h]	273	6.78 (4.32, 10.55)	1.06 (0.51, 2.21)	0.95 (0.45, 2.02)	728	7.22 (5.43, 9.56)	1.16 (0.68, 1.99)	1.11 (0.64, 1.92)
Antiresorptive agents	156	8.67 (5.13, 14.47)	1.26 (0.58, 2.76)	1.28 (0.58, 2.84)	391	5.33 (3.35, 8.43)	0.97 (0.52, 1.81)	1.07 (0.56, 2.03)
Combination of two or more[i]	1	0.00 (0.00, 0.00)	4.81 (0.26, 87.72)	2.92 (0.12, 69.33)	3	33.33 (5.48, 94.59)	9.41 (1.74, 50.84)	11.71 (2.05, 66.95)
Bone metabolism-regulating-drugs only[j]	53	3.77 (0.96, 14.26)	0.71 (0.21, 2.44)	0.64 (0.18, 2.23)	174	2.33 (0.73, 7.32)	0.52 (0.20, 1.36)	0.54 (0.21, 1.43)

Using Firth's method for parameter estimates. CI, confidence interval; HR, hazard ratio; LSEHS, latter-stage elderly healthcare system; NHI, national health insurance; NHNV, non-hip non-vertebral; PVP, percutaneous vertebroplasty; SFS, spine fusion surgery; SHI, society-managed employment-based health insurance association; VF, vertebral fracture

[a] Patients with chronic kidney disease, excluding dialysis

[b] Patients with mild liver disease, excluding moderate or severe liver disease

[c] As prednisolone equivalent mg/day. The mean dose per day calculated from the total dose per year in patients who were prescribed oral glucocorticoids for at least 90 days during the baseline period

[d] Patients with a history of both all hip fracture together with NHNV during the baseline period

[e] Including combined antero-posterior approaches

[f] Untreated or bone metabolism-regulating drugs only

[g] "Anabolic agents" and "antiresorptive agents" also include patients who use those agents in combination with "bone metabolism-regulating drugs"

[h] Anabolic agents: PTH and Rmab

[i] Excluding bone metabolism-regulating drugs

[j] A general term that includes active vitamin D, vitamin K, and calcium

Supplemental Fig. 1 Diagram of study design

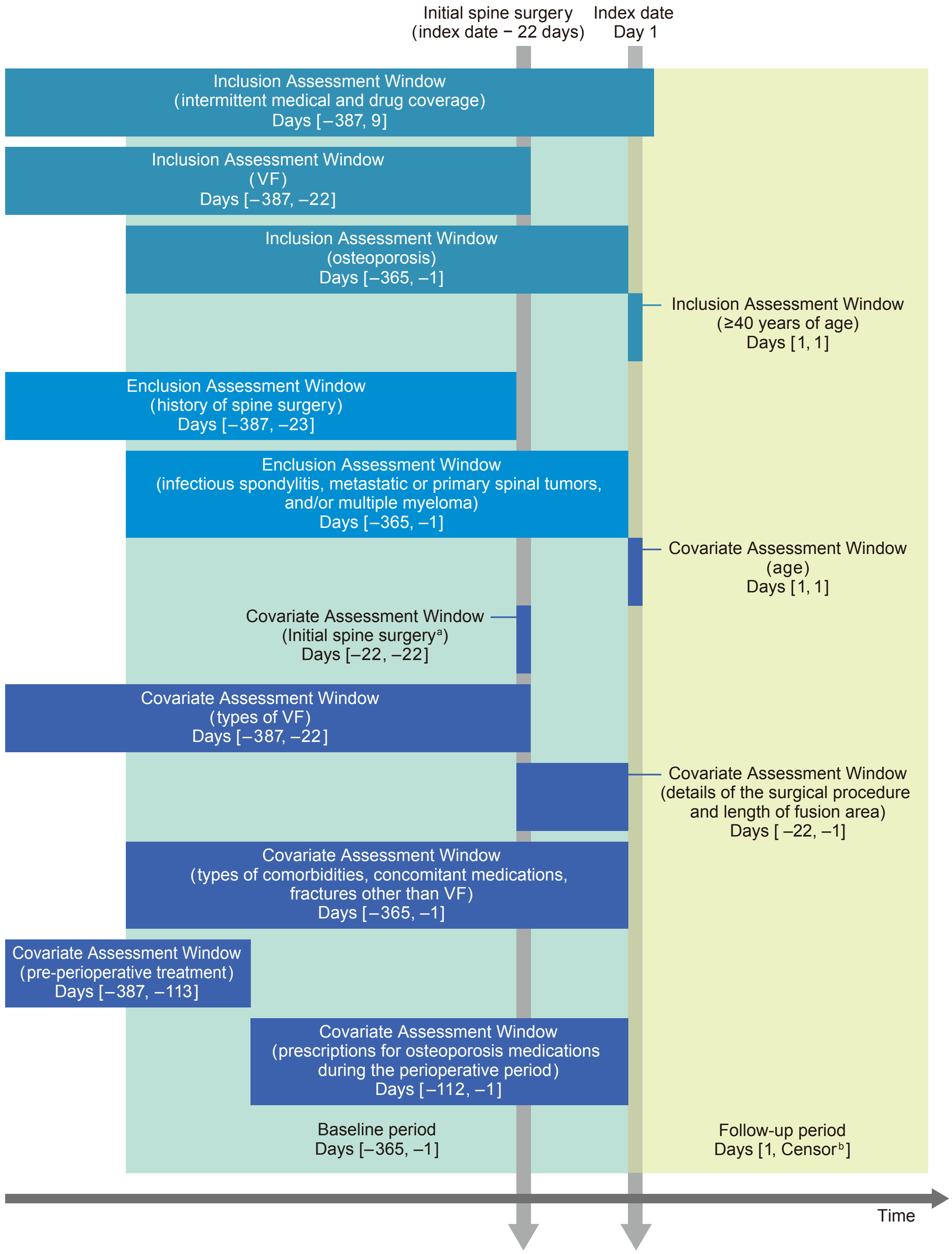

VF, vertebral fracture

[a] Year of initial spine surgery, number of hospital beds at the medical institution where the spine surgery was performed, etc

[b] Time to outcome (the earliest of the following: date of outcome onset, 1095 days after index date, last day of the last month of the observation period

Supplemental Fig. 2 Medication persistence in patients who first started PTH or Rmab in the perioperative period

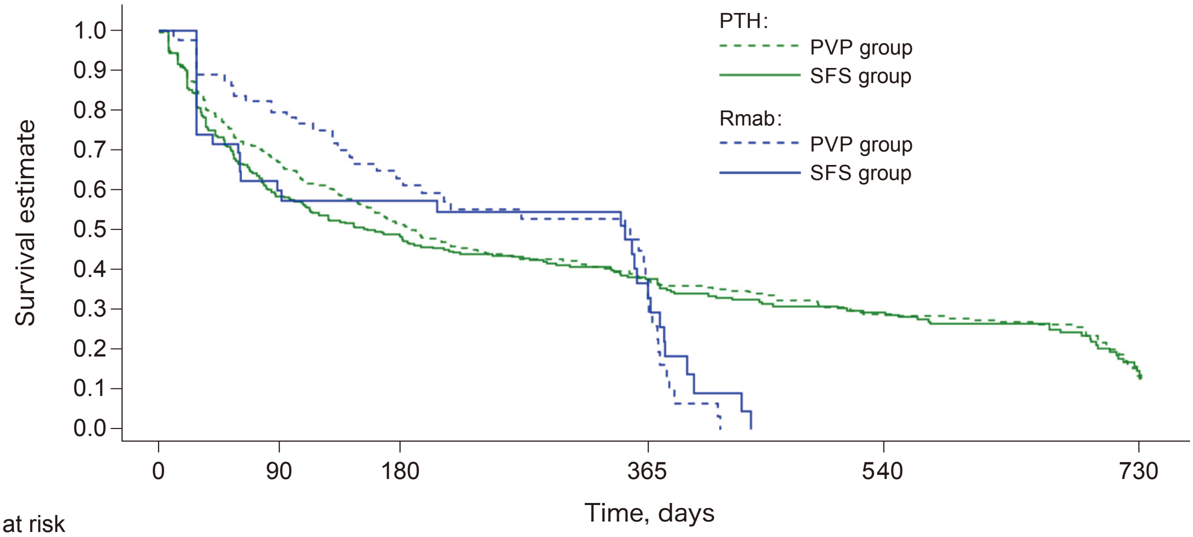

Number at risk

		0	90	180	365	540	730
PTH:	PVP group	412	260	179	108	61	22
	SFS group	345	185	139	82	52	14
Rmab:	PVP group	81	57	34	10	0	0
	SFS group	46	24	21	9	0	0

Survival estimate (95%CI)	Medication persistence, days 90	180	365	540	730
PTH: PVP group	0.6669 (0.6186, 0.7105)	0.5154 (0.4642, 0.5642)	0.3732 (0.3227, 0.4235)	0.2893 (0.2398, 0.3405)	0.1282 (0.0867, 0.1782)
SFS group	0.5847 (0.5299, 0.6354)	0.4823 (0.4268, 0.5356)	0.3773 (0.3219, 0.4324)	0.2932 (0.2386, 0.3497)	0.1371 (0.0872, 0.1982)
Rmab: PVP group	0.7954 (0.6875, 0.8695)	0.6303 (0.5026, 0.7337)	0.2908 (0.1595, 0.4358)	0.0000 NE	0.0000 NE
SFS group	0.5987 (0.4401, 0.7257)	0.5738 (0.4151, 0.7040)	0.3302 (0.1781, 0.4908)	0.0000 NE	0.0000 NE

CI, confidence interval; NE, not estimable; PTH, parathyroid hormone; PVP, percutaneous vertebroplasty; Rmab, romosozumab; SFS, spine fusion surgery
